# PanDelos-plus: A parallel algorithm for computing sequence homology in pangenomic analysis

**DOI:** 10.1371/journal.pcbi.1014724

**Published:** 2026-09-17

**Authors:** Simone Colli, Emiliano Maresi, Vincenzo Bonnici

**Affiliations:** Department of Mathematical, Physical and Computer Sciences, University of Parma, Parma, Italy; Hong Kong University of Science and Technology School of Science, HONG KONG

## Abstract

The identification of homologous gene families across multiple genomes is a central task in bacterial pangenomics traditionally requiring computationally demanding all-against-all comparisons. PanDelos addresses this challenge with an alignment-free and parameter-free approach based on k-mer profiles, combining high speed, ease of use, and competitive accuracy with state-of-the-art methods. However, the increasing availability of genomic data requires tools that can scale efficiently to larger datasets. To address this need, we present PanDelos-plus, a fully parallel, gene-centric redesign of PanDelos. The algorithm parallelizes the most computationally intensive phases (Best Hit detection and Bidirectional Best Hit extraction) through data decomposition and a thread pool strategy, while employing lightweight data structures to reduce memory usage. Benchmarks on synthetic datasets show that PanDelos-plus achieves up to 14x faster execution and reduces memory usage by up to 96%, while maintaining consistency with the original algorithm. These improvements allow the PanDelos methodology to be applied to population-scale comparative genomics, thus enabling more precise characterisation of pangenome structure and dynamics.

PanDelos-plus is available at github.com/synbionics/PanDelos-plus.

## Author summary

The identification of homologous gene families across multiple genomes requires computationally demanding all-against-all comparisons. Recent solutions based on k-mer profiles have shown that competitive accuracy can be achieved with state-of-the-art methods, while being faster and easier to use. Among these, PanDelos stands out for its alignment-free and parameter-free approach, combining high speed and ease of use. However, the increasing availability of genomic data requires tools that can scale efficiently to larger datasets. To address this need, we developed PanDelos-plus, a fully parallel, gene-centric redesign of PanDelos. This new version of the algorithm parallelizes the most computationally intensive phases through data decomposition and a thread pool strategy, while employing lightweight data structures to reduce memory usage. Benchmarks show that PanDelos-plus outperforms the original version in terms of speed and memory usage. These improvements allow substantially larger bacterial genome collections to be analyzed on standard multicore workstations, lowering computational barriers and facilitating scalable alignment-free gene-focused prokaryotic pangenome reconstruction.

## Introduction

With the rapid progress of high-throughput sequencing projects, biological data is growing exponentially, creating a need for efficient and scalable algorithms for further analysis [1]. This need is particularly evident in pangenomics, a discipline that has emerged to study the entire repertoire of gene families in genomes of a given clade. In order to understand the genetic diversity within a species, an abstract structure called pangenome is used. A pangenome is the collection of all gene families present in a set of genomes, divided into core, dispensable, and singleton genes. Its computational reconstruction is achieved by identifying groups of homologous genes [2–4]. Core gene families, shared by all genomes, encode essential cellular functions. Dispensable families occur in only a subset of genomes and often provide adaptive advantages in specific environments. Singleton families, unique to a single genome, usually arise from horizontal gene transfer or reflect organism-specific adaptations [2]. It is important to note that the classification of gene families into core, dispensable, and singleton categories is inherently dependent on the set of genomes under consideration. As additional genomes from the same phylogenetic group are incorporated, gene families previously classified as singletons may be reclassified as dispensable if homologous copies are detected in newly sampled genomes. Thus, these categories represent properties of the sampled collection rather than fixed biological attributes of a species. This sampling dependence is closely related to the distinction between open and closed pangenomes. In species with an open pangenome, the number of novel gene families continues to increase as additional genomes are sequenced, whereas in closed pangenomes the discovery rate progressively saturates [2,5]. Scalable pangenome reconstruction methods are therefore essential not only for computational efficiency, but also for accurately characterizing how gene family frequencies evolve as genome sampling expands.

The importance of pangenome information is revealed in terms of clinical applications. Pangenomic studies have been used to identify vaccine and antibacterial targets, to detect strain-specific virulence factors, to distinguish lineage- and niche-specific bacterial populations, and to investigate pathogens in epidemic diseases [6–10].

Approaches to pangenome content discovery should take into account that gene sequences accumulate mutations during replication and evolution, leading to sequence polymorphisms such as nucleotide substitutions, insertions, and deletions [11–14]. These mutations complicate the accurate identification and clustering of homologous genes [15]. Computationally, retrieval of a pangenome is an NP-hard problem [16], mainly due to all-against-all comparisons between gene sets. While the pangenome itself is a biologically defined set of gene families present in a population, in practice it can only be characterized through computational reconstruction from genomic data, which makes the task complex and time-consuming. To address this challenge, many tools have been developed using combinations of techniques, algorithms, and thresholds to address the challenge of pangenome reconstruction, each with distinct methodologies for gene clustering, leading to different trade‑offs in terms of speed, accuracy, and scalability. Among the widely used solutions are Roary [17], Panaroo [18], PPanGGOLiN [19], PanDelos [15], and Panakeia [20]. More recently, the field has seen the introduction of additional tools designed to improve scalability and efficiency for large genomic collections, such as PanTA, a progressive pangenome inference framework optimized for very large datasets [21], and ongoing updates to existing frameworks like PPanGGOLiN (with recent releases in 2025). Roary remains recognized for its speed on small to moderate, high‑quality genomes, but its clustering approach is sensitive to assembly errors and may inflate dispensable counts under fragmented assemblies. Panaroo extends this by including graph‑based error correction, making it more robust to draft input genomes. PPanGGOLiN employs a probabilistic graph partitioning strategy to classify core, dispensable, and singleton gene families. Panakeia emphasizes structural and syntenic pattern analysis within pangenomes, generating richer graph representations that capture gene order and genomic context, which allows more detailed biological interpretation beyond simple presence/absence matrices. Tools like PanTA explicitly target scalability by progressively building the pangenome and avoiding rebuilding from scratch as sample counts grow, demonstrating good resource efficiency on collections of hundreds to thousands of genomes. In addition to these, other frameworks like PanTools v3 [22] have broadened pangenome analysis by integrating functional annotation, classification, and phylogenomic features into the pangenome graph representation, though they may emphasize downstream analyses over core clustering performance. Graph‑centric toolkits and browsers such as PPanG (for interactive graph inspection) and various graph‑based methods (e.g., variation‑graph strategies) have also emerged, reflecting a growing emphasis on richer representations of genomic variation beyond presence–absence matrices [23,24].

Comparisons demonstrated that PanDelos achieves competitive accuracy relative to other tools across a range of conditions [25]. However, it is not fully optimised for very large datasets: it relies on single‑threaded algorithms and employs memory‑intensive data structures, making the analysis of large collections time‑ and resource‑intensive.

Here, we introduce PanDelos-plus, a re-engineered, parallelized version of PanDelos designed for high-throughput pangenomic analysis. This work leverages a multi-threading approach and optimized, low-memory data structures to enable rapid and efficient analysis overcoming the scalability limitations of PanDelos. PanDelos-plus operates on pre-annotated gene sets and requires as input collections of predicted nucleotide or amino acid sequences for each genome, generated by external genome annotation pipelines. It does not perform genome assembly, gene prediction, or genome reconstruction. However, it can be substituted for the original PanDelos algorithm within the extended PanDelos-frags [26] pipeline that is specifically designed to deal with fragmented, unannotated genomes.

We evaluated PanDelos-plus on both real dataset (including *Escherichia coli*, *Salmonella enterica*, *Mycoplasma* and *Xanthomonas campestris*) and synthetic collections generated with PANPROVA [27] (a tool that simulates prokaryotic pangenome evolution starting from a complete ancestral genome) comprising up to 600 genomes. Results show up to a 14x speedup and a memory reduction of up to 96% compared to PanDelos, while producing results consistent with those of the original PanDelos algorithm. Such an evaluation also shows how extending the set of genomes that are included in a pangenomic analysis can substantially alter the result in terms of gene family composition and, in particular, in identifying core, dispensable and singleton genes. Therefore, PanDelos-plus enables a more precise characterization of pangenome structure and dynamics, which is particularly important for species with open pangenomes, where gene discovery continues to increase with sampling.

### Basic notions

Given a dataset consisting of a list of *n* genomes $𝔾=\{G_{1},\;G_{2},...,\;G_{n}\}$, each genome $G_{i}$ is represented by a list of $m_{i}$ genes. $G_{i}=\{g_{i,1},g_{i,2},...,g_{i,m_{i}}\}$, where $m_{i}=|G_{i}|$ denotes the number of genes in genome $G_{i}$.

A gene $g_{i,j}\in \Gamma^{*}$ is a string $s=a_{1}a_{2}...a_{n}$ constructed over a nucleotide or amino acid alphabet Γ. The substring of *s* starting at position *y* and ending at position *w* is denoted by *s*[*y*, *w*], where 1≤y≤w≤n.

Given a fixed value *k* it’s possible to identify a k-mer, defined as a string $\alpha \in \Gamma^{*}$ such that |α|=k. Considering a gene *g* represented by the sequence *s*, the length of *s* denoted as |*s*| corresponds to the number of characters in the sequence. Within the sequence *s*, there are exactly |s|−k+1 k-mers.

Given a fixed integer *k*, a k-mer is a string $\alpha \in \Gamma^{*}$ with |α|=k. Let *g* be a gene represented by a sequence *s* of length |*s*|.

For a fixed length *k*, the k-mer dictionary of a gene *g*, represents all k-mers contained inside the gene, and is defined as:


$$\begin{array}{c} D_{k}(g)=\{\alpha \in \Gamma^{*}:\exists \rho \text{ such that }g[\rho ,\;\rho +k-1]=\alpha ,\;1\leq \rho \leq |g|-k+1\}. \end{array}$$ (1)

Each k-mer can appear multiple times within a gene *g*. The set of occurrences of a k-mer α in *g* is:


$$\begin{array}{c} occ_{g}(\alpha )=\{\rho :1\leq \rho \leq |g|-k+1\wedge g[\rho ,\;\rho +k-1]=\alpha \}. \end{array}$$ (2)

The multiplicity of a k-mer α in a gene *g*, denoted $mult_{g}(\alpha )$, is the total number of occurrences of α in *g*:


$$\begin{array}{c} mult_{g}(\alpha )=|occ_{g}(\alpha )|. \end{array}$$ (3)

The dictionary-based approach relies on the k-mers, which are highly sensitive to the length *k* of the words that compose the dictionary. The best resolution of *k* in pangenomic analysis is theoretically shown to be the best as follow by the formula:


$$\begin{array}{c} k=\log_{\left|\Gamma \right|}\sum_{i=1}^{n}|G_{i}| \end{array}$$ (4)

where $G_{i}$, is the i-th genome of the dataset 𝔾, |Γ| is the size of the alphabet that is considered, and *n* is the number of genomes in the dataset. This formula provides the value of *k* that maximizes the resolution of genome content, i.e., it balances uniqueness and coverage of k-mers across the dataset, enabling accurate discrimination of gene families and homologous regions. Gene length and GC content are not directly included because the metric is based on the total sequence information in the dataset and the alphabet size, which together capture sequence complexity. These definitions and rationale follow the studies reported in [28,29] that defined the criterion for finding the right word length for comparing a single genome to a random one. Such a criterion was extended to pangenomes in [15] in an empirical way, whose result was confirmed in an extensive comparison study [25].

To limit the number of pairwise comparisons and focus the computation on plausible homologous candidates, PanDelos-plus applies two pre-filtering steps prior to the computation of pairwise similarity: a length-based filter and a coverage-based filter inherited from PanDelos. Only gene pairs passing these filters are subsequently evaluated using the generalized Jaccard similarity.

For each pair of genes $g_{1}\in G_{i}$ and $g_{2}\in G_{j}$, the sequence similarity is computed using the generalized Jaccard similarity on k-mer multiplicity, defined as:


$$\begin{array}{c} J_{k}(g_{1},g_{2})=\frac{\sum_{w\in D_{k}(g_{1})\cap D_{k}(g_{2})}min(mult_{g_{1}}(w),mult_{g_{2}}(w))}{\sum_{w\in D_{k}(g_{1})\cup D_{k}(g_{2})}max(mult_{g_{1}}(w),mult_{g_{2}}(w))} \end{array}$$ (5)

where $D_{k}(g_{1})$ and $D_{k}(g_{2})$ are the k-mer dictionaries (as defined in Eq (1)) of the genes *g*_1_ and *g*_2_, respectively, multiplicities $mult_{g_{1}}(w)$ and $mult_{g_{2}}(w)$ are the respective counts of the k-mer *w* in each gene (as defined in Eq (3)) and *min* and *max* are the standard minimum and maximum functions between the two values.

There is no theory that defines a non-empirical threshold to be applied to the Jaccard similarity in the context of pangenomic analysis, thus we introduce a filtering criterion based on the relative length of the gene pairs under consideration. The filtering criterion is based on a length-based threshold θ, such that 0≤θ≤1, that is applied to the gene pairs to be compared. Given two genes $g_{i}$ and $g_{j}$, they are considered similar enough to be compared if


$$\begin{array}{c} \left|g_{i}|\geq \theta \cdot |g_{j}|\;and\;|g_{j}|\geq \theta \cdot |g_{i}\right| \end{array}$$ (6)

where $\left|g_{i}\right|$ and $\left|g_{j}\right|$ are the lengths of the genes $g_{i}$ and $g_{j}$, respectively.

By default, we set θ=0.5, corresponding to allowing gene length differences of up to a factor of two; however, θ can be specified by the user to accommodate different levels of stringency depending on the application.

This length-based threshold θ is applied to exclude gene pairs that are considered too dissimilar in size, under the assumption that highly unbalanced lengths imply low sequence similarity.

To ensure that similarity is not only high but also reciprocal, we adopt an additional filtering step based on the dictionary intersection coverage, defined as:


$$\begin{array}{c} p_{k}(s\to t)=\frac{\sum_{w\in D_{k}(s)\cap D_{k}(t)}{mult}_{s}(w)}{|s|-k+1}. \end{array}$$ (7)

where $D_{k}(s)$ and $D_{k}(t)$ denote the k-mer dictionaries of sequences *s* and *t*, respectively, $D_{k}(s)\cap D_{k}(t)$ is the set of k-mers shared by both sequences, and ${mult}_{s}(w)$ is the multiplicity of k-mer *w* in sequence *s* (as defined in Eq. (3)).

Intuitively, $p_{k}(s\to t)$ represents the fraction of all k-mer occurrences in *s* that correspond to k-mers also present in *t*, and thus quantifies the propor*t*ion of the k-mer content of *s* that is covered by *t*.

Two genes $g_{i}$ and $g_{j}$ are considered homologous candidates only if both directional coverages exceed a threshold τ: $p_{k}(g_{i}\to g_{j})\geq \tau \;\wedge \;p_{k}(g_{j}\to g_{i})\geq \tau$.

Here, τ∈[0,1] is a coverage threshold controlling the minimum fraction of k-mer occurrences in each gene that must be shared with the other gene. This bidirectional constraint ensures that the overlap in k-mer content between two genes is sufficiently high in both directions. Following the theoretical rationale introduced in PanDelos, where the threshold is fixed to 2/*k* to enforce a minimum overlap between consecutive k-mers and to avoid matches driven by sparse, non-overlapping k-mer occurrences [15], we set the coverage threshold in PanDelos-plus to τ=2/k.

To identify homologous genes between two genomes, $G_{i}$ and $G_{j}$, the Jaccard similarity is computed for all ordered pairs of their respective genes. For a gene $g_{i,l}\in G_{i}$, its Best Hit in genome $G_{j}$ is defined as the set of genes in $G_{j}$ that maximize the Jaccard similarity from $g_{i,l}$ to genes in $G_{j}$. Formally:


$$\begin{array}{c} BH(g_{i,l},G_{j})=\{g_{j,z}\in G_{j}:J_{k}(g_{i,l},g_{j,z})=max_{g_{j,z}\in G_{j}}J_{k}(g_{i,l},g_{j,z})\} \end{array}$$ (8)

where $g_{i,l}$ is the *l*-th gene of the genome $G_{i}$, $g_{j,z}$ is the *z*-th gene of the genome $G_{j}$, and $J_{k}(g_{i,l},g_{j,z})$ is the Jaccard similarity between these two genes.

The Bidirectional Best Hit (BBH) is defined as a reciprocal Best Hit relation between genes in two genomes:


$$\begin{array}{c} BBH(g_{i,l},G_{j})=\{g_{j,k}\in G_{j}:g_{j,k}\in BH(g_{i,l},G_{j})\wedge g_{i,l}\in BH(g_{j,k},G_{i})\} \end{array}$$ (9)

where $g_{i,l}$ is the *l*-th gene of the genome $G_{i}$, $g_{j,k}$ is the *k*-th gene of the genome $G_{j}$, and $BH(g_{i,l},G_{j})$ and $BH(g_{j,k},G_{i})$ are the Best Hits (as defined in Eq (8)).

The BBH relation defines reciprocal similarity maxima between genes across two genomes and may involve multiple reciprocal matches for a given gene. The resulting BBH structure naturally forms a gene–gene similarity graph, in which edges encode reciprocal best-hit relationships across genomes. This graph-based representation is subsequently exploited by the network-based refinement step, where coherent gene families are extracted via community detection.

Based on the relationships established by the Bidirectional Best Hits (as defined in Eq (9)), genes can be clustered into groups of homologs, referred to as gene families. Let ℱ be a gene family, defined as a set of genes $ℱ\subset {⋃}_{G\in 𝔾}G$. We define the diffusivity of a gene family, denoted as δ(ℱ), as the number of distinct genomes in the dataset 𝔾 that contain at least one gene belonging to ℱ. Formally, the diffusivity is defined as:


$$\begin{array}{c} \delta (ℱ)=|\{G_{i}\in 𝔾:G_{i}\cap ℱ\neq \emptyset \}| \end{array}$$ (10)

where 𝔾 is the set of all genomes and $G_{i}\cap ℱ\neq \emptyset$ implies that the genome $G_{i}$ contributes at least one gene to the family ℱ. Consequently, the value of diffusivity ranges from 1 (the family is unique to a single genome) to *n* (the family is part of the core genome, present in all samples).

### Methodology

PanDelos-plus is a parallelized version of PanDelos, designed to enhance the scalability of the pangenomic analysis process. The proposed algorithm compares genomes by evaluating similarity between individual genes, treating each gene as a basic unit of comparison. This gene-centric approach enables the inference of homology relationships through pairwise gene comparisons, which can be efficiently parallelized. The most computationally demanding steps are the Best Hit detection and Bidirectional Best Hit extraction, which are designed to leverage parallel computing via data decomposition. Parallelization is implemented using a thread pool: the workload is partitioned such that each unit of work corresponds to a whole row of comparisons (i.e., one gene compared against the entire target genome), and threads iteratively process available tasks from the pool until all computations are completed.

Both PanDelos and PanDelos-plus operate on pre-annotated genomes represented as collections of gene sequences. The input consists of sets of nucleotide or amino acid sequences corresponding to predicted genes for each genome. The method does not perform genome assembly, gene prediction, or genome reconstruction from raw sequencing reads. Consequently, users must supply annotated gene sequences generated by external genome annotation pipelines prior to pangenome analysis.

Annotated gene sequences can be obtained through standard genome assembly and annotation workflows. PanDelos-plus is designed as a comparative genomics method operating on top of these preprocessing steps.

The input genomes may derive from complete assemblies, draft assemblies, or metagenome-assembled genomes (MAGs), provided that gene prediction and annotation have been performed in advance. If the Reader is interested in processing fragmented genomes, the already published pipeline PanDelos-Frags [26] is available for that purpose and will soon be modified by replacing the original PanDelos approach with the parallel version presented in this work.

#### PanDelos methodology and limitations.

PanDelos is a parameter-free, alignment-free algorithm for identifying gene homology in pangenomic analysis. It leverages information theory and network analysis to achieve this. PanDelos quantifies homology using k-mer multiplicity, with the k-length determined in a non-empirical manner [15].

The process of detecting gene homology in PanDelos is organized into five steps, which can be conceptually grouped into two main phases: an initial candidate selection phase based on k-mer dictionaries, followed by a refinement phase based on network analysis. Regarding the first phase, an optimal k-mer length is initially determined according to the characteristics of the input genome collection. Then, candidate homologous gene pairs are identified by applying coverage-based filters on their k-mer dictionaries. Subsequently, bidirectional best hits (BBHs) are identified by computing the generalized Jaccard similarity for all candidate gene pairs. These BBHs are used to construct a gene–gene homology network, which encodes putative orthologous, paralogous, and xenologous relationships among genes. Finally, during the second phase, gene families are extracted from this network by applying a community detection algorithm to identify densely connected gene clusters [15].

The core algorithm of PanDelos’ pipeline relies on the enhanced suffix array [30] to compute sequence similarity. This structure, combining a suffix array [31] with the longest common prefix (LCP) array, enables efficient enumeration of shared substrings and k-mers across genes.

Despite its substantial impact on computational efficiency, this approach has a relevant limitation in terms of memory usage: the construction and storage of the enhanced suffix array over the concatenated gene sequences is memory-intensive and constitutes one of the most resource-demanding components of the pipeline. This memory requirement arises from the need to maintain multiple auxiliary indexing structures in memory, including the suffix array and the LCP array, whose size scales linearly with the total length of the input sequences and can become prohibitive for large genome collections or pangenome-scale analyses.

#### PanDelos-plus pipeline overview.

**Fig 1:**
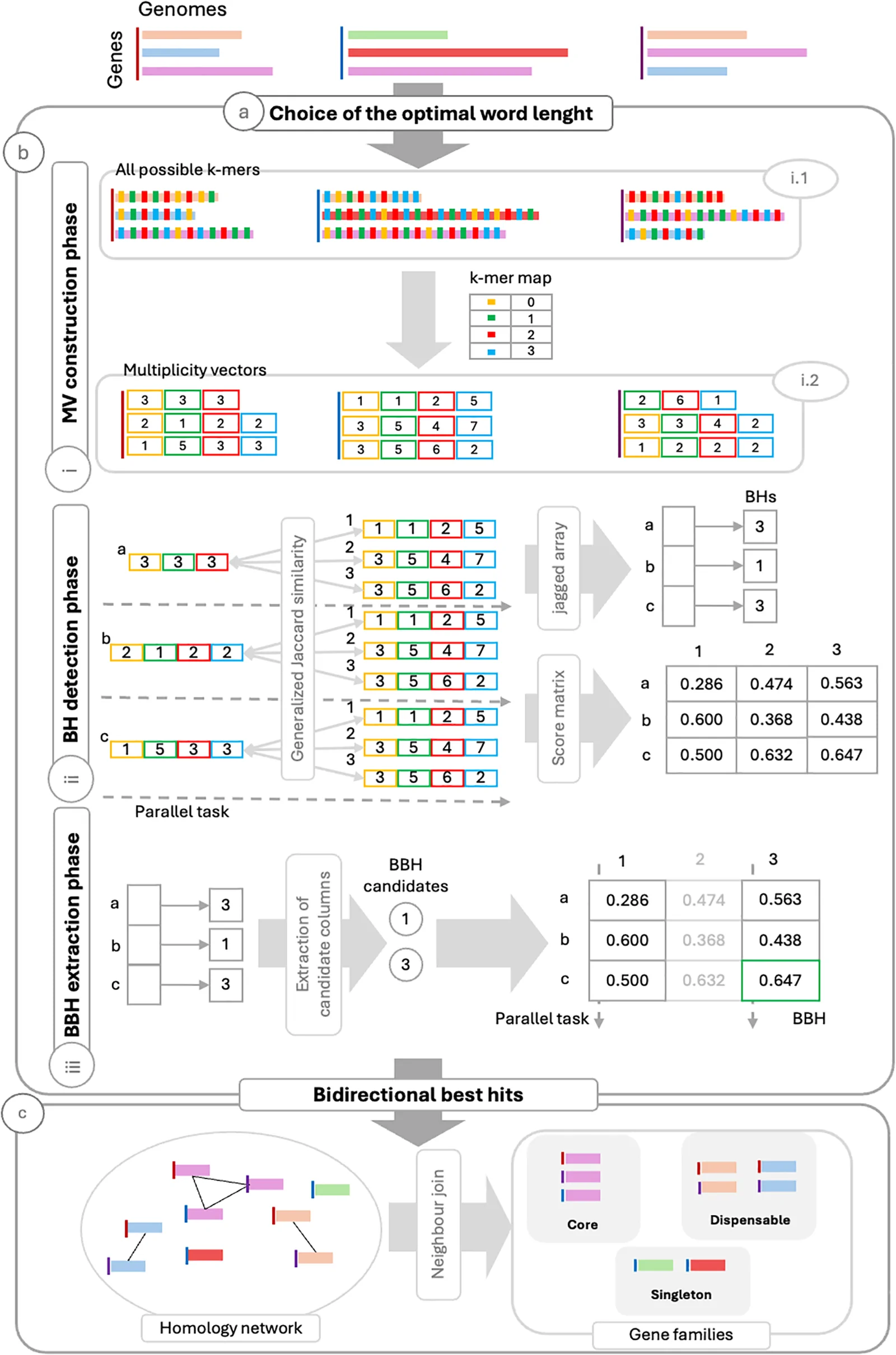
PanDelos-plus pipeline overview. PanDelos-plus pipeline (revisiting PanDelos) in three stages: (a) automatic selection of the optimal k-mer length; (b) all-against-all gene comparison to generate bidirectional best hits (BBH), with three subphases—serial construction of multiplicity vectors (k-mer extraction and grouping), parallel best hit detection via generalized Jaccard similarity (stored in a jagged array), and parallel BBH extraction; (c) final clustering into gene families based on the BBH.

PanDelos-plus pipeline (Fig 1) is a redesign of PanDelos, so some steps of the pipeline are inherited. The pipeline is composed of three macro phases. The first phase (a) is used to determine the optimal k-mer length based on the input dataset. The second phase (b) focuses on an all-against-all comparison of all input genomes, to generate bidirectional best hit. The third phase (c) is used to extract gene families from the bidirectional best hit, via a network-based refining procedure according to the original methodology [15].

The most computationally demanding phase is the second one (b), which consists of three sub-phases: (i) Multiplicity Vector construction, (ii) Best hit detection, and (iii) Bidirectional best hit extraction. The first sub-phase (i) is serial and processes all analyzed genomes, whereas the second and third sub-phases (ii and iii) are parallel and involve all-against-all comparisons between genomes.

The multiplicity vector construction phase (i) is serial. During this phase, all k-mer occurrences are extracted from each gene and mapped to a unique integer identifier (i.1). Occurrences sharing the same identifier are then aggregated to compute the multiplicity of each k-mer within the gene (i.2). The resulting multiplicity vector is a data structure associated with each gene and consists of an ordered list of pairs $\left(f(\alpha ),{mult}_{g}(\alpha )\right)$, where f(α) denotes the integer identifier of k-mer α and ${mult}_{g}(\alpha )$ its multiplicity in gene *g*. The vector is sorted in ascending order with respect to the k-mer identifier f(α).

The best hit detection phase (ii) is parallelized using data decomposition. In this phase, the algorithm computes an all-against-all comparison of genes between a pair of genomes. For each gene in the first genome, the similarity to every gene in the second genome is computed using the generalized Jaccard similarity. These similarity scores are conceptually organized as a gene-by-gene matrix, where rows correspond to genes in the first genome and columns correspond to genes in the second genome. Best Hits for each gene are identified by selecting the genes in the opposite genome with the highest similarity along each row. The resulting Best Hits are stored in a jagged array data structure for efficiency.

The bidirectional best hit extraction phase (iii) is also parallelized. In this phase, the previously computed similarity matrix and jagged array are used to identify pairs of genes that are reciprocal best hits (i.e., each gene is the Best Hit of the other), forming the set of bidirectional best hits used for downstream clustering.

#### Multiplicity vector construction phase.

The first phase of the algorithm is the construction of the multiplicity vectors (MVs). The MV is a data structure that stores, for each gene, all distinct k-mers along with their associated multiplicities.

Consider a function $f(\alpha ):\Gamma^{k}\to \mathbb{N}$ that assigns a unique, consecutive integer identifier to each distinct k-mer found in the dataset. This mapping is constructed incrementally based on the order of appearance: the first unique k-mer encountered is assigned 0, the second is assigned 1, and so on. This function relies on a shared hash map to store the association between k-mers and their integer identifiers. While the MVs for different genes are independent, their construction relies on this shared map, which is dynamically populated as new k-mers are encountered. This design choice avoids race conditions and the overhead of synchronization mechanisms (e.g., locks) that would be required for concurrent write access to the map. Once the MVs are built during this serial phase, the hash map is discarded, as it is no longer required for the subsequent parallel comparison phases.

The MV contains a set of pairs $\left(f(\alpha ),\;mult_{g_{i,j}}(\alpha )\right)$, where f(α) is the unique identifier of the k-mer α and $mult_{g_{i,j}}(\alpha )$ is the multiplicity of the k-mer α in the gene $g_{i,j}$. The MV is defined as:


$$\begin{array}{c} MV(g_{i,j})=\{(f(\alpha ),\;mult_{g_{i,j}}(\alpha )):\alpha \in D_{k}(g_{i,j})\} \end{array}$$ (11)

The MV is sorted in ascending order according to the f(α) value, so that elements with lower f(α) appear first and elements with higher f(α) appear last.

The procedure outlined in Algorithm 1 is iteratively executed for every gene *g* in each genome G∈𝔾 to construct its corresponding MV. It is important to note that once the MVs are constructed, all subsequent computations rely solely on integer operations on these data structures. This approach effectively eliminates the need for computationally expensive string comparisons during the similarity calculation phases.

**Algorithm 1 Multiplicity Vector construction phase for a single gene**

**Require:** Gene $g_{i,j}$, k-mer length *k*, alphabet Γ

**Ensure:** Multiplicity Vector $MV(g_{i,j})$

1: Initialize an empty ordered map *M* ▷ key: k-mer id, value: multiplicity

2: **for** p←1 to $|g_{i,j}|-k+1$ **do** ▷ Slide a window of length *k*

3:   $\alpha \leftarrow g_{i,j}[p...p+k-1]$ ▷ Extract substring of length *k*

4:   id←f(α) ▷ Compute unique identifier for α

5:   **if** id∈M **then**

6:     M[id]←M[id]+1 ▷ Increment multiplicity

7:   **else**

8:     Insert (*id*, 1) into *M* ▷ First occurrence of this k-mer

9:   **end if**

10: **end for**

11: $MV(g_{i,j})\leftarrow$ convert *M* to a vector of pairs (*id*, *multiplicity*) in ascending order of *id*

12: **return** $MV(g_{i,j})$

#### Best hit detection phase.

The second phase of the algorithm is the Best Hit detection. The best hit detection is a parallelized process that computes the similarity between the genes of two genomes $G_{i}$ and $G_{j}$ and detects Best Hits.

All possible comparisons can be represented as a matrix, where the rows represent the genes of genome $G_{i}$ and the columns represent the genes of the genome $G_{j}$. We denote by $S_{i,j}$ the similarity matrix between genomes $G_{i}$ and $G_{j}$, where the entry $S_{i,j}[l,z]$ represents the similarity between the *l*-th gene of $G_{i}$ and the *z*-th gene of $G_{j}$.

The matrix is defined as:


$$\begin{array}{c} S_{i,j}=[\begin{array}{cccc} s_{1,1} & s_{1,2} & ... & s_{1,m}\\ s_{2,1} & s_{2,2} & ... & s_{2,m}\\ \vdots & \vdots & \ddots & \vdots \\ s_{n,1} & s_{n,2} & ... & s_{n,m} \end{array}] \end{array}$$ (12)

Where $m=|G_{j}|$, $n=|G_{i}|$, and $s_{l,z}$ is the similarity value between the gene $g_{i,l}$ and the gene $g_{j,z}$, with 1≤l≤n∧1≤z≤m.

In addition to this matrix, a jagged array is defined to keep tracks of the Best Hit on each row. The jagged array is defined as:


$$\begin{array}{c} A_{i,j}=[\begin{array}{c} Ar_{1}\\ Ar_{2}\\ \vdots \\ Ar_{n} \end{array}] \end{array}$$ (13)

Where $Ar_{i}$ is a vector that contains the column identifiers of Best Hits of the *i*-th row of the matrix $S_{i,j}$. So the $Ar_{i}$ is defined as:


$$\begin{array}{c} Ar_{i}=[\begin{array}{cccc} a_{i,1} & a_{i,2} & ... & a_{i,o} \end{array}] \end{array}$$ (14)

where $a_{i,j}$ represents the index of the column of the *j*-th best hit of the *i*-th row.

Using this representation, each row of the matrix $S_{i,j}$ and its corresponding row of the jagged array $A_{i,j}$ form a unit of work (UoW). All units of work are independent of each other, allowing the computation to be parallelized. Each UoW includes computing the similarity between one gene from $G_{i}$ and all genes of $G_{j}$, along with tracking the Best Hits in the corresponding jagged array row. The total number of units of work is equal to the number of genes in the first genome $G_{i}$. The procedure is detailed in Algorithm 2.

**Algorithm 2 Best Hit detection phase**

**Require:** Genomes $G_{i}$, $G_{j}$, length based threshold θ, coverage threshold τ and word length *k*

**Ensure:** Similarity matrix $S_{i,j}$ and jagged array $A_{i,j}$

1: Initialize $S_{i,j}$ as an n×m matrix ▷ $n=|G_{i}|$, $m=|G_{j}|$

2: Initialize $A_{i,j}$ as an empty jagged array of size *n*

3: **parallel for** $g_{i,l}\in G_{i}$ **do** ▷ Each row is an independent unit of work

4:   **for all** $g_{j,z}\in G_{j}$ **do**

5:     $s_{l,z}\leftarrow similarity(g_{i,l},g_{j,z},\theta ,\tau )$

6:     Store $s_{l,z}$ in $S_{i,j}[l,z]$

7:   **end for**

8:   $Ar_{l}\leftarrow$ indices of maximum values in the row $S_{i,j}[l,...]$ ▷ Best Hits of $g_{i,l}$

9:   Store $Ar_{l}$ in $A_{i,j}[l]$

10: **end parallel for**

11: **return** $S_{i,j},A_{i,j}$

The *similarity* function invoked by Algorithm 2 return 0 if $|g_{i,l}|<|g_{j,z}|\cdot \theta \vee |g_{j,z}|<|g_{i,l}|\cdot \theta$ or $p_{k}(g_{i,l}\to g_{j,z})<\tau \vee p_{k}(g_{j,z}\to g_{i,l})<\tau$, otherwise it returns $J_{k}(g_{i,l},g_{j,z})$ computed by exploiting the multiplicity vectors of the two genes that have been previously computed and that are available as global variables.

#### Bidirectional best hit extraction phase.

The third phase of the algorithm is the bidirectional best hit extraction. This phase is parallelized and extracts bidirectional best hits using the similarity matrix $S_{i,j}$ and the jagged array $A_{i,j}$ computed in the previous phase.

A bidirectional best hit between a gene in genome $G_{i}$ and a gene in genome $G_{j}$ requires that each gene is the best hit of the other. Concretely, a gene corresponding to row *l* in $S_{i,j}$ and a gene corresponding to column *z* in $S_{i,j}$ form a BBH if the gene at column *z* is among the best hits of row *l*, and reciproca*l*ly the gene at row *l* is among the best hits of column *z* when considering the reverse comparison.

Therefore, only a subset of the matrix $S_{i,j}$ needs to be considered in this phase, namely the columns that contain at least one best hit of any row. Exploiting the jagged array $A_{i,j}$, it is possible to efficiently identify the relevant columns that must be processed during BBH extraction.

As in the best hit detection phase, the Bidirectional best hit extraction phase is parallelized by decomposing the computation into independent units of work. In this case, the matrix $S_{i,j}$ is partitioned by columns, and each relevant column constitutes an independent unit of work. The number of such units corresponds to the number of genes in the second genome $G_{j}$ that appear at least once as a best hit in $A_{i,j}$.

Relying on the independence of these columns and on the matrix representation of the computed Jaccard similarities, each column is processed independently. Each unit of work includes scanning one column of $S_{i,j}$ and cross-checking the corresponding Best Hit information in $A_{i,j}$ to identify reciprocal Best Hits. The bidirectional best hit extraction procedure is summarized in Algorithm 3.

**Algorithm 3 Bidirectional best hit extraction phase**

**Require:** Similarity matrix $S_{i,j}$, jagged array $A_{i,j}$, genomes $G_{i}$, $G_{j}$

**Ensure:** Set of Bidirectional Best Hits *BBH*

1: BBH←∅

2: Identify relevant columns of $S_{i,j}$ from $A_{i,j}$ ▷ Columns that contain at least

3: **parallel for** each relevant column *z* **do** ▷ Each column is an independent UoW

4:   **for all** rows *l* such that $z\in A_{i,j}[l]$ **do** ▷ Candidate Best Hits

5:     **if** $S_{i,j}[l,z]=\max_{r}S_{i,j}[r,z]$ **then** ▷ $g_{i,l}$ is a Best Hit of $g_{j,z}$

6:       **if** $l\in A_{j,i}[z]$ **then** ▷ Cross-check: reciprocal Best Hit

7:         Add pair $\left(g_{i,l},g_{j,z}\right)$ to *BBH*

8:       **end if**

9:     **end if**

10:   **end for**

11: **end parallel for**

12: **return** *BBH*

### Dataset construction

Two sets of experiments have been conducted to evaluate the computational performance of PanDelos-plus. The first set, referred to as the real datasets, is composed of real bacterial genomes, and is used to compare the performance of PanDelos-plus with the original PanDelos algorithm, and to measure scalability in runtime and memory usage as the thread count grows. The second set, referred to as the synthetic datasets, is composed of synthetic bacterial genomes, and is used to measure scalability on large genome collections generated via PANPROVA [27].

PANPROVA is a computational tool designed to simulate the evolution of prokaryotic pangenomes by evolving the complete genomic sequence of an ancestral isolate to generate synthetic datasets. It operates by taking a full genome and its gene annotations, then simulating evolutionary events (such as mutations, gene loss, gene duplication, and horizontal gene transfer) under user-defined parameters, resulting in a phylogenomic tree populated with fully assembled, evolutionarily related synthetic genomes. This tool is used primarily to create realistic synthetic bacterial genomic datasets for benchmarking and evaluating pangenomic analysis tools, especially in scenarios where complete and controlled reference data are needed.

The real dataset (see Table 1) includes four real-world bacterial groups taken from the original PanDelos paper. These groups (listed in S1 File) comprise complete genomes of *Escherichia coli*, *Salmonella enterica*, and *Xanthomonas campestris*, along with a collection of different *Mycoplasma* species, downloaded from the NCBI RefSeq database, which provides standardized and consistently annotated reference genomes.

**Table 1:** Summary of real bacterial datasets used for computational assessment.

Dataset	Number of genomes	Total number of genes	Max. number of genes	Min. number of genes	Mean of number of genes	Standard deviation of number of genes
*Escherichia coli*	10	46724	5195	4209	4672.40	340.41
*Salmonella enterica*	7	29898	4325	4212	4271.14	35.71
*Xanthomonas campestris*	14	55290	4300	3023	3949.29	339.58
*Mycoplasma*	64	48117	1495	479	751.83	181.41

Each dataset consists of complete genomes downloaded in REFSEQ format from NCBI, originally used in the PanDelos study. The table reports, for each species group, the total number of genomes, the total gene count, and descriptive statistics of gene content per genome (maximum, minimum, mean, and standard deviation).

The synthetic dataset (see Table 2) includes a collection of synthetic bacterial pangenome datasets generated using PANPROVA starting from an ancestral *Escherichia coli* genome. Genome evolution was modelled by allowing horizontal gene transfer events from a predefined gene pool composed of nine external genomes (listed in S2 File).

**Table 2:** Overview of synthetic datasets used for computational assessment.

Collection	Number of genomes	Total number of genes	Max. number of genes	Min. number of genes	Mean of number of genes	Standard deviation of number of genes
Collection 1	50	269882	5559	5202	5397.64	80.27
Collection 2	100	540231	5608	5202	5402.31	75.79
Collection 3	150	812378	5650	5202	5415.85	83.70
Collection 4	200	1084178	5651	5202	5420.89	86.53
Collection 5	250	1356037	5658	5202	5424.15	87.42
Collection 6	300	1628181	5687	5202	5427.27	89.85
Collection 7	350	1901281	5707	5202	5432.23	92.27
Collection 8	400	2173975	5707	5202	5434.94	91.45
Collection 9	450	2446401	5758	5202	5436.45	92.77
Collection 10	500	2719951	5758	5202	5439.90	94.68
Collection 11	550	2994448	5762	5202	5444.45	96.73
Collection 12	600	3268551	5790	5202	5447.59	98.37

Each collection is generated with PANPROVA from an ancestral Escherichia coli genome. Collections are nested: each is obtained by adding 50 genomes to the previous one, so the N-genome collection is a strict subset of the (N + 50)-genome collection. The minimum gene count is identical (5202) across all collections because the smallest genome belongs to the initial 50 and is therefore present in every collection; maximum and mean values increase as larger genomes are added.

To ensure consistency across experiments, we generated a comprehensive master dataset of synthetic genomes using PANPROVA. From this pool, we derived 12 experimental collections of increasing size, ranging from 50 to 600 genomes. These collections were built incrementally: we started with a core set of 50 genomes and sequentially added 50 distinct genomes from the master pool to create each subsequent collection. Consequently, the genomes in the *N*-sized collection are a strict subset of those in the (*N* + 50)-sized collection (e.g., the 100-genome dataset contains the exact same genomes as the 50-genome dataset, plus 50 additional ones). This design ensures that all collections originate from the same simulated evolutionary history, allowing scalability to be evaluated solely as a function of dataset size, without confounding effects arising from independently generated genome sets.

Lastly, we generated two additional synthetic datasets for comparing the proposed approach with the current state of the art. The simulated genomes were derived from an ancestral Escherichia coli genome, which was evolved using an HGT pool consisting of 9 genomes (same as Collections 1−12 and detailed in S2 File). An overview of the properties of such datasets is given in Table 3.

**Table 3:** Overview of datasets used in quality tests.

Dataset	Number of genomes	Total number of genes	Max. number of genes	Min. number of genes	Mean of number of genes	Standard deviation of number of genes
Collection A	50	271477	5705	5202	5429.54	105.91
Collection B	100	547922	5753	5202	5479.22	112.06

Both collections were generated independently using PANPROVA and by utilising the same ancestral Escherichia coli genome and HGT pool.

## Results

We evaluate the computational performance of the proposed approach, also compared to the original one, over the real and synthetic datasets by measuring wall-clock time, CPU time, peak memory usage, and speedup efficiency as the thread count varied. Furthermore, we evaluated the enabling of the proposed approach to retrieve a better refined pangenomic information by analysing the change in family diffusivity when an increased number of genomes is taken into account. Lastly, we assessed the outperformance of the PanDelos and PanDelos-plus methodologies relative to the most recent state-of-the-art approaches for retrieving the correct homology using statistical measures commonly used in machine learning.

All tests were performed through the University of Parma’s high-performance computing (HPC) facility. The compute node was equipped with dual AMD EPYC 7282 CPUs, providing a total of 32 physical cores (corresponding to 32 logical cores, as SMT was disabled), and 256 GB of RAM. Detailed hardware specifications, including cache sizes and architecture configuration, are provided in S3 File.

### Computational performance on real datasets

In all the four real datasets, PanDelos-plus, thanks to its lightweight data structure and parallelization of the most demanding phases, outperforms the original PanDelos in both execution time and memory consumption (Table 4). Specifically, it demonstrates performance advantages, in execution time and memory consumption over PanDelos across all real datasets. For instance, on the *Escherichia coli* dataset, PanDelos required 619 s and 20.2 GB of RAM, whereas PanDelos-plus completed the same analysis in just 46 s using only 0.7 GB, achieving a 13.5x speedup and a 96.5% reduction in memory. In the *Mycoplasma* test, the execution time fell from 563 s to 65 s and maximum RAM from 2.2 GB to 0.7 GB, corresponding to an acceleration of 8.7x and 68.2% less memory usage. For *Salmonella enterica*, PanDelos-plus reduced runtime from 216 s to 16 s and memory from 12.8 GB to 0.5 GB again a 13.5x speedup alongside a 96.1% RAM saving. Finally, in *Xanthomonas campestris*, the enhanced implementation ran in 73 s instead of 1015 s and used 0.7 GB rather than 15.7 GB, translating into a 13.9x faster execution with a 95.5% reduction in memory footprint. These substantial gains in speed and memory efficiency establish PanDelos-plus as a more powerful tool than its predecessor.

**Table 4:** Comparison of PanDelos and PanDelos-plus performance metrics across real bacterial datasets.

Dataset	PanDelos (1 thread)		PanDelos-plus (32 threads)		Runtime speedup	Memory reduction (%)
	Time (s)	Memory (GB)	Time (s)	Memory (GB)	Speedup	Reduction
*Escherichia coli*	619	20.2	46	0.7	13.5	96.5
*Salmonella enterica*	216	12.8	16	0.5	13.5	96.1
*Xanthomonas campestris*	1015	15.7	73	0.7	13.9	95.5
*Mycoplasma*	563	2.2	65	0.7	8.7	68.2

Each dataset was analysed using PanDelos with a single thread and PanDelos-plus with 32 threads. Metrics include execution time, peak memory usage, speedup factor, and percentage reduction in memory consumption.

In all four datasets, PanDelos-plus demonstrates high efficiency in resource scaling; the impact of increasing the number of processing threads leads to a drastic reduction in execution time while maintaining a nearly constant memory footprint (Table 5). This analysis, detailed below, examines the impact of an increasing number of processing threads on execution time and memory usage for a fixed input dataset across the same experimental collections.

**Table 5:** Comparison of PanDelos-plus 1 thread and PanDelos-plus 32 threads performance metrics across real bacterial datasets.

Dataset	1 thread		32 threads			
	Time (s)	Memory (GB)	Time (s)	Total CPU time (s)	Memory (GB)	Total CPU time (s) / 32
*Escherichia coli*	888.30	0.61	46.30	974.58	0.72	30.46
*Salmonella enterica*	262.27	0.37	16.02	261.87	0.49	8.18
*Xanthomonas campestris*	1660.27	0.71	73.84	1634.92	0.71	51.09
*Mycoplasma*	1229.57	0.65	65.23	1091.79	0.65	34.12

Comparison of PanDelos-plus execution metrics. Memory usage, while showing minor variations across different thread counts, remains overall stable and will be further analyzed in subsequent sections. The ratio *Total CPU / 32* is consistently lower than the actual wall-clock time (*Time*) as the program includes serial code sections where parallelization would not have provided benefits or would not be possible due to synchronization requirements.

To further investigate how effectively PanDelos-plus exploits computational resources when parallelized, we conducted a strong scaling analysis. For all the real datasets, an almost ideal speed up is observed, as doubling the number of threads approximately halves the execution time. Memory usage remains nearly constant as the number of threads increases. This indicates that increasing parallelism does not introduce additional memory overhead, independent of the parallel speedup achieved (discussed below).

**Fig 2:**
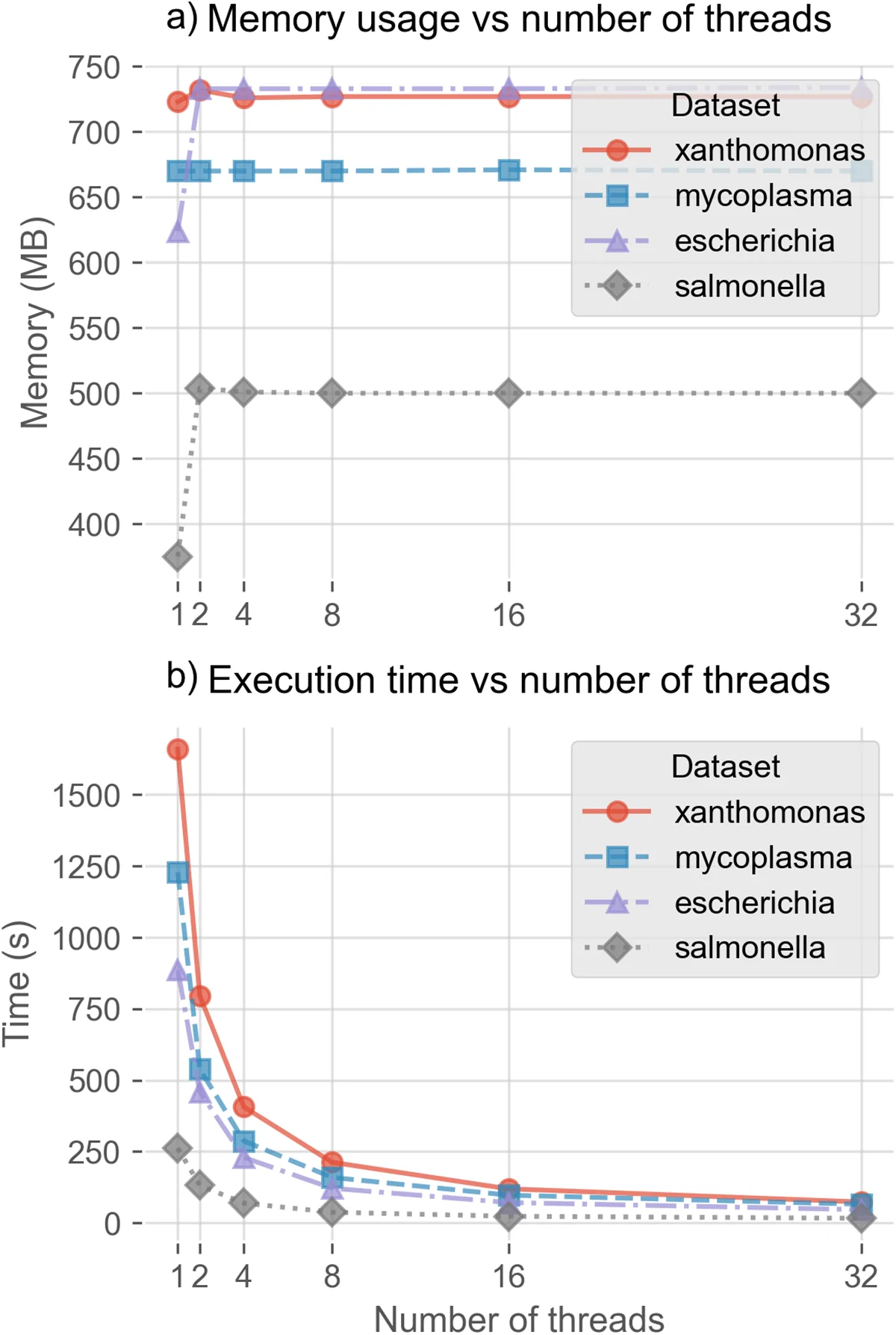
Strong scaling of PanDelos-plus on real datasets. **(a)**: Peak memory usage (MB) remains overall constant as the number of threads increases, indicating minimal overhead from parallelization. **(b)**: Execution time (s) decreases almost inversely with the number of threads, confirming efficient parallel scalability across all datasets.

The plot in Fig 2a shows that memory consumption (in MB), barring a few minor variations, remains essentially constant for each dataset as the number of threads increases. This indicates that increasing parallelism does not increase the memory footprint for a given problem size. The plot in Fig 2b shows a reduction in execution time (in seconds) as the number of threads increases for all datasets.

### Computational performance on synthetic datasets

To assess the scalability of PanDelos-plus on increasingly large collections, we conducted two series of tests on the synthetic dataset generated using PANPROVA.

**Fig 3:**
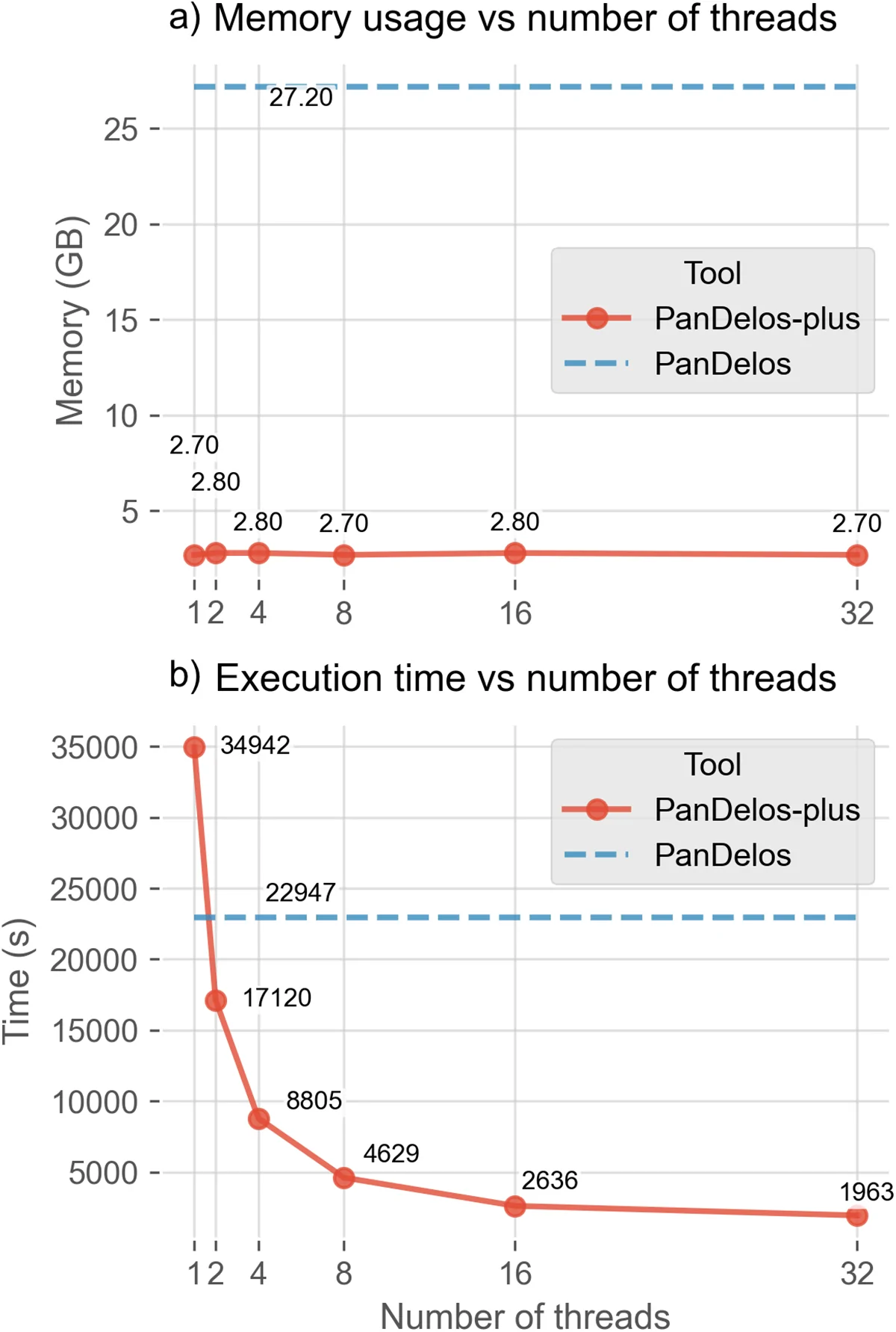
Scaling behavior of PanDelos-plus on a synthetic dataset of 50 genomes. **(a)** Memory usage (GB) remains essentially constant as the number of threads increases. PanDelos-plus requires approximately 2.8 GB, whereas PanDelos requires about 27.2 GB. **(b)** Execution time (s) decreases approximately inversely with thread count, from about 34942 s (9.6 h) with 1 thread to approximately 1963 s (0.5 h) with 32 threads. This corresponds to approximately 18x speedup relative to single-threaded execution, indicating strong but sub-ideal parallel scaling.

**Fig 4:**
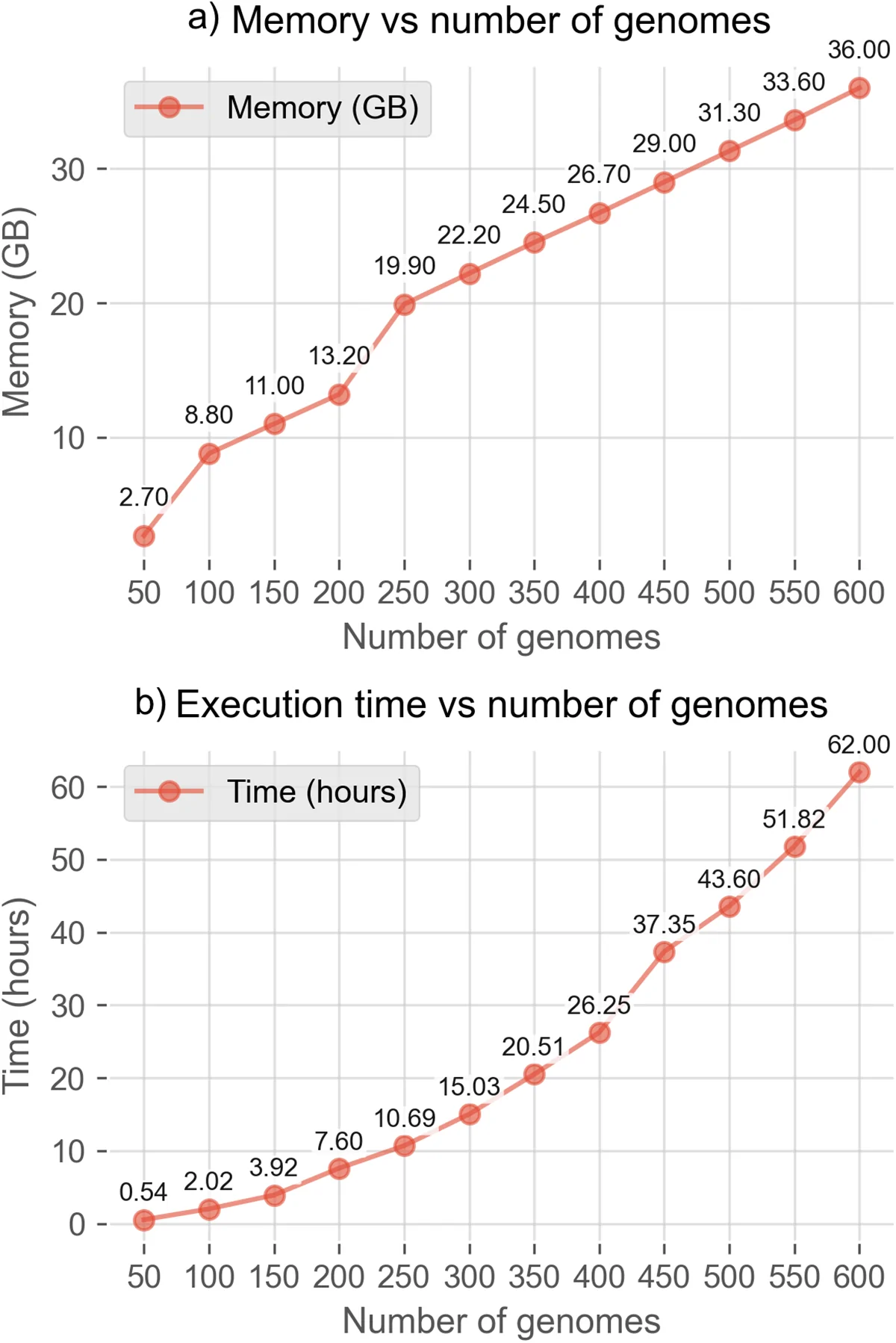
Scalability of PanDelos-plus with increasing collection size (50-600 genomes) at 32 threads. **(a)** Peak memory usage grows approximately linearly with the number of genomes, remaining below 40 GB even for 600 genomes. **(b)** Execution time increases from ≈ 0.5 h (50 genomes) to ≈ 62 h (600 genomes). While the number of genomes grows by a factor of 12, runtime increases by more than two orders of magnitude, exhibiting superlinear scaling and indicating that computational costs grow more rapidly than linearly as collection size increases.

In the first one (Fig 3), the collection size was fixed to 50 genomes (Table 2, Collection 1) and we varied the number of threads from 1 to 32, measuring peak memory usage and execution time. In this instance, PanDelos has been introduced as a baseline for comparison, using a single thread as it does not support parallel execution. In the second part (Fig 4), we used a constant number of threads (namely, 32) and observed how memory usage and execution time grow as the number of genomes increases from 50 to 600 (Table 2, Collections 1–12). PanDelos has not been included as it cannot handle collections of this size.

When analysing the 50 genomes (Table 2, Collection 1), peak memory usage remains essentially constant and the time decreases markedly as the number of threads increases. This indicates that increasing parallelism does not substantially affect the RAM required for a fixed problem size, while it does improve execution time.

The plot in Fig 3a shows that memory consumption (in GB) remains essentially constant as the number of threads increases, with only minor variations. Here PanDelos-plus’s memory usage is substantially lower than PanDelos’s, respectively, approximately 2.8 GB vs approximately 27.2 GB.

The plot in Fig 3b shows a reduction in execution time (in seconds) as the number of threads increases. Execution times for the 50-genome collection are reported in Table 6.

**Table 6:** Execution time for the analysis of 50 genomes.

Method	Threads	Time (s)	Time (h)
PanDelos	1	22947	6.37
PanDelos-plus	1	34942	9.60
PanDelos-plus	2	17120	4.70
PanDelos-plus	4	8805	2.40
PanDelos-plus	8	4629	1.30
PanDelos-plus	16	2636	0.73
PanDelos-plus	32	1963	0.55

Even with only 2 threads, PanDelos-plus is already faster than PanDelos. At 32 threads, runtime is reduced by approximately 18x relative to the single-threaded execution, compared to the theoretical 32x reduction expected under ideal scaling. This indicates strong but non-ideal parallel performance, with diminishing scaling gains at higher thread counts.

The next test, using a constant number of threads (32) shows that both memory usage and execution time grow as the number of genomes increases from 50 to 600.

The plot in Fig 4a shows that with 32 threads PanDelos-plus’s peak memory consumption (in GB) grows as the number of genomes increases from 50 to 600. For 50 genomes, the peak memory is approximately 2.7 GB. As we add more genomes, memory usage rises almost linearly reaching ≈ 36 GB at 600 genomes. This nearly linear trend indicates that PanDelos-plus allocates memory in direct proportion to the total gene count, remaining within acceptable bounds (under 40 GB) even for a 600-genome pangenome on a 32-core node.

The plot in Fig 4b shows that with 32 threads the runtime increases from approximately 0.5h (1900s) for 50 genomes up to approximately 62h (225000s) for 600 genomes. While the number of genomes increases by a factor of 12, the execution time increases by roughly a factor of 100, with progressively larger increments as dataset size grows. This pattern indicates superlinear scaling, suggesting that computational costs increase more rapidly than linearly for larger datasets, even when parallelism is employed.

In addition to the scalability benchmarks, we conducted a qualitative evaluation of gene family prediction accuracy on the synthetic datasets. Using the gene families established by PANPROVA as ground truth, we compared the predictions of PanDelos-plus against PPanGGOLiN [19] and PanTools v3 [22] by computing Precision, Recall, and F1-score.

### Diffusivity change on dataset size increase

We evaluated how gene family diffusivity changes when the number of genomes included in a pangenomic reconstruction increases. The objective is to assess the biological consequences of enabling large-scale genome integration. To this end, we compare pangenomes reconstructed from two configurations for each species group of the real datasets: *Reduced set*, that is, approximately one-third of the available genomes and *Complete set*, that is, the full genome collection. By contrasting these two conditions, we assess whether scaling the genome number affects the inferred gene family landscape and the resulting diffusivity profiles.

Table 7 summarises the global statistics for the Reduced and Complete sets.

**Table 7:** Dataset overview: Total counts of genomes, genes, and gene families for Reduced and Complete sets.

Dataset	Reduced			Complete		
	Genomes	Genes	Families	Genomes	Genes	Families
*Escherichia coli*	3	14376	5930	10	46724	7203
*Salmonella enterica*	2	8477	4251	7	29898	4441
*Xanthomonas campestris*	4	15457	5817	14	55290	8334
*Mycoplasma*	21	15735	8743	64	48117	13100

The table shows the total number of genomes, genes, and gene families identified in each set for four bacterial species groups, respectively, in the reduced and complete set.

As expected, an increase in the number of genomes leads to a substantial increase in total gene counts across all species groups. The number of gene families also increases, albeit sublinearly with respect to the expansion in gene count. This reflects two concurrent processes: redundancy accumulation, in which newly added genomes contribute genes that cluster into existing families; and novel family discovery, in which previously unobserved gene variants form new families. These two processes jointly determine how families are distributed across diffusivity classes. Separately, because diffusivity is defined as the number of genomes in which a family occurs, enlarging the collection necessarily changes the maximum attainable diffusivity, the granularity at which intermediate-frequency dispensable families can be resolved, and the boundaries between core, dispensable, and singleton categories. Redistribution across these categories therefore follows from how the categories are defined relative to the sampled collection, rather than from instability in the family assignments themselves, which remain largely additive. Among the datasets analyzed, *Mycoplasma* shows the largest genome expansion (21–64 genomes) and the largest increase in gene family number, consistent with its broader phylogenetic diversity. This makes it a particularly informative case for assessing the impact of expanded sampling on diffusivity distributions.

Table 8 reports the macro-level correspondence between the Reduced and Complete sets in order to quantify how gene family assignments change when additional genomes are integrated. The majority of gene families identified in the Reduced set are recovered in the Complete set, thus indicating strong structural consistency of family assignments under genome scaling. *Salmonella enterica* exhibits complete conservation, with all 4,251 Reduced families preserved in the Complete reconstruction and no collapsed families. In contrast, *Mycoplasma* and *Xanthomonas campestris* introduce a substantial number of new families (4,641 and 2,573, respectively), reflecting the increased diversity captured by broader genome sampling. Collapsed families remain rare across all datasets, demonstrating that scaling does not destabilize previously inferred families. Instead, the primary structural effect of adding genomes is the introduction of novel families and the expansion of existing ones.

**Table 8:** Family correspondences between the Reduced and Complete sets.

Dataset	Number of families		Correspondence		
	Reduced	Complete	Shared	Collapsed	New
*Escherichia coli*	5930	7203	5814	116	1389
*Salmonella enterica*	4251	4462	4251	0	211
*Xanthomonas campestris*	5817	8361	5788	29	2573
*Mycoplasma*	8743	13268	8627	116	4641

Families are classified as: Shared (present in both sets with ≥80% gene overlap), Collapsed (present in the Reduced set but not matched in the Complete set), and New (present only in the Complete set). This analysis highlights how gene families evolve when additional genomes are integrated into the pangenome.

Beyond macro-level correspondences, Table 9 provides a finer classification of how the internal composition of shared gene families changes when moving from the Reduced to the Complete set. Across all datasets, the dominant category is *Enlarged*, indicating that most shared families in the Complete set contain all genes from the Reduced set plus additional members contributed by newly included genomes. This confirms that scaling primarily results in membership expansion rather than structural reorganization. Unaltered families represent cases in which no additional genes were incorporated, despite the larger genome pool. These are most frequent in *Mycoplasma*, likely corresponding to lineage-specific or low-diffusivity families that remain confined to a limited subset of genomes. Partially Enlarged families are extremely rare (at most four cases), and collapsed families remain minimal. This demonstrates that gene reassignment or fragmentation upon scaling is negligible. Collectively, these results show that genome expansion induces predominantly additive growth of gene families, with minimal restructuring. Consequently, observed changes in diffusivity are not artifacts of unstable clustering but are consistent with the expectation that observed diffusivity shifts primarily as a function of the change in sample size, rather than reflecting instability in the underlying gene family assignments.

**Table 9:** Detailed classification of gene family match types.

Dataset	Correspondence				
	Shared				
	Unaltered	Enlarged	Partially Enlarged	Collapsed	New
*Escherichia coli*	375	5435	4	116	1389
*Salmonella enterica*	28	4223	0	0	211
*Xanthomonas campestris*	926	4861	1	29	2573
*Mycoplasma*	2857	5769	1	116	4641

Match types are defined as: Unaltered (identical gene content in both sets), Enlarged (Complete family contains all genes from the Reduced set plus additional genes), Partially Enlarged (Complete family gained genes but also lost some from the Reduced set), Collapsed (no corresponding family found in the Complete set), and New (family exists only in the Complete set).

**Fig 5:**
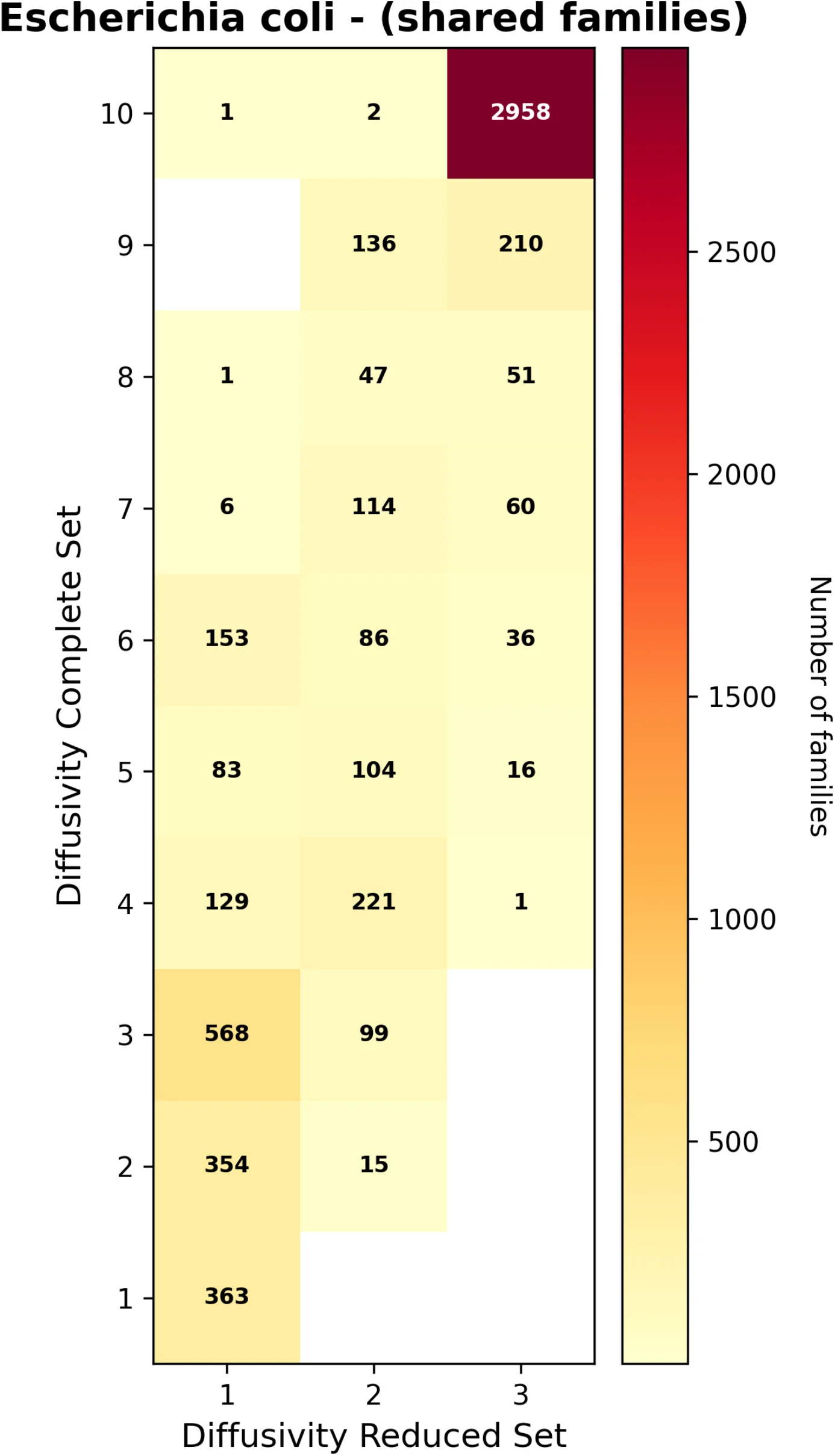
Transition heatmap of shared gene families across diffusivity classes in *Escherichia coli.*

**Fig 6:**
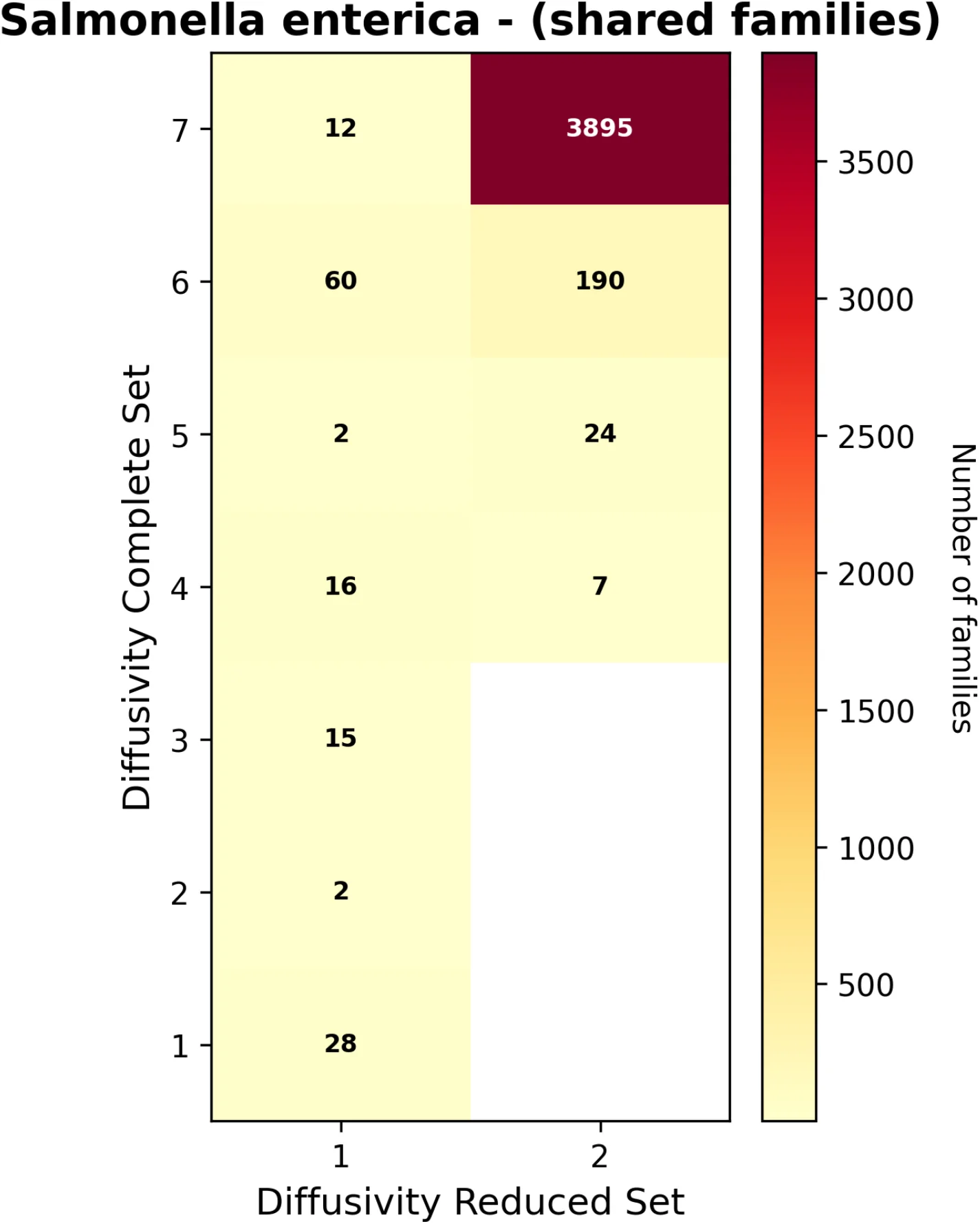
Transition heatmap of shared gene families across diffusivity classes in *Salmonella enterica.*

**Fig 7:**
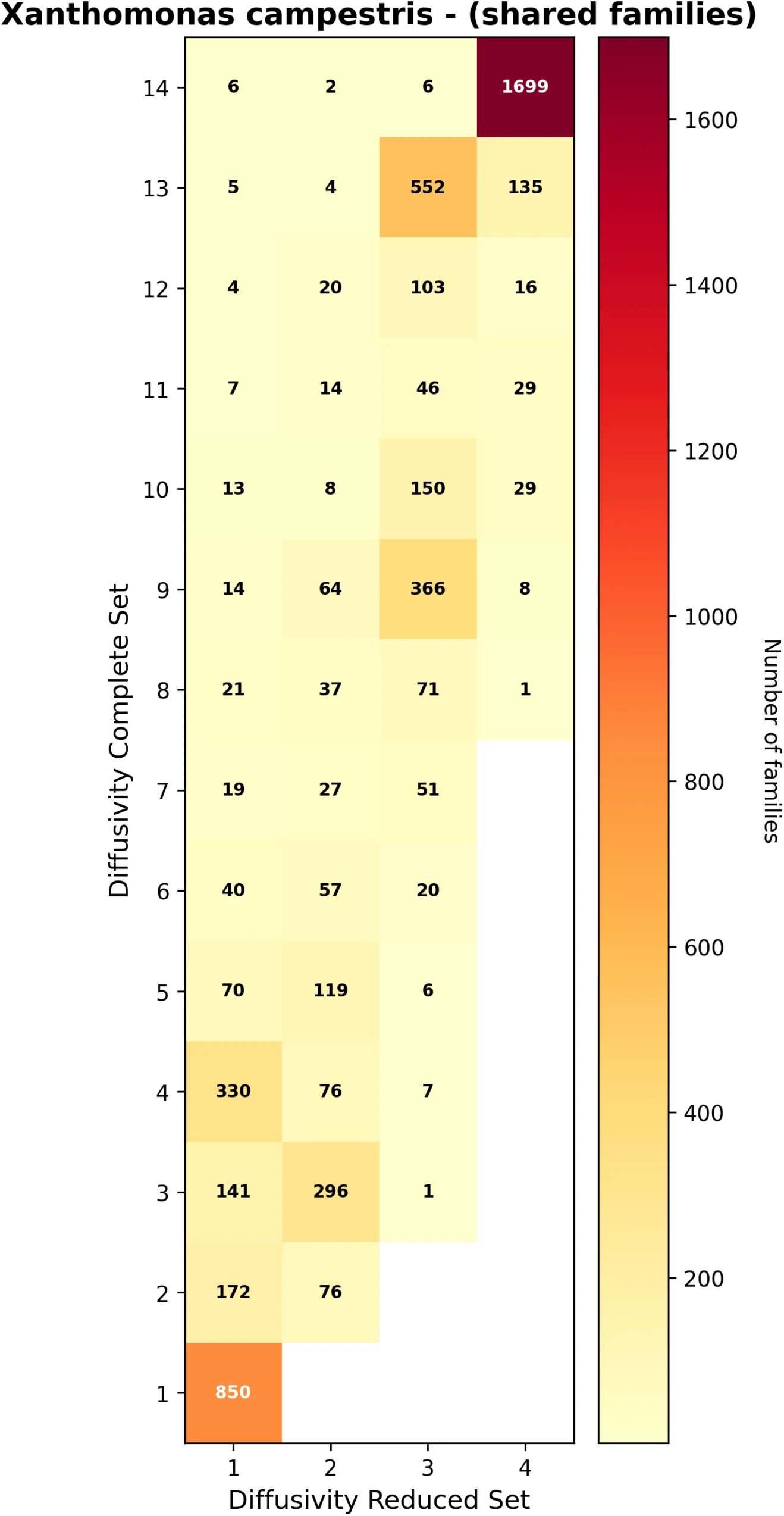
Transition heatmap of shared gene families across diffusivity classes in *Xanthomonas campestris.*

**Fig 8:**
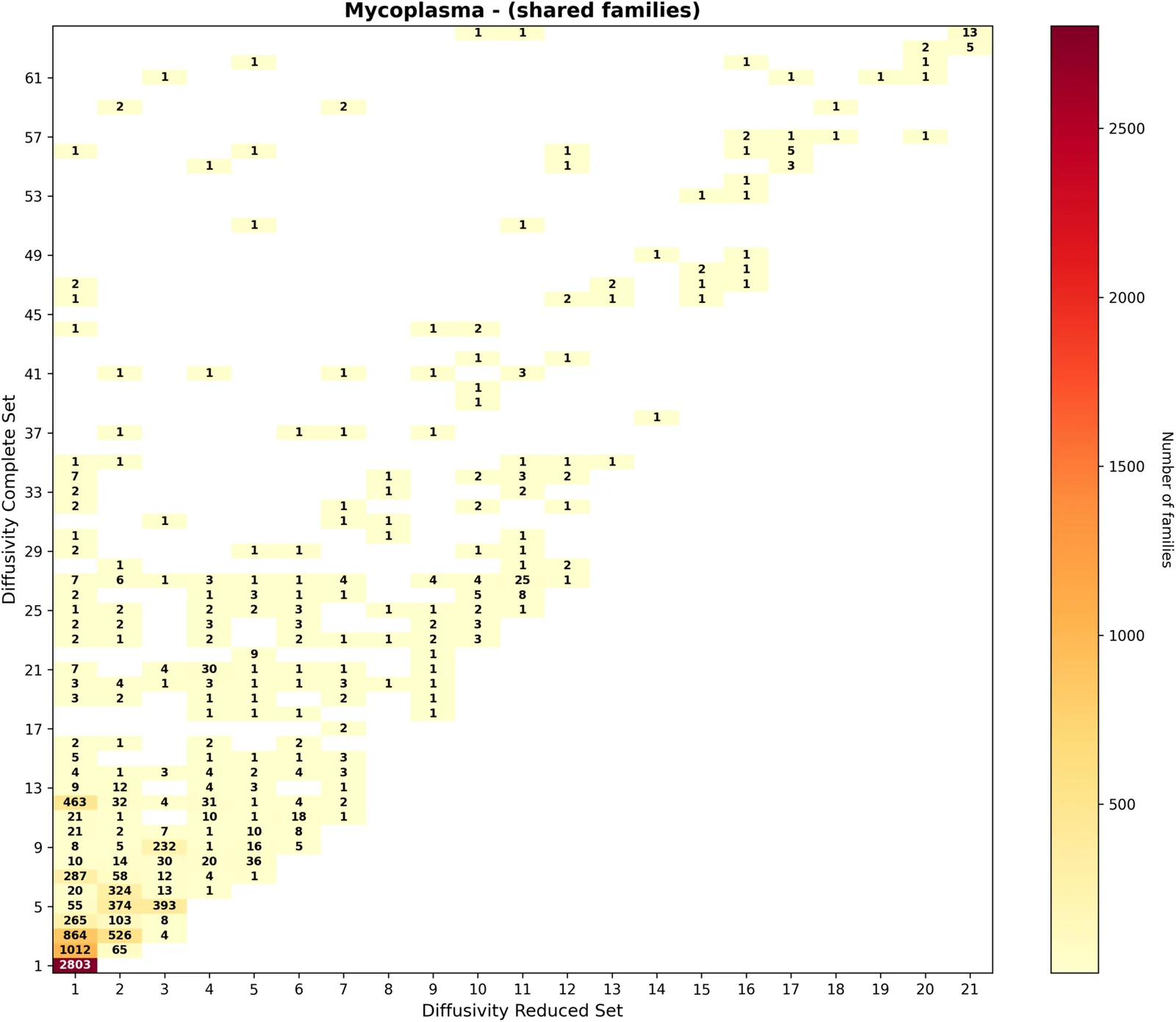
Transition heatmap of shared gene families across diffusivity classes in *Mycoplasma.*

To better show alteration in family diffusivity, we computed heatmaps in which each family is mapped to a cell whose coordinates are given by its diffusivity in the Reduced set and its diffusivity in the Complete set (see Figs 5–Fig 6, Fig 7, Fig 8). This type of analysis allows us to investigate in detail the diffusivity, but also to detect the change in the classification of core, dispensable and singleton families that is essential for biological interpretations in pangenomic studies. Across all datasets, the mass of the heatmaps concentrates along the diagonal, indicating that family assignments remain consistent across sample sizes: the shift toward higher integer diffusivity classes follows from the larger number of sampled genomes rather than from the method reaching different conclusions about the same genes. In *Escherichia coli* (Fig 5) and *Salmonella enterica* (Fig 6), shared families are concentrated along the diagonal, with high-diffusivity families in the Reduced set remaining high-diffusivity in the Complete set. *Xanthomonas campestris* (Fig 7) shows a more heterogeneous pattern, with both upward transitions and broader dispersion across intermediate classes. *Mycoplasma* (Fig 8), analyzed at the genus level, exhibits the strongest concentration in low diffusivity classes, with a dense block at (1,1) reflecting persistent singletons and substantial interspecific diversity. Above this block, families show a diffuse upward gradient toward intermediate diffusivity classes, while high-diffusivity clusters remain limited, consistent with a small strict core genome.

### Comparison with the recent state of the art

During the generation process, PANPROVA models each gene family as a graph where each node represents a gene $g_{i,j}$ and edges represent homology relationships. In particular, given a gene family, all the pairwise edges between the genes belonging to the family are present in the graph. Thus, we evaluated the ability of pangenomic tools to retrieve/predict the correct set of edges. The predictions were compared with the ground truth extracted from the PANPROVA-generated data to determine the number of True Positives (TP), False Positives (FP), False Negatives (FN) and True Negatives (TN). Specifically, True Positives (TP) represent homologous pairs correctly identified; False Positives (FP) are non-homologous pairs incorrectly predicted as homologous; False Negatives (FN) correspond to true homologous pairs missed by the tool; and True Negatives (TN) represent correctly identified non-homologous pairs. Given the all-against-all nature of pairwise comparisons across entire genomes, the number of TNs is expected to be exceptionally high. For this reason, we focus the evaluation on the precision, recall, and F1-score statistical metrics because they do not rely on TN and are not affected by such an imbalance bias. Precision measures accuracy among positive predictions (*TP*/(*TP* + *FP*)), Recall (sensitivity) measures the ability to find all actual positives *TP*/(*TP* + *FN*), while F1-score is their harmonic mean.

Table 10 summarises the total number of gene families and individual homologous gene pairs identified by each algorithm, juxtaposed against the expected ground truth for both synthetic collections. This initial overview highlights the general clustering behaviour, fragmentation, and granularity of the different approaches before assessing their statistical performance. For Collection A (50 genomes), PPanGGOLiN predicted 7,919 families, closest to the ground truth of 7,594, yet largely overestimated the number of homologies (9,237,627 vs. 6,480,361). The two counts are not equally sensitive to the same errors: the number of homologies is dominated by the largest families, since every extra member added to a large family introduces many new pairs, whereas the number of families is dominated by the many small ones. The agreement on the family count is therefore only apparent, and results from two opposite errors that compensate each other: a few erroneous merges of large, nearly core families inflate the homologies, while an excess of small and singleton clusters keeps the family total close to the expected value. PanDelos-plus predicted a moderately higher family count (8,824) while closely approximating the expected number of homologies, indicating that its additional families are small and its large ones correctly delimited. PanTools exhibited pure fragmentation, producing nearly twice as many families (14,334) but fewer homologies than the ground truth. These trends were consistent, and in most cases amplified, in Collection B (100 genomes), where PPanGGOLiN exceeded both the expected number of homologies and the expected number of families (11,532 vs. 10,044), making the coexistence of merging and fragmentation directly visible. PanTools further inflated the family count to 28,363, and PanDelos-plus remained the most balanced across both metrics.

**Table 10:** Number of gene families and gene homologies identified by each tool.

Collection	Expected		PanDelos-plus		PPanGGOLiN		PanTools	
	Families	Hom.	Families	Hom.	Families	Hom.	Families	Hom.
A	7,594	6,480,361	8,824	6,535,521	7,919	9,237,627	14,334	5,593,906
B	10,044	26,170,772	13,834	25,603,506	11,532	36,740,464	28,363	15,241,230

Comparison of the number of gene families and gene homologies predicted by each tool in relation to the number expected counts due to the actual collection composition.

To quantitatively assess the quality of the predicted gene families, we evaluated the pairwise homology relationships using Precision, Recall, and F1-score metrics. Table 11 details the performance achieved by each tool. These metrics provide a comprehensive view of how accurately each algorithm balances the identification of true homologous pairs while minimising the inclusion of incorrect associations. PanDelos-plus achieved the highest F1-score on both Collection A (0.9635) and Collection B (0.9674), maintaining a strong balance between Precision and Recall. Notably, its performance remained stable or even slightly improved as the number of genomes scaled from 50 to 100. PPanGGOLiN achieved high Recall values (above 0.96 in both collections), indicating a strong tendency to recover most true homologous pairs. However, its Precision was substantially lower (approximately 0.685), consistent with the excess of predicted pairs observed in Table 10 and indicative of frequent merging of unrelated genes into the same family. This trade-off resulted in F1 scores of approximately 0.80 for both collections. PanTools showed moderate Precision (approximately 0.79) but the lowest Recall among the three tools, which dropped markedly from 0.6859 in collection A to 0.4609 in collection B. This decline is consistent with the high degree of family fragmentation noted earlier: as the number of genomes increases, PanTools splits families more aggressively, thereby missing an increasing fraction of true homologous pairs. Consequently, its F1-score decreased from 0.7363 to 0.5825, highlighting a sensitivity to dataset scale.

**Table 11:** Precision, Recall, and F1-score for each compared tool.

Collection	PanDelos-plus			PPanGGOLiN			PanTools		
	Prec.	Recall	F1	Prec.	Recall	F1	Prec.	Recall	F1
A	0.9594	0.9676	0.9635	0.6847	0.9761	0.8048	0.7946	0.6859	0.7363
B	0.9782	0.9570	0.9674	0.6855	0.9623	0.8007	0.7914	0.4609	0.5825

Classification metrics computed on gene pairs. Precision measures the fraction of predicted homologous pairs that are correct; Recall measures the fraction of true homologous pairs that were identified; F1 is the harmonic mean of Precision and Recall.

Table 12 explicitly reports TP, TN, FP and FN values for each evaluated tool for each collection. As expected, all tools produced very high TN counts, reflecting the combinatorial nature of pairwise comparisons across entire genomes, where the vast majority of gene pairs are non-homologous. PanDelos-plus achieved the most balanced TP-to-FP ratio across both collections, with relatively low FP and FN counts. In Collection B, it correctly identified over 25 million true pairs while producing only 559,235 false positives, the lowest among all tools. PPanGGOLiN consistently recovered the highest number of true positives (6,325,212 in Collection A and 25,185,313 in Collection B), explaining its high Recall. However, this came at the cost of substantially elevated FP counts, reaching over 11.5 million in Collection B. This confirms that PPanGGOLiN tends to over-merge gene families, incorrectly grouping non-homologous genes together. PanTools exhibited the highest FN values in both collections, with over 14 million missed pairs in Collection B, consistent with its aggressive family fragmentation and the steep Recall drop observed earlier. While its FP counts were moderate, the large number of missed true homologous pairs explains its low F1-score, particularly on the larger dataset.

**Table 12:** TP, TN, FP and FN values for each tool.

Collection	Metric	PanDelos-plus	PPanGGOLiN	PanTools
A	TP	6,270,221	6,325,212	4,445,004
	FP	265,300	2,912,415	1,148,902
	FN	210,140	155,149	2,035,357
	TN	36,684,356,725	36,675,750,239	36,686,994,797
B	TP	25,044,271	25,185,313	12,061,165
	FP	559,235	11,555,151	3,180,065
	FN	1,126,501	985,459	14,109,607
	TN	149,407,974,698	149,392,605,298	149,414,101,028

TP, TN, FP and FN values for each tool on both collections. TP: homologous pairs correctly identified; FP: non-homologous pairs incorrectly predicted as homologous; FN: true homologous pairs missed; TN: correctly identified non-homologous pairs.

## Discussion

The central result of this work is that the PanDelos methodology can be made to scale to population-level genome collections without altering the homology criterion on which it rests. PanDelos-plus reproduces the behaviour of the original algorithm while reducing peak memory by up to 96.5% and wall-clock time by up to 13.9× on the real datasets. The memory reduction is attributable entirely to the replacement of the enhanced suffix array with the multiplicity vector: at a single thread, PanDelos-plus already requires 0.61 GB on *Escherichia coli* against 20.2 GB for PanDelos, and 2.8 GB against 27.2 GB on the 50-genome synthetic collection. The speedup, by contrast, is attributable entirely to parallelism. At one thread PanDelos-plus is in fact slower than PanDelos on every dataset tested, by a factor ranging from 1.2× (*Salmonella enterica*) to 2.2× (*Mycoplasma*). The enhanced suffix array is a serially efficient structure, and abandoning it carries a measurable serial cost; what it does not tolerate is decomposition, since its auxiliary indices are shared across the whole concatenated sequence set and scale with total input length. The multiplicity vector trades a constant-factor serial penalty for two properties the suffix array cannot provide: a per-gene memory footprint independent of collection size, and independence between units of work that permits near-ideal strong scaling. On the real datasets this trade is recovered by the second thread, and on the 50-genome synthetic collection PanDelos-plus overtakes PanDelos at two threads.

The scaling behaviour with collection size follows directly from the structure of the problem. An all-against-all comparison over *n* genomes requires n(n−1)/2 genome pairs, moving from 50 to 600 genomes multiplies the number of genomes by 12 but the number of pairs by approximately 147.The observed runtime increase, from approximately 1,963 s to approximately 225,000 sec., is a factor of roughly 115, that is, slightly sub-quadratic in the number of genomes once the pairwise structure is accounted for, and therefore consistent with a per-pair cost that does not derade as the collection grows. Mean gene content per genome is essentially constant across Collections 1–12 (5,397–5,448 genes), so the growth is not confounded by increasing genome size. Peak memory, by contrast, grows approximately linearly, from 2.7 GB to 36 GB, reflecting the fact that multiplicity vectors are stored per gene rather than per pair.

The comparison with PPanGGOLiN and PanTools indicates that the accuracy advantage of the dictionary-based criterion is preserved under parallelization and, importantly, is stable with respect to collection size. PanDelos-plus attains F1 scores of 0.9635 and 0.9674 on Collections A and B respectively, with the two component metrics remaining balanced (precision 0.9594 and 0.9782; recall 0.9676 and 0.9570). The two comparison tools fail in opposite and characteristic ways. PPanGGOLiN recovers the largest number of true homologous pairs in both collections but does so with precision near 0.685, producing over 11.5 million false positive pairs on Collection B; this is the signature of over-merging, in which a small number of erroneous fusions among large, near-core families generates a disproportionate number of spurious pairs. PanTools shows the converse: moderate precision with recall falling from 0.6859 to 0.4609 as the collection doubles, consistent with progressively aggressive family fragmentation and with its production of 28,363 families against an expected 10,044. That PanTools degrades with scale while PanDelos-plus does not is relevant beyond a simple ranking of tools, since it implies that benchmark results obtained on small collections may not transfer to the collection sizes that pangenomic studies increasingly employ.

These observations bear on a working assumption in the field: that the classification of gene families as core, dispensable, or singleton is a stable property to be estimated, with additional genomes serving mainly to refine the estimate. Our diffusivity analysis suggests a more specific picture. The transition from Reduced to Complete sets is overwhelmingly additive: across all four species groups, the dominant category of change is Enlarged, partially enlarged families number at most four per dataset, and collapsed families remain rare. Because this stability is assessed by comparing the method against itself at two sampling depths, it does not depend on any simulated ground truth. It follows that the substantial reclassification observed across diffusivity classes — most pronounced in *Mycoplasma*, where family count rises from 8,743–13,268 as genomes increase from 21 to 64 — reflects the change in the sampled collection rather than instability in gene family assignment. Core, dispensable, and singleton are thus properties of a sample, and their estimation is limited less by clustering accuracy than by how many genomes a method can process. For taxa with open pangenomes, where novel family discovery does not saturate, computational scalability is not a convenience but a precondition for a good, reasonable classification.

## Conclusions

PanDelos-plus represents a substantial enhancement of the original PanDelos algorithm, addressing key limitations in runtime efficiency, memory usage, and parallel execution. By leveraging a gene-centric approach, lightweight data structures, and thread-level parallelism, PanDelos-plus reduces execution time and memory requirements compared to PanDelos. PanDelos-plus extends beyond a straightforward parallelization effort. The replacement of the enhanced suffix array with a lightweight multiplicity vector data structure represents a novel engineering solution. To the best of our knowledge, no comparable structure in the literature provides equivalent advantages for parallel k-mer similarity computation with such a reduced memory footprint. Overall, PanDelos-plus extends the applicability of the PanDelos framework to population-scale comparative genomics, enabling large-scale pangenome reconstruction on parallel architectures.

Evaluation on a synthetic dataset of 50 genomes shows that PanDelos-plus already surpasses the single-threaded performance of PanDelos when using only two threads, with execution time decreasing as the number of threads increases up to 32. Peak memory usage remains essentially constant across thread counts, indicating efficient parallel utilization of computational resources. For this fixed genome set, PanDelos-plus achieves up to 18× speedup relative to single-threaded execution, showing strong, though sub-ideal, parallel scaling. When scaling the number of genomes from 50 to 600 at 32 threads, peak memory usage grows roughly linearly with dataset size, remaining under 40 GB for 600 genomes. Execution time, however, increases faster than linearly, rising from approximately 0.5 hours for 50 genomes to over 60 hours for 600 genomes. This indicates superlinear scaling of computational cost with increasing genome collection size, reflecting the combinatorial growth of gene families and the associated computations. These results demonstrate that PanDelos-plus efficiently exploits parallelism for fixed dataset sizes and maintains predictable memory growth with increasing genome count. While execution time increases faster than linearly for larger datasets, the tool remains applicable to moderate- to large-scale pangenome analyses within practical computational resources.

As a collateral but key result, the new methodology can more effectively refine the aggregation of genes into families by leveraging homology information from an increased number of analysed genomes. In fact, the classification of a specific family as core, dispensable, or singleton is sensibly affected by the availability of such information.

## Supporting information

S1 File Real dataset. Complete list of the genomes used in the real dataset experiments, including accession numbers and relevant metadata.
(XLSX)

S2 File Synthetic dataset. Complete list of the genomes used in the synthetic dataset experiments, including root genome and htg pool details. All genomes are listed with their respective accession numbers and relevant metadata.
(XLSX)

S3 File Hardware and software specifications. Detailed technical specifications of the experimental environment. This includes the complete hardware description (processor model, architecture topology, cache hierarchy, memory configuration) as well as the software build environment, including the compiler version, the exact optimisation flags used, and the specific commit hash of the PanDelos-plus version tested.
(PDF)
