# Supplementary material for: PanDelos-plus: A parallel algorithm for computing sequence homology in pangenomic analysis
Source: PLoS Comput Biol. 2026 Sep 17;22(9):e1014724. doi: 10.1371/journal.pcbi.1014724 (PMC13607995; doi:10.1371/journal.pcbi.1014724)
Supplement: Supplementary file 1 [file pcbi.1014724.s003.pdf]

S3_File: hardware and software specifications

Overview

This document provides detailed hardware specifications for the computing node used to perform the experiments reported in the manuscript. The node is part of a University High-Performance Computing (HPC) cluster.

To ensure reproducibility, we report below the processor architecture, cache hierarchy, memory configuration, and raw system diagnostic output.

System configuration summary

The experiments were conducted on a dual-socket node equipped with AMD EPYC 7282 processors. **Note on parallelism:** Although the processor supports Simultaneous Multithreading (SMT), the specific node configuration has SMT disabled. Consequently, the 32 physical cores correspond to 32 logical threads available to the operating system.

**Component**	**Specification**
**Processor Model**	AMD EPYC 7282 16-Core Processor
**Architecture**	2 Sockets × 16 Physical Cores (Total: 32 Physical Cores)
**Logical Cores**	32 (SMT/Hyper-threading is **Disabled**: 1 thread per core)
**Clock Speed**	Base: 2.80 GHz (Core Performance Boost: Enabled)
**L1 Cache**	64 KB (32 KB Data + 32 KB Instruction)
**L2 Cache**	512 KB
**L3 Cache**	16 MB
**RAM**	256 GB DDR4

Table 1: Hardware specifications of the computing node.

Raw system output

Below is the raw output of the `lscpu` command executed on the node, confirming the architecture topology and cache sizes.

```
Architecture:          x86_64
CPU op-mode(s):        32-bit, 64-bit
Byte Order:            Little Endian
CPU(s):                64
On-line CPU(s) list:   0-31
Off-line CPU(s) list:  32-63
Thread(s) per core:    1
Core(s) per socket:    16
Socket(s):             2
NUMA node(s):          2
Vendor ID:             AuthenticAMD
```

```
CPU family:           23
Model:                49
Model name:           AMD EPYC 7282 16-Core Processor
Stepping:             0
CPU MHz:              2800.000
CPU max MHz:          2800,0000
CPU min MHz:          1500,0000
BogoMIPS:             5589.32
Virtualization:       AMD-V
L1d cache:            32K
L1i cache:            32K
L2 cache:             512K
L3 cache:             16384K
NUMA node0 CPU(s):    0-15
NUMA node1 CPU(s):    16-31
```

Software build environment

**Component**	**Detail**
**Compiler**	g++ (GCC) 4.8.5 20150623 (Red Hat 4.8.5-44)
**PanDelos-plus Optimization profile**	Custom (see `compile.sh` in repository)
**Source code version**	PanDelos-plus, commit `3c5cee392eaa5044cac01a58e00419a4b4fc2407`; PanProva, commit `3d3bbfc8c13bae70747bde9e2c872b1b87c342ec`

Table 2: Software build configuration.

Note on optimisation flags. The build uses `-O1` as a baseline with selective additions from the `-O2`/`-O3` levels, specifically targeting inter-procedural analysis, loop transformations, and modulo scheduling, while disabling aggressive inlining and auto-vectorisation. All benchmarks reported in this paper were executed under this configuration.

PANPROVA configuration

The synthetic datasets used in the experiments were generated using PANPROVA, a simulator for prokaryotic pangenome evolution. The exact version used corresponds to the commit reported in Table 2.

The simulation was executed using the script `examples/test_3/run_example.sh` available in the PANPROVA repository. This script invokes `PANPROVA.sh`, which defines all simulation parameters, including the random seed set to `rseed="123456"`.
