# Supplementary material for: PanDelos-plus: A parallel algorithm for computing sequence homology in pangenomic analysis
Source: PLoS Comput Biol. 2026 Sep 17;22(9):e1014724. doi: 10.1371/journal.pcbi.1014724 (PMC13607995; doi:10.1371/journal.pcbi.1014724)
Supplement: Supplementary file 2 [file pcbi.1014724.s004.pdf]

Response to Reviewers

We would like to express our sincere gratitude to the Editors and the Reviewers for their time, effort, and constructive feedback on our manuscript, "PanDelos-plus: A parallel algorithm for computing sequence homology in pangenomic analysis". We found the feedback to be highly insightful, and addressing these points has significantly strengthened the quality, clarity, and depth of our work.

In this document, we provide a comprehensive, point-by-point response to each of the questions and concerns raised during the review process. For ease of reading, the reviewers' questions and remarks are identified by the tag Comment, and our detailed replies and explanations are identified by the tag Answer. As noted in our cover letter, all added or substantially changed text within the revised manuscript has been highlighted in red to facilitate your review.

Reviewer #1: Review is uploaded as an attachment.

This manuscript describes PanDelos-plus, an improved version of the PanDelos bacterial gene set pangenomics tool. Compared to PanDelos, PanDelos-plus adds parallelization and reduces memory usage. PanDelos-plus seems like a great improvement over the original PanDelos, and indeed seems to be a complete reimplementation in a different language. I think the tool will be of broad interest among bacterial pangenomics researchers, though I admit that I myself am not one. The experiments presented seem well-designed to compare the performance of the new tool to the old one.

However, there are several issues with the writing of the manuscript, where the mathematical formulas are wrong, the text is written confusingly, or the description in the text doesn't properly characterize the results presented in the figures.

In terms of major concerns, I only have a couple of larger-scale problems with the manuscript that might potentially require more experiments.

[Comment R1-1] First, the authors only look at wall-clock time (seconds), and not CPU time (CPU-seconds). The single-threaded case in the lower panel of Figure 3, where PanDelos runs faster than PanDelos-plus, suggests that the new implementation might be trading off worse CPU efficiency for better memory efficiency, or that there might be important overhead added by the multi-threading logic. This is a valid design choice, but it's important to actually characterize it: how does total CPU time used increase as the number of threads is increased, and at what point (if any) does the overhead from synchronization and straggling threads with nothing to do begin to get unacceptably in comparison to the CPU-time spent doing useful work? How does the total CPU time at various thread counts compare to the total CPU time of original PanDelos? If someone has a lot of pangenomic analysis jobs to do, and needs to pack them efficiently onto available computing resources, these are important measurements to have.

[Answer] We agree with the reviewer that CPU time has to be taken into account when evaluating parallel implementation. For this reason, we added Table 4 that shows CPU time of PanDelos-plus on varying the number of threads. These values can be compared with the wall-clock time of Pandelos reported in Table 3, since the original serial version does use parallel code and its CPU time is comparable with its wall-clock time.

[Comment R1-1]Second, the authors compare PanDelos-plus only against the original PanDelos, not against any competing tools. I don't know of any in particular that I would expect to see, but I would suspect at least one new competitor is available now that was not available when the original PanDelos came out, or else maybe one of the tools that was originally tested against PanDelos supports a multithreaded mode that could be compared against here. It looks like, since the original PanDelos came out, potentially relevant tools called "Panakeia" and "PanTools v3" have been published. I also have a number of smaller-scale concerns.

[Answer] We thank the reviewer for this comment. To address this point, we have conducted a completely new set of comparative evaluations and introduced a dedicated Supplementary File (File_S5).

In this new analysis, we evaluate the clustering performance of PanDelos-plus against PanTools and PPanGGOLiN. We have generated synthetic datasets with PANPROVA (where the exact ground truth of gene families is known) and we compared the families predicted from the tools and compared using Precision, Recall, and F1-score metrics calculated on the set of retrieved homology relationships among genes.

Moreover, we reported an additional state of the art for what concerns tools (Panakeia, PanTA, PanTools v3, PPanG and others reported in the review "Pangenomes: new tools for ecological and evolutionary genomics") that do not directly compute sequence homology, thus gene families aggregation, but that allow the users to perform downstream analysis on top of it.

[Comment R1-2]- The Author Summary says that "I developed PanDelos-plus", but it probably should be "we".

[Answer] We thank the reviewer for pointing out this error. We have corrected the Author Summary by replacing "I" with "we".

[Comment R1-3]- The Author Summary also says that the new tool is "making large-scale bacterial pangenome analysis accessible for routine use in everyday research". Is it really plausible that a bacterial pangenomics researcher would actually want to fully rebuild their orthology graph on a daily basis? Or is the increased efficiency instead expected to open up some new kind of analysis that was previously impractical? Or is the contribution really that something that was previously difficult and slow is now fast, simple, and easy, making researchers' lives easier?

[Answer] We thank the reviewer for this observation. We agree that the original phrasing overstated the practical usage scenario. Our intention was not to suggest that researchers would routinely rebuild orthology graphs on a daily basis, but rather to emphasize that the improved efficiency lowers computational barriers and enables the analysis of substantially larger genome collections on standard multicore systems.
We have revised the Author Summary to clarify that the primary contribution of PanDelos-plus is improved scalability and reduced computational requirements, facilitating large-scale pangenome reconstruction without the need for specialized high-performance computing infrastructure.

[Comment R1-4]- Starting on line 4, but more or less throughout the manuscript, there seems to be extra space before each citation number.

[Answer] We thank the reviewer; there were in fact two spaces before each citation number. We corrected them to have only one space before each citation number.

[Comment R1-5]- On line 12, there's a discussion of singleton genes in the pangenome of a set of genomes. Since PanDelos-plus seems aimed at expanding the maximum size of the genome set that it is practical to work with, what does sampling the same phylogenetic group with a larger number of

genomes do to the concept of singleton genes? If you add an additional sequenced genome that shares a recent ancestor with one already sampled, it might turn what was a "singleton" gene into a "dispensable" gene by also having a copy. Will these concepts retain their usefulness as the sizes of practical pangenomes expand, or will new ways of thinking about how a gene relates to a pangenome be required?

[Answer] We thank the reviewer for raising this important conceptual issue. We agree that core, dispensable, and singleton classifications are inherently dependent on the sampled genome set. As additional genomes from the same phylogenetic group are incorporated, gene families previously classified as singletons may be reclassified as dispensable if homologs are identified in newly sampled genomes. We have revised the Introduction to explicitly clarify this sampling dependence.

We further relate this behavior to the distinction between open and closed pangenomes, emphasizing that scalable reconstruction methods are essential for accurately characterizing how gene family frequencies evolve as genome sampling increases. To show such a behavior, we conducted tests aimed at evaluating how gene families vary on including more genomes in the analysis.

[Comment R1-6]- On line 16, the word "transitions" needs a comma after it, and might also need to be a different word, because I don't know what it means here. Maybe it is meant to mean "horizontal transfer events"?

[Answer] We agree with the reviewer, the sentence was unclear. We have revised the sentence to explicitly describe that gene sequences accumulate mutations during replication and evolution, including nucleotide substitutions, insertions, and deletions, and we removed the ambiguous term "transitions." This clarifies the intended meaning without implying horizontal transfer where it is not intended

[Comment R1-7]- On line 15-17, the whole paragraph seems to be there to note that mutations to genes change the sequences of those genes, and that this is important for pangenomic analysis. This might not need to be said at all, and could probably be said more straightforwardly.

[Answer] We agree with the reviewer that the original formulation was overly verbose and stated a well-known biological fact in a convoluted way. We have therefore simplified and clarified this part of the paragraph to more directly link sequence variation to its methodological implications for pangenome inference. The revised text now states that gene sequences accumulate mutations leading to polymorphisms, and that this impacts the identification and clustering of homologous genes into families, which is the relevant point for pangenomic analyses.

[Comment R1-8]- On lines 18-19, I don't understand "strong evolutionary selection of core genes even if transmitted with few modifications", and how it makes recognizing homologs difficult. I would understand strong selection on core genes as being generally in favor of conserving the existing sequence. Are they trying to make the point that core genes can sometimes be subject to positive selection that makes them accumulate mutations quickly? But then why say they were "transmitted with few modifications"?

[Answer] We thank the reviewer for highlighting this ambiguity. We agree that the original wording was conceptually unclear and potentially misleading. Our intention was not to imply that core genes are under positive selection leading to rapid sequence change, but rather to emphasize that core gene families are evolutionarily conserved, which can complicate homology inference when combined with sequence-level variation and clustering thresholds in pangenome pipelines.

We have therefore corrected both the biological interpretation and the phrasing. The revised text replaces the ambiguous reference to "strong evolutionary selection" with a clearer statement about high conservation of core gene families, and explicitly connects this to challenges in clustering homologous genes into families.

The sentence now reads:

"These mutations, together with the high conservation of core gene families, complicate the accurate identification and clustering of homologous genes."

This formulation more accurately reflects the methodological challenge: core genes tend to be conserved at the functional and family level, yet sequence-level variation and clustering heuristics can still make homolog recognition non-trivial, especially across phylogenetically diverse genomes.

[Comment R1-9]- On line 22, saying "the importance of" something is "revealed in terms of" something else doesn't really seem to mean anything. They really mean that that pangenome information "has" clinical applications.

[Answer] We have revised the sentences to state clearly that pangenome information has important clinical applications.

[Comment R1-10]- Lines 23 to 27 have bad parallelism; the things in the list are not all the same kind of phrase.

[Answer] The list of applications has been restructured for grammatical parallelism, using consistent infinitive phrasing for each item (to identify, to detect, to distinguish, to investigate) to improve clarity and readability

[Comment R1-11]- Line 28 describes "retrieval" of a pangenome, but seems to really mean something more like "construction" of a pangenome.

[Answer] We thank the reviewer for the comment regarding the terminology of "retrieval" versus "construction." We agree that, from a computational perspective, the pangenome is constructed as a model or representation derived from genome sequences. However, biologically, the pangenome refers to the set of gene families present in a population and thus exists independently of any specific computational procedure.

To clarify this distinction and avoid ambiguity, we have revised the text to explicitly state that the pangenome is a biologically defined entity, while its characterization in practice requires computational reconstruction from genomic data. This change aligns the wording with both biological and computational perspectives.

[Comment R1-12]- On line 35-36, competing tools are mentioned, but the newest is from 2021; has nothing relevant come out in the past 4 years?

[Answer] We agree with the reviewer. We improved the review section by adding more recent pangenomic tools: Panakeia, PanTA, PanTools v3, PPanG and a 2025 review “Pangenomes: new tools for ecological and evolutionary genomics”.

[Comment R1-13]- On line 49, "that" should be "which".

[Answer] We thank the reviewer for pointing out this grammatical error. We have corrected " that " to " which " in the revised manuscript.

[Comment R1-14]- On lines 76-77, the math expression after "of length" is missing, and the rest of the sentence seems shuffled or garbled. "can occurs" is definitely wrong. Maybe the part about |s| is out of place?

[Answer] We apologize for the garbled sentence and the grammatical error. We have rewritten the paragraph to clearly define the sequence length notation and to correctly state the number of k-mers contained in a sequence (correcting the formula to $|s|-k+1$).

[Comment R1-15]- In equation 1, ρ is bounded above by |g|, not |g - k|, so the set appears to include partial k-mers that go off the end of g.

[Answer] The reviewer is correct regarding the boundary condition. We have corrected Equation 1 by adjusting the upper bound of p to $|g| - k + 1$. This ensures that the substring index never exceeds the sequence length |g|.

[Comment R1-16]- Also in equation 2, the same upper bound is used on ρ, and it's again allowed to produce k-mers that leave the end of g.

[Answer] We thank the reviewer for this correction. We have fixed the upper bound of p to $|g| - k + 1$ as done in the previous point.

[Comment R1-17]- On line 88, "Dictionary-based approach" seems to be missing "The".

[Answer] We thank the reviewer for this correction. We have added the missing article 'The' as suggested.

[Comment R1-18]- The claim on line 89 that this formula gives the best "resolution" of k needs to be explained. Do they mean the best "value" of k? Best by what metric or for what purpose? Why does neither gene length nor GC content matter? I understand that this is cited to a couple previous publications, but these appear to be publications from the same group, so the explanation might need to be summarized here again.

[Answer] We thank the reviewer for pointing this out. We have clarified the meaning of "best resolution" and summarized the rationale for the formula in the manuscript.

The formula for k provides the value of k that optimally balances uniqueness and coverage of k-mers across all genomes in the dataset. In dictionary-based pangenomic approaches, k must be large enough that most k-mers are unique, reducing spurious matches between unrelated regions, but not so large that sequence overlap is lost. The cited studies show theoretically that this logarithmic formula achieves this balance, yielding the best resolution of genome content for constructing pangenomic dictionaries.

This definition of "best resolution" therefore refers to the ability to distinguish gene families and homologous regions accurately. Gene length and GC content are not explicitly included because the metric is derived from the total number of nucleotides across the dataset (sum_i over |G_i|) and the size of the alphabet gamma (|Γ|), which already account for sequence complexity and information content. Empirically, this approach has been validated across multiple datasets, providing k values that optimize family discrimination and minimize redundancy in the dictionary.

As suggested we added a summarized explanation before the cited studies:
"This formula provides the value of k that maximizes the resolution of genome content, i.e., it balances uniqueness and coverage of k-mers across the dataset, enabling accurate discrimination of gene families and homologous regions. Gene length and GC content are not directly included because the metric is based on the total sequence information in the dataset and the alphabet size, which together capture sequence complexity. These definitions and rationale follow the studies reported in Bonnici 2016 and Manca 2017"

[Comment R1-19]- Lines 92-93 have a sentence fragment.

[Answer] We thank the reviewer for catching this grammatical error. We have rephrased the text to merge the fragment with the following sentence, ensuring a complete grammatical structure.

[Comment R1-20]- On line 95, "k-mers multiplicity" should be "k-mer multiplicity"

[Answer] We thank the reviewer for pointing out this grammatical error. We have corrected " k-mers multiplicity " to " k-mer multiplicity " in the revised manuscript.

[Comment R1-21]- In equation 5 (and later), things like "min" and "max" andf "and" are typeset in italic, but probably should by typeset in Roman (normal) instead.

[Answer] We thank the reviewer for the comment. We changed the typeset to Roman as suggested.

[Comment R1-22]- On line 104, the sentence is missing a subject (perhaps "they")?

[Answer] We thank the reviewer for noticing this grammatical error. We have added the missing subject 'they' to the sentence as suggested.

[Comment R1-23]- Can't the "correct" value of the θ threshold be derived from the value of the τ threshold, based on the length ratio at which two strings would no longer have the necessary amount of Jaccard similarity even if one were a substring of the other? If not, what value of θ is actually used?

[Answer] We agree with the reviewer that, in an idealized scenario where one sequence is a near-identical substring of the other, the maximum achievable Jaccard similarity is bounded by the length ratio. However, in real biological data, sequence divergence due to substitutions and indels substantially alters k-mer composition. As a result, Jaccard similarity cannot be reliably bounded by length ratios alone.

For this reason, the length-based threshold θ cannot be derived from the Jaccard threshold τ in a principled way. Instead, θ is used as an independent, computationally inexpensive prefilter to discard gene pairs with highly unbalanced lengths that are unlikely to be homologous, while τ captures sequence-level similarity after accounting for k-mer overlap under mutation. The two thresholds therefore play complementary roles rather than being redundant.

As suggested, we added the value of θ used which is of 0.5 by default and can also be adjusted by the user.

[Comment R1-24]- I found the explanation at lines 115-118 confusing; is τ meant to be 1/k? It is supposed to be computed theoretically, but the actual expression to use is not provided.

[Answer] We agree with the reviewer that the explanation in the original manuscript was confusing. We have revised the presentation to improve the logical flow of the section and corrected the terminology used for the filter p_k(s --> t), which was previously and incorrectly referred to as a "directional Jaccard similarity." In the revised version, p_k(s --> t) is correctly described as a measure of dictionary intersection coverage of sequence *ss*s with respect to *tt*t, consistent with the terminology adopted in the original PanDelos paper in the Methods section ("Selection of candidate gene pairs").

Regarding the choice of the threshold τ=2/k, we now explicitly refer to the theoretical rationale provided in the original PanDelos work and cite it accordingly. In our study, we retain the same value τ=2/k as the filtering threshold in order to remain consistent with the original methodology.

[Comment R1-25]- The sentence starting on line 119 should be a new paragraph; the explanation of filtering based on τ has been abandoned and a new point is being made.

[Answer] As discussed in our response to the comment on lines 115–118, we have rewritten this part of the manuscript to improve the clarity and logical organization of the explanation. In the revised version, we first introduce the second filtering criterion based on dictionary intersection coverage, then formally define the condition under which two genes gi and gj are considered homologous candidates (i.e., both directional coverages must exceed the threshold τ), and finally present the rationale for the choice of the threshold value τ =2/k, with reference to the original PanDelos methodology.

[Comment R1-26]- The sentence on lines 119-123 is scrambled. Maybe "This" was meant to be "to"? You can't say "is introduced an additional filtering step" because that leaves the subject position of the

sentence empty. It has to be "an additional filtering step is introduced" or "we introduced an additional filtering step".

[Answer] We apologize for the scrambled sentence. We have rephrased it as suggested, changing 'This' to 'To' and fixing the sentence structure to correctly introduce the filtering step.

We rewrote the sentence:

“This ensures that the similarity is not only high, but also reciprocal, which is a desirable property in homolog detection, is introduced an additional filtering step based on the directional Jaccard similarity defined as:”

As:

“To ensures that the similarity is not only high, but also reciprocal, which is a desirable property in homolog detection, we introduced an additional filtering step based on the directional Jaccard similarity defined as:”

[Comment R1-27]- The directional Jaccard similarity needs to be not only mathematically defined but also explained; it appears to be the fraction of k-mer occurrences in s that are of kmers also present in t, but this isn't written out, so it's not clear to the reader what this function is meant to compute.

[Answer] We thank the reviewer for pointing this out. We revised the text to explain the formula, now called dictionary intersection coverage and not directional Jaccard similarity.

In particular, we now clarify that p_k (s->t) represents the fraction of all k-mer occurrences in s that correspond to k-mers also present in t, and it quantifies the proportion of the k-mer content of s that is covered by t.

[Comment R1-28]- The sentence on lines 124-125 is missing a colon before the math.

[Answer] We thank the reviewer for the correction. We have added the missing colon before the equation.

[Comment R1-29]- On line 125, is the τ used here the same as the one that it was implied we use to filter out potential matches based on the normal, symmetrical Jaccard distance? Or is that filter only used here and the implied filtering step from the explanation of the threshold doesn't actually happen? In general, the filtering logic could be described more explicitly.

[Answer] We thank the reviewer for pointing out that the filtering logic was not sufficiently explicit in the original presentation. We clarify that the threshold τ is not applied to the generalized Jaccard similarity. Instead, τ is the threshold used in the dictionary intersection coverage filter pk(s-->t), which acts as a pre-filter to select candidate homologous gene pairs prior to computing the Jaccard similarity.

Similarly, the length-based threshold θ is also applied before Jaccard computation. Only gene pairs that pass both the length-based filter θ and the coverage-based filter τ are subsequently evaluated using the generalized Jaccard similarity to identify Best Hits and Bidirectional Best Hits.

To avoid this ambiguity, we have added a paragraph before the explanation of the Jaccard similarity, stating that both θ and τ are pre-filtering criteria and that the Jaccard similarity is computed only for gene pairs passing these filters.

[Comment R1-30]- On line 129, sharing "the highest Jaccard similarity" is ambiguous to me; does it mean among the set somehow, or does it mean between each gene in the set and the gene whose Best Hit set we are talking about? And do we mean the normal, symmetrical Jaccard, or the directional Jaccard that was just defined?

[Answer] We thank the reviewer for pointing out this ambiguity. The "highest Jaccard similarity" is referring to the generalized Jaccard similarity, the directional Jaccard similarity was corrected to "dictionary intersection coverage" in the prefiltering section, so now there is no ambiguity since there is only the generalized Jaccard similarity.

In our method, the Best Hit of a gene is defined with respect to a specific query gene: it consists of the gene(s) in the other genome that achieves the highest similarity to that query gene, not among themselves.

We clarified by fixing the sentence preceding the Best Hit formula: "To identify homologous genes between two genomes, Gi and Gj, the Jaccard similarity is computed for all ordered pairs of their respective genes. For a gene g_{i,l} in Gi, its Best Hit in genome Gj is defined as the set of genes in Gj that maximize the Jaccard similarity from g_{i,l} to genes in Gj."

[Comment R1-31]- Around equation 9, the Bidirectional Best Hit concept could be explained better. If two genes are in each others' Best Hit sets, they are Bidirectional Best Hits. But is this expected to always form complete bipartite graphs between the genes in one genome and the genes in the other, making a sort of equivalence class? Or can you have a gene that is Bidirectional Best Hits with two genes in the second genome, but only one of those two is Bidirectional Best Hits with some other gene in the first genome?

[Answer] We thank the reviewer for the insightful comment regarding the structural properties of the Bidirectional Best Hit (BBH) relation. BBHs in our framework are defined locally and pairwise as reciprocal best-hit relationships between genes across two genomes and are not intended to define equivalence classes. In particular, BBHs are not transitive and do not necessarily form complete bipartite components between genomes. A gene in one genome may be in BBH relationship with multiple genes in another genome, and these genes may, in turn, have different BBH partners in the first genome.

To avoid ambiguity, we have revised the manuscript to clarify that BBHs induce a general gene–gene similarity graph, in which edges represent reciprocal best-hit relationships. This graph representation is then used as input to the subsequent network-based refinement step (community detection) for extracting coherent gene families.

[Comment R1-32]- On line 146-147, it says each "comparison task" is an "independent" unit of work, but actually in Algorithm 2 a whole row of many comparisons against one gene serves as a unit of work.

[Answer] We thank the reviewer for pointing out this imprecision; we have revised the sentence to explicitly state that a unit of work consists of a whole row of comparisons. The sentence:

"... each comparison task is submitted as an independent unit of work ..."

Becomes:

"... the workload is partitioned such that each unit of work corresponds to a whole row of comparisons (i.e., one gene compared against the entire target genome) ..."

[Comment R1-33]- On line 151, "leverages on" should probably just be "leverages".

[Answer] We thank the reviewer for pointing out this grammatical error. We have corrected "leverages on" to " leverages" in the revised manuscript.

[Comment R1-34]- On lines 154-156, it says PanDelos has 5 key steps and then describes two things. Are those two things two of the 5 steps? Are they two phases, each of which contains some of those 5 steps? Does the word "integrate" mean that the important thing about PanDelos is that actually it combines these two things into a single one of the 5 steps?

[Answer] We thank the reviewer for highlighting the ambiguity in the description of the PanDelos workflow. In the original manuscript, the relationship between the five procedural steps and the two conceptual components of the method (k-dictionary–based candidate selection and network-based refinement) was not sufficiently explicit.

We have revised this part of the text to clarify that the five steps are organized into two conceptual phases: (i) an initial candidate selection phase based on k-dictionaries, and (ii) a subsequent network-based refinement phase. Moreover, each of the five steps is now described in a separate sentence, and we explicitly state how each step maps to one of the two phases. These changes make the overall workflow clearer and remove the ambiguity regarding the correspondence between procedural steps and conceptual components.

[Comment R1-35]- On line 158, does the filter for minimum k-dictionary overlap ever lose Best Hit or Bidirectional Best Hit results, or is it provably throwing out only comparisons that cannot give rise to them?

[Answer] This filter is described in the original PanDelos paper in the Methods section. The filter is a theoretically motivated heuristic: it requires that at least a fraction 2/k of the k-mers in each gene overlap, which is the minimum necessary to meaningfully reconstruct gene sequences from their k-mer dictionaries. While this threshold does not guarantee that no true homologs are ever excluded, gene pairs below this cutoff cannot be reliably reconstructed or compared, so the filter effectively eliminates only gene pairs with no realistic chance of forming Best Hits or Bidirectional Best Hits. In practice, this filter reduces computational cost without noticeably affecting homology detection.

[Comment R1-36]- On line 161, the manuscript is talking about a "homology" network, but I would think that with the bidirectional constraint this is really meant to be a network of orthologs specifically, right?

[Answer] We thank the reviewer for the comment regarding the term “homology network.” We agree that the network is not limited to orthologous relationships. To clarify, the network includes orthologous, paralogous, and xenologous relationships, with singletons often arising from horizontal gene transfers (xenologues). We have updated the text accordingly to accurately reflect the composition of the homology network.

[Comment R1-37]- On line 164, "combines" should probably be "combining".

[Answer] We thank the reviewer for pointing out this grammatical error. We have corrected " combines " to " combining " in the revised manuscript.

[Comment R1-38]- On line 165, it says a structure "drastically improves" some performance metrics; what is the improvement relative to?

[Answer] We thank the reviewer for pointing out that the statement regarding performance improvement was made without an explicit reference. The original sentence (“drastically improve the efficiency of substring comparisons and overall runtime performance”) was imprecisely worded and potentially misleading. Our intention was not to claim a quantified performance improvement, but rather to highlight the computational efficiency of the adopted data structure.

We have therefore revised the sentence to read as follows:

“The core algorithm of the PanDelos pipeline relies on the enhanced suffix array [24] to compute sequence similarity. This structure combines a suffix array [25] with the longest common prefix (LCP) array. This indexing scheme enables efficient enumeration of shared substrings and k-mers across genes.”

[Comment R1-39]- On line 169, it says combining the suffix array and the LCP allows for "fast and accurate similarity detection", but it doesn't really explain how, or why using them together is an important insight over using one or the other alone. I'm not sure this is really relevant; maybe this part should be rewritten to be more like background and not read like it's reporting but not really explaining a key innovation.

[Answer] We thank the reviewer for pointing out that the original text suggested an innovation without sufficiently explaining its significance. We agree that this part should serve as background rather than appear to introduce a novel methodological contribution.

We have therefore revised the paragraph to remove the claim of “fast and accurate similarity detection” and to avoid implying that the combined use of the suffix array and LCP array constitutes a new insight. The text now briefly explains the role of the enhanced suffix array as a standard indexing structure that enables efficient enumeration of shared substrings and k-mers, and clarifies its computational trade-offs, particularly in terms of memory usage.

This revision reframes the description as methodological background and eliminates any ambiguity regarding its contribution to the novelty of the approach.

[Comment R1-40]- On line 178, "basing" should maybe be "based"?

[Answer] We thank the reviewer for pointing out this grammatical error. We have corrected " basing" to " based" in the revised manuscript.

[Comment R1-41]- On line 183, maybe the "Multiplicity Vector building" phase should really be called "Multiplicity Vector construction", to align with the other -tion names?

[Answer] We thank the reviewer for this suggestion. We have changed the term to 'Multiplicity Vector construction' in the text and updated the corresponding figure (Fig 1) to reflect this change.

[Comment R1-42]- On line 184, "a serial" should probably be "serial". There are many similar minor grammar errors in this section of the text describing Figure 1 which should be corrected.

[Answer] We thank the reviewer for pointing out the minor grammatical errors in the description of Figure 1. We have corrected “a serial” to “serial” and carefully revised the section for grammar and clarity to improve readability throughout the manuscript.

[Comment R1-43]- Why is the multiplicity vector building phase serial, when the multiplicity vectors being built do not depend on each other?

[Answer] Although the Multiplicity Vectors (MVs) are independent for each gene, their construction requires a consistent mapping of k-mers to unique integer IDs via the function f. This mapping is managed through a global shared hash map. We chose to perform this phase serially to populate the map dynamically without the significant performance overhead of synchronization primitives (such as mutexes) and to prevent race conditions. Once all MVs are constructed, the hash map is no longer needed and is discarded, allowing the computationally heavy Best Hit detection and BBH extraction to proceed in a fully parallel, lock-free manner.

We clarified this in the paper by adding “This function relies on a shared hash map to store the association between k-mers and their integer identifiers.

While the MVs for different genes are independent, their construction relies on this shared map, which is dynamically populated as new k-mers are encountered.

This design choice avoids race conditions and the overhead of synchronization mechanisms (e.g., locks) that would be required for concurrent write access to the map.

Once the MVs are built during this serial phase, the hash map is discarded, as it is no longer required for the subsequent parallel comparison phases.”

[Comment R1-44]- Line 188 talks about sorting k-mers, but I think it needs to talk about sorting occurrences of k-mers instead, or the multiplicity information will get lost.

[Answer] We thank the reviewer for pointing out the potential ambiguity regarding the sorting of k-mers. In the original text, it was not clear that the multiplicity information is preserved during sorting. We have clarified that each unique k-mer identifier is paired with its multiplicity, and the resulting pairs are sorted by the k-mer identifier, ensuring that the multiplicity information is maintained for each gene. This change makes explicit that the multiplicity vector retains all relevant information for downstream comparisons.

[Comment R1-45]- In general I expected the association of k-mers with integers to be done using a hash function, or a simple encoding of the k-mer sequence into the bits of an integer. Is it actually being done by numbering the kmers in the order they are encountered, or something? Line 188 talks about "associating" a k-mer with an integer like that requires an action, and the f function from k-mer to integer is not yet defined at the beginning of the algorithm.

[Answer] We appreciate this observation. The function f does not employ a static bit-encoding (which would generate excessively large integers for large k) nor a direct hash value (which would result in a sparse index space). Instead, f maps k-mers to consecutive integers (0,1,2,...,N) based on the order of their first appearance across the dataset. This strategy ensures a dense range of integer IDs, which is crucial for the efficiency of the Multiplicity Vectors. Consequently, the "action" referred to is the lookup-or-insert operation in the global hash map: if a k-mer is new, it is assigned the next available integer ID.

We have revised the definition of f in the "Multiplicity vector construction" section to explicitly describe this incremental assignment process and its relationship with the serial construction phase.

The sentence:

“Consider a function $f(\alpha) : \alpha \to n$, such that $\alpha \in \Gamma^*$ and $n \in \mathbb{N}$, that is used to associate each kmer to a unique positive integer.”

s replace with:

“Consider a function $f(\alpha) : \Gamma^k \to \mathbb{N}$ that assigns a unique, consecutive integer identifier to each distinct k-mer found in the dataset.

This mapping is constructed incrementally based on the order of appearance: the first unique k-mer encountered is assigned 0, the second is assigned 1, and so on.

This approach ensures a dense range of identifiers, minimizing the memory footprint of the resulting vectors.“

[Comment R1-46]- Line 193 would be clearer if it talked about sorting on k-mer number and not on the first element of a pair.

[Answer] We agree that the original phrasing was slightly ambiguous. We have revised the sentence to explicitly state that the sorting is performed based on the k-mer integer identifier (f(α)), rather than referring generically to the data structure's "first element". The sentece:

“The vector is sorted in ascending order according to the first element of the pair.”

Has been replaced with :

“The vector is sorted in ascending order based on the k-mer integer identifier $f(\alpha)$.”

[Comment R1-47]- Line 197 to 200 start talking about rows and columns, but hasn't really explained what matrix these are rows and columns of, and which axis is which.

[Answer] We thank the reviewer for this comment. We have clarified the description of the Best Hit detection phase to explicitly define the similarity matrix: rows correspond to genes in the first genome, and columns correspond to genes in the second genome. Best Hits are identified along each row, and the resulting data are stored in a jagged array. We have also clarified that this matrix and array are used in the subsequent phase to extract Bidirectional Best Hits. This revision ensures that the meaning of "rows" and "columns" is unambiguous and directly tied to the gene comparison data structure.

[Comment R1-48]- After line 201 I expected a description of what's happening in Figure 1 part c, since up until this point we've been following the Figure 1 structure

[Answer] We thank the reviewer for pointing out the lack of an explicit description of Fig. 1c. To improve clarity while avoiding redundancy, we revised the pipeline overview to explicitly state in the opening paragraph of the "PanDelos-plus pipeline overview" section that PanDelos-plus is a redesign of the original PanDelos framework, and that several steps are inherited from the original methodology.

In the revised text, phase (c) of Fig. 1 is now explicitly contextualized as the gene family extraction step based on bidirectional best hits and a network-based refinement procedure, following the original PanDelos approach (as indicated in the manuscript):
"The third phase (c) is used to extract gene families from the bidirectional Best Hit, via a network-based refining procedure according to the original methodology."

Accordingly, we do not provide a detailed re-description of phase (c) later in the section, and instead focus on the in-depth description of phase (b), which represents the main methodological contribution and redesign introduced in PanDelos-plus.

[Comment R1-49]- At line 203 it would help to explain again that we are constructing 1 multiplicity vector for each gene.

[Answer] We thank the reviewer for pointing out this ambiguity. We have revised the text to explicitly state that a unique Multiplicity Vector is constructed for each individual gene. The sentence " The MV is a data structure that all genes will handle in order to store all different k-mers,along with related multiplicities." has been replaced with

"In this phase, a specific MV is constructed for each gene to store all its constituent k-mers, along with their related multiplicities."

[Comment R1-50]- At line 204, "handle" isn't the right word; maybe "that all genes will handle in order to store" should really be "that will store"?

[Answer] We have revised the text to clarify that the multiplicity vector (MV) is a data structure that stores, for each gene, all distinct k-mers along with their associated multiplicities. This change replaces “handle” with the more precise and accurate term “stores,” improving clarity and readability.

[Comment R1-51]- Lines 208 to 209 don't really make sense as written; a "function" by definition always gives equal outputs for equal inputs. This could be cut.

[Answer] We agree with the reviewer and we removed the sentence.

[Comment R1-52]- The equation after line 213 is missing an opening brace. Also, at this point, the equations stop being numbered.

[Answer] We fixed the missing opening brace and added the number to the following equations.

[Comment R1-53]- The description of the sort order at lines 214-215 would probably be something like "is sorted in ascending order by f() value", instead of trying to express it as a mathematical condition.

[Answer] We thank the reviewer for this comment. We have revised the description of the multiplicity vector sort order to indicate that it is sorted in ascending order according to the f() value, rather than expressing the order as a formal mathematical condition. This makes the text clearer and more readable.

[Comment R1-54]- Line 216 should make it clearer that now we're talking about repeating Algorithm 1, not discussing the inputs of Algorithm 1.

[Answer] We have rephrased the sentence to explicitly state that the procedure described in Algorithm 1 is executed iteratively for every gene in the dataset to construct the corresponding Multiplicity Vectors. The sentence:

“ The work defined in Algorithm~\ref{Alg 1} is done for each gene in the genomes G_i and G_j. ”

Has been replaced with:

“ The procedure outlined in Algorithm~\ref{Alg 1} is iteratively executed for every gene g in each genome $G \in \mathbb{G}$ to construct its corresponding MV. ”

[Comment R1-55]- Lines 217-218 maybe should invite the reader to note that there are no string comparisons in the algorithm; as is it sounds like there's some other algorithm, not presented here, which included string comparisons that have now been removed.

[Answer] We agree that the original sentence was confusing and sounded more like a changelog than a description of the method. We have rewritten the sentence to explicitly state that the use of Multiplicity Vectors fundamentally eliminates the need for string comparisons in the subsequent phases, replacing them with more efficient integer operations. The sentence:

“ Any type of computation that could include any type of string comparison has been substituted by a computation on the MV data structure. ”

Has been replaced:

“ It is important to note that once the MVs are constructed, all subsequent computations rely solely on integer operations on these data structures. This approach effectively eliminates the need for computationally expensive string comparisons during the similarity calculation phases. ”

[Comment R1-56]- On line 222, "detect" should be "detects".

[Answer] We thank the reviewer for pointing out this grammatical error. We have corrected " detect " to " detects " in the revised manuscript.

[Comment R1-57]- Lines 233-234 say the matrix row is independent from its corresponding jagged array row, but they are meant to say that the matrix row and its corresponding jagged array row are a unit, and all the units are independent of each other.

[Answer] We thank the reviewer for this comment. We have revised the description to clarify that each row of the similarity matrix and its corresponding jagged array row together form a unit of work, and it is these units that are independent and parallelizable. This correction ensures the text accurately reflects the relationship between the matrix and jagged array and the parallelization strategy.

[Comment R1-58]- On line 236, "unit" should be "units".

[Answer] We thank the reviewer for pointing out this grammatical error. We have corrected " unit " to " units " in the revised manuscript.

[Comment R1-59]- On line 236, instead of saying the units of work are "represented by" matrix rows, it should say something more like "defined as", or just say that the unit of work "is" the matrix row.

[Answer] We revised the paragraph by replacing the term “represented by” with the term “form”.

[Comment R1-60]- On line 239, "rows" should be "row".

[Answer] We thank the reviewer for pointing out this grammatical error. We have corrected " rows " to " row " in the revised manuscript.

[Comment R1-61]- Algorithm 2 must have access to the multiplicity vector information at line 5 to compute the similarity. But the multiplicity vector information isn't listed as an input to the algorithm I also can't find where the similarity function is defined; I think different notation is used in the text. Conversely, the θ and τ filters do not appear in Algorithm 2.

[Answer] We thank the reviewer for this observation. We now specify in the text how the function “similarity” is computed and, in particular, that it calculates J_k similarity between two genes for which

multiplicity vectors have been previously computed and are available as global variables of the program.

[Comment R1-62]- On line 245, I thing the subject of the sentence (which would be "it") is missing. Also "identify" might not be the right word; maybe this should be something like "A Bidirectional Best Hit requires that..."

[Answer] We thank the reviewer for pointing out this grammatical and stylistic issue. We revised the sentence to explicitly introduce the subject and to more clearly define the condition for a Bidirectional Best Hit. In particular, we rephrased the sentence to state that *a Bidirectional Best Hit requires that* the Best Hit relation holds in both directions, improving both grammatical correctness and conceptual clarity.

[Comment R1-63]- Lines 245-246 are talking about Best Hits of rows and columns, rather than of genes. I found thinking about the best hit of a column confusing, having just gotten through the explanation of how we're computing the rows and they're independent.

[Answer] We agree that the original phrasing was confusing. The ambiguity stemmed from an imprecise use of indices, where genome identifiers (i,j) were inadvertently conflated with matrix row and column indices. We have revised the text to consistently refer to Best Hits of *genes* and to explicitly distinguish between genome indices and matrix indices (rows l and columns k).

In addition, we added a clarifying sentence in the Methods section describing the similarity matrix S_{i,j} to make explicit that S_{i,j}[l,k] denotes the similarity between the l-th gene of genome G_i and the k-th gene of genome G_j. This clarification is intended to avoid confusion when referring to rows and columns of the similarity matrix in the Best Hit and Bidirectional Best Hit extraction phases.

[Comment R1-64]- At line 246, "just only" should probably be "only".

[Answer] We corrected the phrase by replacing “just only” with “only”.

We changed the sentence:

“So just only a part of the matrix Si,j is needed to be considered in that phase.”

To:

“Therefore, only a subset of the matrix Si,j needs to be considered in this phase, namely the columns that contain at least one Best Hit of any row.”

[Comment R1-65]- At line 247, and again at line 248, "is needed to be considered" should be "needs to be considered".

[Answer] We agree with the reviewer and have revised the phrasing accordingly. The expression “is needed to be considered” has been replaced with “needs to be considered” in both instances to improve grammatical correctness and readability.

[Comment R1-66]- I found the description of the part of the matrix at lines 248-249 confusing. It sounds like at this point we have the Best Hits in one direction, and now we need to find the Best Hits in the other direction, but only for genes that were themselves Best Hits of something. But Best Hit was defined on genes, and now we're using it on matrix rows and columns (or cells?), and I don't know what it measn to contain a Best Hit "on" the "j-column". (Was that meant to be "j-th column"?)

[Answer] We thank the reviewer for highlighting this ambiguity. We revised this part to clarify that Best Hits are defined between genes, and that rows and columns of the similarity matrix $S_{i,j}$ correspond to genes in genomes G_i and G_j, respectively. In particular, we corrected the index notation to distinguish genome indices (i,j), as per S_{i,j}, from matrix indices (l,z), like S_{i,j}[l,z]. We clarified that the "relevant columns" are those corresponding to genes in G_j that appear in at least one Best Hit of a gene in G_i, as stored in the jagged array $A_{i,j}$.

We also revised the phrasing to remove expressions such as "Best Hit on the j-column" and replaced them with explicit references to the genes represented by the corresponding matrix columns. This makes explicit that the Bidirectional Best Hit extraction step checks reciprocity between gene-level Best Hits.

[Comment R1-67]- On line 251, "contains" should be "contain".

[Answer] We thank the reviewer for pointing out this grammatical error. We have corrected "contains" to "contain" in the revised manuscript.

[Comment R1-68]- On line 257, "independency" should be " independence".

[Answer] We thank the reviewer for pointing out this grammatical error. We have corrected " independency " to " independence " in the revised manuscript.

[Comment R1-69]- Algorithm 3 would be more readable if at Line 2 the comment was wrapped separately and kept on the right side near its bullet point.

[Answer] We thank the reviewer for pointing out this formatting problem, we have reformatted the comment in Algorithm 3, Line 2. It is now wrapped within a fixed-width box aligned to the right, ensuring it remains readable and visually associated with the corresponding instruction.

[Comment R1-70]- Lines 264 to 270 have rampant conflation between sets of experiments, datasets, and sets of genomes. A set of experiments should not be "referred as real dataset" (which, moreover, would be "referred to as"). A set of experiments also can't be "composed of" real bacterial genomes. This section needs to be rewritten to say that the "real-dataset experiments" were run *on* a set of real genomes, and similarly for the synthetic-dataset experiments.

[Answer] We thanks the review for pointing out this logical confusion and the grammar error. We have rewritten the introduction paragraph as follow:

"Two sets of experiments have been conducted to evaluate the performance of PanDelos-plus. The first set, refered as real dataset, is composed of real bacterial genomes, and is used to compare the

performance of PanDelos-plus with the original PanDelos algorithm, and to measure scalability in runtime and memory usage as the thread count grows.

The second set, referred as synthetic dataset, is composed of synthetic bacterial genomes, and is used to measure scalability on large genome collections generated via PANPROVA." becames "Two sets of experiments were conducted to evaluate the performance of PanDelos-plus.

The first set of experiments was performed on a collection of real bacterial genomes, hereinafter referred to as the real dataset. This dataset was used to compare the performance of PanDelos-plus with the original PanDelos algorithm and to measure scalability in runtime and memory usage as the thread count increases.

The second set of experiments was performed on a collection of synthetic bacterial genomes, hereinafter referred to as the synthetic dataset. This dataset was used to measure scalability on large genome collections generated via PANPROVA~\cite{bonnici2022panprova}."

[Comment R1-71]- On line 270, PANPROVA should probably be cited.

[Answer] We thank the reviewer for pointing out this miss citation. We have added the reference for PANPROVA.

[Comment R1-72]- On line 281, "PanDelos original" should be "original PanDelos", and "S1 File" should be "File S1".

[Answer] We have corrected the phrasing to "original PanDelos" and updated the file reference to "File S1", "File S2" and "File S3".

[Comment R1-73]- On line 283-284, "in REFSEQ annotation" doesn't make any sense to me. Are these "from" something called REFSEQ? Are they annotated as being "REFSEQ" genomes? Or is it something else?

[Answer] We have revised the sentence to clarify that the genomes were downloaded from the NCBI RefSeq database, which provides standardized and consistently annotated reference genomes. This removes the unclear phrasing "in REFSEQ annotation" and accurately describes the source and nature of the dataset.

[Comment R1-74]- On line 288, what is an "htg pool"? Is "htg" an acronym? Why not "HTG"? Is this meant to be "HGT" for "Horizontal Gene Transfer"?

[Answer] We corrected the sentence by explicitly stating "horizontal gene transfer pool". We also revised the sentence to improve clarity on using the horizontal gene transfer pool with PANPROVA.

[Comment R1-75]- On line 288, "S2 File" should be "File S2".

[Answer] We corrected the names of the supplementary files through the manuscript.

[Comment R1-76]- On line 289, it's not sufficiently clear that "from 50 to 600" refers to the sizes of the different collections.

[Answer] We apologize for the ambiguity. We have rephrased the text to make it clear that '50 to 600' refers to the specific sizes of the subsets. As detailed in the response to the comment below (regarding lines 289-290), we clarified that these collections were obtained by down sampling a larger master dataset.

[Comment R1-77]- On lines 289-290, it is stated that the collections were made by increasing the number of genomes "at each step" (presumably the steps between collections of adjacent sizes), while "maintaining the previous genomes untouched". That would read better as "keeping the previous genomes untouched", but more importantly I'm baffled as to how this could be accomplished. It looks like PANPROVA doesn't model horizontal transfer *between* branches of the tree being simulated, which would make this completely impossible, but it still seems very difficult. PANPROVA simulates a population of genomes over time, right? How can PANPROVA be run to simulate 100 genomes, but under the constraint that the first 50 it outputs must be exactly the same as on the previous run where it only simulated 50? Is PANPROVA able to hang new genomes it generates on the same true tree that was used to make the existing ones, which gets saved and re-used between runs? Or is there some larger population size that is used during the simulation, and then that population is down sampled to 50, 100, 150, etc. to produce the different collections? If so, why not explain this as simulating 600 genomes and then downsampling to progressively smaller subsets?

[Answer] The reviewer is absolutely correct in their hypothesis: we followed the down sampling approach. To clarify, we did not run separate simulations for each step. Instead, we generated a single 'master' dataset containing 1,000 genomes using PANPROVA. From this master pool, we extracted the 12 experimental subsets (ranging from 50 to 600 genomes). This methodology ensures that the dataset with 100 genomes contains exactly the same genomes as the 50-genome dataset, plus 50 additional ones from the pool. This eliminates any variability artifacts between runs. We have rewritten the text to explicitly describe this down sampling process.

[Comment R1-78]- On line 293, why would we expect there to be artifacts from new genome generation? What would they be like? Maybe it would make more sense to explain this as making sure that all the genomes actually come from the same simulated history so that relationships between them are realistic.

[Answer] We thank the reviewer for pointing out that the reference to “artifacts” was unclear. Our intention was to emphasize that generating each dataset size through independent simulations could introduce variability in evolutionary history, potentially confounding scalability evaluation. We have revised the text to clarify that all experimental collections are derived from a single master simulation, ensuring that they share the same evolutionary background and that scalability is evaluated solely as a function of dataset size.

[Comment R1-79]- In Table 2, why does the minimum number of genes start at 5202 and not decrease as the number of genomes in the collection increases? Did the lowest-gene-count simulated sample just *happen* to end up in the first 50, or is something else going on?

[Answer] The reviewer's intuition is correct. As clarified in the response to the previous comment (regarding lines 289-290), the datasets are constructed using a cumulative (nested) approach: the set of 100 genomes includes the exact set of 50 genomes, and so on.

Consequently, the genome with the minimum number of genes (5,202) happened to be part of the initial batch of 50. Since this specific genome is preserved in all subsequent larger collections, the minimum value remains constant at 5,202 (as no genome smaller than that was encountered in the subsequent additions). This stability is a direct consequence of the cumulative sampling strategy.

[Comment R1-80]- In Table 2, shouldn't the rows be labeled as "Collection 1", etc. instead of "Dataset 1", to match the terminology in the text? Or should different terms be used for all the real data and all the simulated data, when talking about experiments in the text?

[Answer] We agree with the reviewer regarding the terminology inconsistency. We have updated Table 2 to use the term 'Collection' (e.g., Collection 1, Collection 2) instead of 'Dataset'. This aligns with the text and better reflects the nested nature of these genome sets. Furthermore, we have reviewed the entire manuscript and corrected all similar inconsistencies to ensure full terminological uniformity.

[Comment R1-81]- On line 296, "16c" should probably be "16-core".

[Answer] We thank the reviewer for the comment. We removed the "16c" and changed the paragraph to better explain the type of processors used for our experiments and added supplementary information in File S3.

[Comment R1-82]- On line 297, the system used is described as having 32 cores, but these are physical cores with simultaneous multithreading, and so the system has 64 logical cores, right? In general, since CPU manufacturers are known to re-use model numbers, when reporting what CPU was used for performance metrics, one should include all of:

- Model name
- Base clock speed
- L1 and L2 cache size per core
- L3 cache size in total
- Number of sockets, cores, and threads
- If the cores are heterogeneous, the number of each type of core
- Whether hyper-threading was left on or turned off
- Whether turbo boost or similar up-clocking features were left enabled or disabled

[Answer] We thank the reviewer for requesting these detailed specifications. The experiments were conducted on a shared computing node within our university's HPC infrastructure. Using system diagnostics, we have retrieved the exact hardware configuration available to our user partition.

Regarding the core count, we must clarify a detail: while the processor model theoretically supports Simultaneous Multithreading (SMT), the specific configuration of our assigned node has SMT disabled

(reporting 1 thread per core and 32 active cores). Therefore, the experiments ran on 32 physical cores corresponding to 32 logical threads, not 64.

We have updated the manuscript to reflect this configuration and provided a comprehensive table with all the requested metrics (cache sizes, architecture topology, etc.) in the new Supplementary Material S3_File.

[Comment R1-83]- Table 3 should probably include PanDelos-plus on 1 thread, to show that the speedup is coming from the ability to use multiple threads and not a faster single-thread implementation.

[Answer] We agree with the reviewer that comparing PanDelos-plus on a single thread is important to distinguish the contribution of parallelism from that of the redesigned data structures. Rather than modifying Table 3, which is intended to highlight the overall improvement over PanDelos, we have added a new table (Table 4) that reports PanDelos-plus performance at both 1 thread and 32 threads, including wall-clock time, total CPU time, and memory usage.

[Comment R1-84]- Starting around line 306, units like "s" and "GB" should be in Roman (not italic) tyoe, and should be separated from their numbers by a non-breaking space (not immediately adjacent to them).

[Answer] As suggested by the reviewer we changed the typeset of units to Roman and separated them from their value.

[Comment R1-85]- On line 317, the word "significantly" is used, and even though there's not a real empirical comparison being made here, seeing it without a statistical test made me worried.

[Answer] We thank the reviewer for pointing this out. We did not intend to imply statistical significance. The term "significantly" was used in a qualitative sense to indicate a marked improvement. To avoid ambiguity, we have revised the sentence by removing "significantly" and rephrasing it to reflect a descriptive, non-statistical comparison.

[Comment R1-86]- Line 321 talks about "a constant problem size across the same datasets", and ,aybe should talk about "a constant input" instead.

[Answer] We thank the reviewer for this suggestion. While "constant problem size" is standard terminology in strong scaling analysis, we agree that "fixed input dataset" provides clearer wording in this context. We have revised the sentence accordingly.

[Comment R1-87]- Lines 322-327 seem to contain unfinished text redundant with the surrounding text and should be deleted.

[Answer] We thank the reviewer for noting the incomplete and redundant sentence. The fragment has been removed, and the paragraph has been revised to clearly and concisely describe the strong scaling results without repetition.

[Comment R1-88]- The top and bottom panels in Figure 2 (and similar figures) should be assigned (a) and (b) to make them easier to reference as "Fig 2a", etc.

[Answer] Following the reviewer suggestion we assigned (a) and (b) to the figure panels

[Comment R1-89]- The memory usage results in Figure 2 are described as "nearly constant", but using 1 thread used noticeably less memory than using 2 threads.

[Answer] We agree with the reviewer. We changed the wording from "nearly constant" to "overall constant".

[Comment R1-90]- The text shouldn't reference figure panels by title, just by number and letter/position.

[Answer] We thank the reviewer, we changed the reference to the plot as suggested using number and letter.

[Comment R1-91]- Line 336 uses the word "significant" about an empirical result without there being a statistical test.

[Answer] We thank the reviewer, we removed the improperly used "significant" word.

[Comment R1-92]- In the Figure 2 caption, "indicating minimal overhead from parallelization" and "confirming efficient parallel scalability across all datasets" offer unnecessary opinions; the caption shouldn't make these sorts of judgements.

[Answer] We thank the reviewer for the comment. We have revised the figure caption to be purely descriptive, focusing on what is plotted: peak memory usage and execution time as functions of the number of threads, without drawing any conclusions.

[Comment R1-93]- Line 339 has another passive-voice sentence where the subject position is incorrectly empty.

[Answer] We corrected the sentence by changing "have been conducted" to "we conducted" thus switching from passive to active voice

[Comment R1-94]- Line 342 is missing a subject for the "varied" clause.

[Answer] We added "we" as the subject for "varied"

[Comment R1-95]- Line 345 has another similar bad construction at "has been used a constant number of threads".

[Answer] We changed “has been used” to “we used”.

[Comment R1-96]- On line 359, it doesn't make sense to use "about" with a number with 5 significant figures; maybe say "about 9.6 hours", and only then give the value in seconds?

[Answer] As suggested by the reviewer, we removed the use the word “about” and “roughly” with numbers with 5 significant figures. For the number of hours, we used the mathematical symbol for approximation.

[Comment R1-97]- Lines 359-364 really want to be a table, rather than a bunch of pairs of numbers written inside a sentence.

[Answer] We agree with the reviewer. As suggested, we replaced the description of the values with a Table.

[Comment R1-98]- On line 364, "Since the execution with 2 threads" should probably be "Even with only 2 threads".

[Answer] We thank the reviewer for the comment. We followed the suggestion and changed the phrase to be “Even with only 2 threads”.

[Comment R1-99]- On line 361, and in the Figure 3 caption, runtime is said to decline "almost linearly" as the number of threads increases. I think this is wrong for two reasons. First, with perfect parallelization, runtime is not expected to decrease *linearly* with thread count. Going from 1 to 2 threads should remove half the time, but adding an additional thread should not be expected to remove the *other* half of the time and produce a result of 0 time, as you would expect by extrapolating a line from those two points. Instead, we expect that doubling the number of threads should halve the time, which would produce a straight downward-sloping line *on a log-log plot*. Second, I think the text overstates the degree to which the measured scaling follows this theoretical relationship. At 32 threads, we would ideally take 1/32nd of the time as with one thread, but actually we take 1.8/32nds, or almost twice as long as would be required in the ideal case. It's still quite scaleable, but it looks like it might be starting to reach a point of diminishing scaling returns at around 32 threads, and it is neither "almost linear" nor almost ideal.

[Answer] We thank the reviewer for this important clarification. We agree that describing the runtime reduction as “almost linear” was not technically accurate. Under ideal parallelization, runtime is expected to decrease proportionally to the inverse of the thread count, rather than linearly with respect to the number of threads. We have therefore revised the manuscript to state that execution time decreases approximately inversely with thread count, and we explicitly acknowledge that the observed scaling is sub-ideal.

Specifically, we now report that at 32 threads the runtime is reduced by approximately 18x relative to single-threaded execution, compared to the theoretical 32x reduction expected under ideal scaling. The revised text and Figure 3 caption clarify that the implementation demonstrates strong but non-ideal parallel efficiency, with diminishing scaling gains at higher thread counts.

We believe this revision provides a more accurate theoretical framing and a more precise interpretation of the empirical results.

[Comment R1-100]- At lines 370 and 377, the text says something like "The plot (Fig 4, top), titled "...."", which doesn't read right. It should be "Figure 4a", with a real panel letter, or "In the top plot of Figure 4", and the title shouldn't be used.

[Answer] We changed the text as suggested. Now the plots are referenced as Fig. 3a, Fig. 3b, Fig. 4a and Fig. 4b.

[Comment R1-101]- At line 379 and in the figure 4 caption, it is said that execution time grows "sublinearly" with data set size. This is obviously false from looking at the figure, or the numbers. 62 hours at 600 samples is much more than 6 times 2 hours at 100 samples. The note about "indicating near-linear time complexity and effective parallel efficiency even on very large datasets" is false, and also reads like it was sampled from a random distribution (what is "effective parallel efficiency" exactly, other than several good things packed together into a syntactically valid phrase?).

[Answer] We thank the reviewer for pointing out this inconsistency. Upon re-examination of the reported values, we agree that describing the growth in execution time as “sublinear” was incorrect. As the number of genomes increases from 50 to 600 (a 12× increase), runtime grows from approximately 0.5 h to 62 h, corresponding to an increase of more than two orders of magnitude. This clearly reflects superlinear scaling rather than sublinear or near-linear behavior.

We have therefore revised both the main text and the Figure 4 caption to accurately describe the observed trend as superlinear scaling. We have also removed the statements referring to “near-linear time complexity” and “effective parallel efficiency,” as these were not supported by the empirical results and could be interpreted as overstated. The revised text now provides a strictly quantitative and descriptive account of the observed behavior.

We believe the corrected version more faithfully represents the data and avoids any unsupported interpretation.

[Comment R1-102]- On line 386, "enhancement over" should be "improvement over" or maybe "enhancement of".

[Answer] Following the reviewer suggestion we have revised the phrasing to use “enhancement of” for clarity and correctness.

[Comment R1-103]- Lines 399-401 misstate the results again, and saying that the result "highlights its robust scalability" again sounds like pulling words randomly out of a hat. Good scalability in dataset size doesn't "highlight" scalability; it *is* scalability. And what would it mean for scalability to be "robust"? The manuscript doesn't define or do anything to try and measure the "robustness" of scalability; indeed, scalability is only assessed on one series of collections of genomes, from one simulator, so it's possible different ways of getting collections of genomes would somehow show different results.

[Answer] We thank the reviewer for this suggestion. We have revised the conclusion to more accurately reflect the results obtained on the synthetic datasets. Specifically, we now distinguish between memory usage, which grows roughly linearly with the number of genomes, and execution time, which exhibits superlinear growth as the genome collection increases. We also clarify that parallelism provides strong speedup for fixed dataset sizes but does not fully offset the combinatorial increase in computational cost for larger collections. The revised text removes vague terms such as "robust scalability" and focuses on the observed performance, reporting measured speedup, memory requirements, and scaling behavior explicitly. This ensures that the conclusions are fully supported by the data without overgeneralization.

[Comment R1-104]- For the supporting information, "Supplementary material real dataset" and "Supplementary material synthetic dataset" are bad titles; they could be "File S1: Real Dataset" and "File S2: Synthetic dataset"

[Answer] We thank the reviewer for the comment. We followed the suggestion and changed the titles.

[Comment R1-105]- The supporting information for the synthetic dataset experiments does not contain the actual simulated genomes, nor does it contain the exact simulator version and random seed or other information sufficient to re-simulate the same genomes again.

[Answer] We agree with the reviewer that this information are essential for reproducibility. We have updated the example folder in the GitHub repository to include the scripts used to generate the synthetic datasets and to run the experiments presented in the paper. Additionally, a dedicated supplementary file (File S3) has been added reporting detailed hardware and software specifications (including compiler version, optimization flags, and the exact commit hash of PanDelos-plus), as requested by Reviewer 1. File S3 also includes a dedicated section describing the PANPROVA version and configuration used to generate the synthetic datasets.

Finally, I briefly reviewed the PanDelos-plus codebase. Overall, I was happy with it, but the project made some unusual decisions that might benefit from being explained somewhere, maybe in its documentation.

[Comment R1-106]- Almost all of the C++ code is header-only, which is defensible for a project of this size, but which is more commonly seen in libraries than in applications.

[Answer] We acknowledge the reviewer 's observation regarding the header-only structure. However, in our perspective, the header-only approaches facilitates system configuration and integration, avoiding the need for complex build chains or linking setups. In fact, the project size is currently manageable, making the compilation overhead negligible compared to the benefits. Moreover, such an approach ensures the compiler has full visibility over the code units, allowing for maximum optimization opportunities (such as inlining).

[Comment R1-107]- The build system uses a strangely specific combination of optimization flags on top of optimization level 1, instead of the more commonly used optimization level 3 which I think would turn on most of the relevant flags anyway. Are there particular optimizations that the authors

discovered to be counterproductive during their experimentation, which are deliberately being left out? Or particular optimizations they found to be useful that aren't in the base level 3 preset for the compiler they use?

[Answer] We thank the reviewer for pointing out this aspect. The optimization profile was determined empirically during development by selectively enabling individual optimization passes and evaluating their impact on correctness and performance. All benchmarks reported in the paper were executed under the same compilation profile, ensuring a fair and consistent comparison across all experiments. The build configuration adopts a granular optimization strategy. Instead of relying on the generic -O3 preset, we utilized -O1 as a stable baseline and explicitly enabled specific high-impact optimizations targeting loop transformations, inter-procedural analysis, and modulo scheduling, while avoiding aggressive inlining and auto-vectorization. The flag -fno-lifetime-dse is required for correctness given the memory management patterns in the codebase. All benchmarks reported in the paper were executed under this configuration. We have included all the details in the S3 Supplementary file.

[Comment R1-108]- If the particular set of optimizations is important, it might be a good idea to report the compiler version used in the manuscript, as well as the commit hash or released version number of PanDelos-plus that was tested.

[Answer] We agree with the reviewer. We have added a dedicated table in the supplementary material (File S3, Table 2) reporting the compiler version, the optimization profile used, and the specific commit hashes of both PanDelos-plus and PANPROVA.

[Comment R1-109]- The recommended way to run PanDelos-plus is through Docker Compose, which is used to simplify the command-line command and automatically mount input and output directories into the tool's container. This does work, but it seems rather unusual, because Docker Compose is really designed to orchestrate several containers together to provide a persistent service, rather than to run a single container to completion.

[Answer] We thank the reviewer for this insightful observation. We agree that Docker Compose is primarily designed for multi-container orchestration rather than single-container execution. Based on this feedback, we have restructured the Docker documentation to offer two methods:

- Method A: Docker run (recommended) - We now provide a wrapper script (run-docker.sh) and direct docker run commands as the standard approach. This aligns with conventional Docker usage for single-execution tools.
- Method B: Docker Compose - Retained as an alternative for users who prefer declerative configuration files.
- The Dockerfile now includes an ENTRYPOINT directive, allowing users to run the tool with a simple command:

The Dockerfile now includes an ENTRYPOINT directive, allowing users to run the tool with a simple command:

```
docker run --rm \
  -v "$(pwd)/input:/home/pdp/PanDelos-plus/input" \
  -v "$(pwd)/output:/home/pdp/PanDelos-plus/output" \
```

```
  pandelosplus -i input/file.pdi -o output/result
```

Or using the provided wrapper script:

```
./run-docker.sh -i input/file.pdi -o output/result
```

If the user prefer can use a directory with gbff files with docker command:

```
docker run --rm \
  -v "$(pwd)/input:/home/pdp/PanDelos-plus/input" \
  -v "$(pwd)/output:/home/pdp/PanDelos-plus/output" \
  pandelosplus -i input/custom.pdi -o output/custom -g input/gbff/
```

Or with wrapper script:

```
./run-docker.sh -i input/custom.pdi -o output/custom -g input/gbff/
```

[Comment R1-110]- The Dockerfile for PanDelos-plus does not build the version of the repository that one has checked out, because it retrieves the source code for the desired version from Github and uses that instead. But, it uses the pip-requirements.txt file from the local repository, not from the requested version, to determine what Python dependencies to install. This makes it overly complicated to build a particular version of the tool, because you need to both check it out *and* provide the version number to the Docker build process.

[Answer] We completely agree with the reviewer's assessment. The previous Docker build strategy, which mixed local configuration files with remote source code fetching, was indeed inconsistent and prone to version mismatches. We have resolved this issue by refactoring the Dockerfile.

[Comment R1-111]- Much of the tool's user interface appears to be localized in both Italian and English, which is good to see in scientific software written for a broad audience. However, the Italian messages are not accessible to end-users; they are turned on only in debug builds of the program. The program should instead respect the environment variables configuring locale and language, probably by using the setlocale() function to read them, and select between Italian and English messages depending on the language that the user has configured at runtime.

[Answer] We agree with the reviewer's observation. The Italian messages were originally intended solely for internal debugging purposes during development. We have updated the codebase to use English-only messages throughout.

Reviewer #2: In the manuscript entitled “PanDelos-plus: A parallel algorithm for computing sequence homology in pangenomic analysis,” Colli et al. introduce an updated, parallelized implementation of the previously published PanDelos and PanDelos-frags pangenome tools. The improvements include redesigned internal data structures, multi-threaded BH/BBH computation, and substantially reduced memory usage. These scalability enhancements are valuable for large bacterial pangenomes.

[Comment R2-1]However, the claims of methodological novelty are overstated relative to PanDelos (Bonnici et al., 2018) and PanDelos-frags (Bonnici et al., 2023). The core algorithmic framework, k-mer multiplicity vectors, generalized Jaccard similarity, and BH/BBH-based homology inference, remains unchanged, and the primary advances are engineering and parallelization improvements.

[Answer] We agree with the reviewer that the core algorithmic framework (k-mer multiplicity vectors, generalized Jaccard similarity, BH/BBH-based homology inference) remains unchanged from PanDelos. This is by design, as PanDelos has been shown to outperform other solutions in terms of result quality. However, the engineering and parallelization improvements presented in PanDelos-plus are not merely incremental: they enable the application of the PanDelos methodology to substantially larger inputs that were previously infeasible, which in turn can yield more informative pangenomic reconstructions. To demonstrate this concretely, we have added an analysis of gene family diffusivity across the real datasets of increasing size and show of the relative diffusivity of families change with the input size. The new methodology can more effectively refine the aggregation of genes into families by leveraging homology information from an increased number of analysed genomes. In fact, the classification of a specific family as core, dispensable, or singleton is sensibly affected by the availability of such information.

[Comment R2-2]Additionally, the performance comparisons in Table 3 present speedups in a way that may mislead readers regarding per-core efficiency. The authors themselves acknowledge this limitation in lines 393–397: “Importantly, the analysis of synthetic datasets shows that while the single-threaded version of PanDelos remains faster than PanDelos-plus when only one core is used, the parallel design of PanDelos-plus allows it to surpass PanDelos already with two threads, achieving nearly ideal scalability as the number of threads increases.” A clearer presentation of single-core versus multi-core performance would strengthen the benchmarking section.

[Answer] We agree with the reviewer that a clearer distinction between single-core and multi-core performance strengthens the benchmarking section. To address this concern, and also in response to a similar request by Reviewer 1, we have introduced a new table (Table 4) that reports PanDelos-plus performance at both 1 thread and 32 threads, including wall-clock time, total CPU time, and memory usage. This table clearly shows that the single-threaded execution of PanDelos-plus is slower than PanDelos on the real datasets, confirming that the observed speedups in Table 3 are attributable to parallel execution rather than to a faster sequential implementation. Table 3 has been retained to provide a direct comparison between PanDelos and PanDelos-plus at their respective intended usage configurations (single-threaded vs 32 threads).

[Comment R2-3]Furthermore, the abstract describes PanDelos-plus as a “gene-centric” redesign of PanDelos, and the Methods section defines genomes strictly as lists of gene sequences. However, the manuscript does not explicitly state that PanDelos-plus does not operate on full genome assemblies or raw sequence data, and that users must supply pre-annotated gene sequences. This is a critical

practical limitation of the tool. I recommend adding an explicit statement early in the Methods, and ideally in the Introduction, clarifying that PanDelos-plus accepts gene sequences rather than genome assemblies as input and does not include gene prediction or genome reconstruction functionality.

[Answer] We thank the reviewer for highlighting this important clarification. PanDelos-plus operates on pre-annotated gene sequences and does not perform genome assembly, gene prediction, or genome reconstruction from raw sequencing data. We have added a paragraph in the methodology and clarify the statement in the Introduction to make this practical limitation clear. We specify that input genomes may originate from complete assemblies, draft assemblies, or metagenome-assembled genomes, provided that gene annotation has been performed prior to analysis. Additionally, for users working with unannotated genomes, we are working on a gene prediction pipeline in the repository, based on the preprocessing workflow originally developed for PanDelos-frags, which allows users to obtain annotated gene sequences suitable as input for PanDelos-plus. This version of PanDelos-frags, available will be avaiable on PanDelos-plus repository's organization (https://github.com/synbionics).

[Comment R2-4]Overall, the work appears to represent a valuable parallelization and optimization extension, but not a conceptual or methodological advance over prior versions of PanDelos. Strengthening the framing of novelty and clarifying input requirements would improve the manuscript.

[Answer] Following the reviewer's suggestion, we revised the manuscript to strengthen the framing of novelty. Specifically, we clarified that PanDelos-plus is not merely a parallelized or optimized version of the original PanDelos. While it improves computational efficiency, its ability to handle much larger genome collections also has direct implications for the biological results. By enabling the inclusion of more genomes in a single analysis, PanDelos-plus allows for a more accurate classification of gene families into core, dispensable, and singleton categories, reflecting their true distribution across the sampled population rather than being limited by small sample size.

We emphasize this point in the introduction: the classification of gene families depends inherently on the number and diversity of genomes analyzed. As additional genomes are incorporated, families previously considered singletons may be reclassified as dispensable if homologs are detected in newly added genomes. This illustrates that PanDelos-plus is not just faster; it enables a more precise characterization of pangenome structure and dynamics, which is particularly important for species with open pangenomes, where gene discovery continues to increase with sampling. Therefore, the novelty of our work lies in both computational scaling and the resulting improvement in the biological insight that can be obtained from large genomic datasets.

To further substantiate the novelty and biological relevance of PanDelos-plus, we conducted additional analyses . These tests compare gene family diffusivity between pangenomes reconstructed from a Reduced subset of genomes and the Complete genome collection. The results demonstrate that scaling the number of genomes primarily leads to additive expansion of gene families, the emergence of novel families, and redistribution across diffusivity classes, while preserving the structural integrity of previously inferred clusters. This confirms that PanDelos-plus enables not only faster computation but also a more accurate characterization of gene family frequencies and pangenome dynamics when larger genome collections are analyzed.

Moreover, we acknowledge that the core algorithmic framework (k-mer multiplicity vectors, generalized Jaccard similarity, BH/BBH-based homology inference) is inherited from PanDelos. However, we would like to point out that the contribution of PanDelos-plus extends beyond a

straightforward parallelization effort. The replacement of the enhanced suffix array with a lightweight multiplicity vector data structure represents a novel engineering solution: to the best of our knowledge, no comparable structure in the literature provides equivalent advantages for parallel k-mer similarity computation with such a reduced memory footprint.

Lastly, we clarified the input requirements in the methodology section.

Reviewer #3: Manuscript presents PanDelos-plus, a new version of a tool for identifying homologous gene families called PanDelos. The improvement concerns computational efficiency – reduced memory consumption and parallelization.

The manuscript contains several unsubstantiated claims, which seem to indicate that the authors are exaggerating the results of their work, which in reality seem minor. In particular:

[Comment R3-1]1. The authors claim that the memory reduction results from replacing enhanced suffix arrays (ESA) with some lightweight data structures. However, a straightforward (not commpressed) ESA implementation requires less than 10 bytes per character, which yields the memory consumption very similar to the memory used in the experiments by PanDelos-plus. Thus it seems that a more thorough ESA implementation would do be adequate.

[Answer] We appreciate the reviewer's technical observation regarding the memory requirements of Enhanced Suffix Arrays (ESA). While we acknowledge that a compressed ESA could be memory-efficient, the primary motivation for shifting away from the suffix structure was to prioritize runtime performance in a multi-threaded environment. Implementing a compressed structure would introduce a significant computational overhead due to the compression and decompression cycles required for data access. In a high-performance parallel context, the constant need to decompress data on-the-fly creates a processing bottleneck (latency) that outweighs the memory benefits. In contrast, the Multiplicity Vector (MV) structure maintains a direct, uncompressed representation of k-mer counts, allowing for immediate access and efficient computation of the generalized Jaccard similarity without the CPU burden of decoding.
We revised the paper to clear such aspects.

[Comment R3-2]2. The authors claim that the PanDelos-plus computation time scales almost linearly (in line 379, they even write "sublinearly") with respect to the number of genomes. In fact, the results presented in Figure 4 reveal a near-quadratic relationship - increasing the input data by a factor of 12 results in a time increase of over 100.

[Answer] We agree that the original characterization of the scaling behavior as sublinear (and near-linear) was incorrect. We have revised the manuscript to remove these claims and now describe the observed trend in purely empirical terms. Specifically, the revised text states that increasing the number of genomes by a factor of 12 results in an execution-time increase of roughly a factor of 100, with progressively larger increments as dataset size grows, indicating superlinear scaling. This correction aligns the description with the data shown in Fig. 4 and avoids attributing an unsupported complexity class.

[Comment R3-3]3. In the abstract, the authors state "Benchmarks on synthetic datasets show that PanDelos-plus achieves (...), while maintaining accuracy", but no accuracy assessment was performed.

[Answer] We agree with the reviewer the term accuracy is not suitable for this case. We changed the sentence by using the term “consistent”. The meaning is that both algorithms produce the same output.

Moreover, the 'Materials and methods' section is terribly written. For example:

[Comment R3-4]l.70: do you really assume that each genome has exactly m genes?

[Answer] We have revised the notation to allow each genome Gi to have a variable number of genes m_i, denoted as G_i = {g_{i,1}, g_{i,2}, ..., g_{i,m_i}} where m_i =|Gi| is the (variable) number of genes in genome G_i . This modification accurately reflects that different genomes may contain different numbers of genes and removes the implicit assumption of uniform genome size in the original formulation.

[Comment R3-5]l.73: it is better to reformulate: "The substring of a sequence s=... is denoted by s[y,w]."

[Answer] We have revised the sentence to clearly define a substring of a sequence s and denote it as s[y,w], specifying the start and end positions for clarity.

[Comment R3-6]l.76-77: this sentence is completely incomprehensible

[Answer] We agree with the reviewer. We have rewritten this passage to provide a precise and unambiguous definition of a k-mer and to clearly specify the relationship between the sequence length and the number of substrings of length k.

[Comment R3-7]l.81 and l.84: unnecessarily repeated definition of substring from l.73
formula (1): this defines the sets of (k+1)-mers, not k-mers

[Answer] We removed the unnecessary repeated definition of substring as suggested. We corrected the formula (1) to correctly define the set of k-mers and not (k+1)-mers

[Comment R3-8]l.90: in what sense this is the best k?
[Answer] We clarified why such a value is the best one by modifying the paragraph. Specifically, we added this sentence: “In fact, according to the studies reported in [22, 23], such a value for k is the most informative resolution for comparing real sequences with random ones.”

[Comment R3-9]l.101: Word "therefore" suggests that previous sentence explains the motivation for the criteria, but it does not. The actual motivation seems to be included in the next paragraph, but this arrangement confuses the reader.

[Answer] We removed the word “therefore” and added an introductory paragraph of the pre-filtering steps before the definition of Jaccard similarity. With this arrangement, the reader should be clear.

[Comment R3-10]l.119: What do you mean "ensures" here? Explain how it ensures.
formula (7) and l.122: occ_s(w) is a set according to formula (2), arithmetic operations are not applicable

[Answer] We thank the reviewer for identifying this inconsistency. We have corrected formula (7) by replacing $occ_s(w)$ with $\mult_s(w)$, which denotes the multiplicity (i.e., the count of occurrences) of k-mer w in sequence s, as defined in Eq. (3). This ensures that the arithmetic operations in the formula are applied to scalar values rather than to sets. The accompanying text has been updated accordingly.

[Comment R3-11]l.176: do you mean "revision"?

[Answer] We thank the reviewer for the suggestion. We have replaced the term with "redesign" to better clarify the structural evolution of the pipeline.

[Comment R3-12]l.187-188: do you assign all possible k-mers to the same integer?

[Answer] The answer is no. So, we modified the sentence in order to be clearer.

[Comment R3-13]l.206: Why should the reader "consider" function f? If you mean a particular function, give it's definition.

[Answer] We gave a description for f, in particular, we clarified that f exploits the use of an hashmap for mapping repeated k-mers into a unique integer.

[Comment R3-14]l.208-209: I have no idea, what property this sentence is supposed to describe. If a_1=a_3 then f(a_1)=f(a_3) for any function f and no matter what a_2 is.

[Answer] We agree with the reviewer, the previous formulation was unclear. We wanted to formally define that the function acts as a perfect hashing, assigning unique integer identifiers to distinct k-mers (collision-free) while maintaining consistency for identical k-mers. We have rewritten the sentence to clarify that distinct k-mers occurring in the input genes map to distinct integers.

The previous sentence:

"Given α_1, α_2 and α_3, such that $\alpha_1,\ \alpha_2, \alpha_3 \in \Gamma^*$ with $\alpha_1 \ne \alpha_2$ and $\alpha_1 = \alpha_3$, the function ensures that $f(\alpha_1) = f(\alpha_3)$ whenever $f(\alpha_1) \neq f(\alpha_2)$"

has been rewritten as:

"Let α_1, α_2 and α_3 be k-mers occurring in the input genes,

such that, $\alpha_1 \ne \alpha_2$ and $\alpha_1 = \alpha_3$, the function ensures that

$f(\alpha_1) = f(\alpha_3)$ and $f(\alpha_1) \neq f(\alpha_2)$. This function relies on a shared hash map to store the association between k-mers and their integer identifiers."

[Comment R3-15]l.245-246: the notion of the best hit of a row/column is misleading - best hits should be identified with row/column indexes rather than matrix entries

[Answer] We agree with the reviewer that the previous notation was potentially confusing, as referring to rows and columns using the genome indices (i and j) was ambiguous. We have revised the text to strictly use indices l and k when referring to rows (genes of G_i) and columns (genes of G_j) respectively.
