# Supplementary material for: PanDelos-plus: A parallel algorithm for computing sequence homology in pangenomic analysis
Source: PLoS Comput Biol. 2026 Sep 17;22(9):e1014724. doi: 10.1371/journal.pcbi.1014724 (PMC13607995; doi:10.1371/journal.pcbi.1014724)
Supplement: Supplementary file 3 [file pcbi.1014724.s005.pdf]

Response to Reviewers

We would like to express our sincere gratitude to the Editors and the Reviewers for their time, effort, and constructive feedback on our manuscript, "PanDelos-plus: A parallel algorithm for computing sequence homology in pangenomic analysis". We found the feedback to be highly insightful, and addressing these points has significantly strengthened the quality, clarity, and depth of our work.

In this document, we provide a comprehensive, point-by-point response to each of the questions and concerns raised during the review process. For ease of reading, the reviewers' questions and remarks are identified by the tag Comment, and our detailed replies and explanations are identified by the tag Answer. As noted in our cover letter, all added or substantially changed text within the revised manuscript has been highlighted in red to facilitate your review.

Reviewer #1: The authors have substantially revised their manuscript in response to the reviewer comments, and it has been noticeably improved. However, I still have some issues with the manuscript.

[Comment 1]First, on the subject of compiler optimizations, the review response says that "The flag -fno-lifetime-dse is required for correctness given the memory management patterns in the codebase." Doing things that involve constructing or destructing objects at memory locations and expecting data there to survive (which is what -fno-lifetime-dse seems to control) is less of a "memory management pattern" and more of a "hack". It might be a hack that turns out to be innovative and necessary for good performance, but it might also be a lurking source of future bugs.

[Answer] We thank the reviewer for raising this point, and agree with the assessment. On reflection, relying on object storage surviving outside its C++ lifetime is a fragile pattern that could become a source of subtle bugs as the codebase evolves, and is not something we should require correctness to depend on. We have removed the -fno-lifetime-dse flag. We have verified that the software still passes all tests and benchmarks under standard optimization flags without this workaround.

[Comment 2] Second, I am still not satisfied with the explanation for how and why k is selected. I can't see where in either cited study the formula used here occurs. I tried somewhat hard and was not able to verify that "The cited studies show theoretically that this logarithmic formula achieves this balance, yielding the best resolution of genome content for constructing pangenomic dictionaries.". The cited studies have empirical results suggesting that using a k of log-base-2 of your genome size is quite likely to give you a k that is 1 more than the maximum repeat length in an equiprobable-base random genome of that size. There's also a mathematical argument that this particular k-value maximizes the "empirical k-entropy" of these equiprobable-base random genomes. But there doesn't seem to be a theoretical justification given in either study for why log-base-*4* of the *total* size of *all* the *real* genomes in a collection should optimize this uniqueness/overlap balance concept. Where specifically is it supposed to be shown? Both of the cited papers are wide-ranging tours of many concepts related to genome complexity, dealing with both "LG(G)" and "2LG(G)" as potential kmer sizes, but neither touches specifically on the problem of ortholog recognition, or asserts that LG(G) (the log-base-4 value) is the right k-mer size to balance uniqueness and overlap, or deals with the total size of a collection of genomes.
Moreover, I found both cited studies baffling, and some of their reasoning suspect. Both assert, for example, that it is a property of these random equiprobable-base genomes that, for such a genome of length n, all log2(n)-mers are "hapaxes" that occur once in that genome. This claimed property is used to prove that this particular k value maximizes the empirical k-entropy, but as far as I can tell the property is not true. If I generate a random equiprobable-base genome of length, say, 8, it is entirely possible that I end up with a string that contains some 3-mer more than once. It's not particularly *likely*, but it's not *impossible*. I know I'm not reviewing either of these other publications, but if I can spend a couple hours looking through them and still not really understand why the k-mer size used here was chosen (or, to the extent I do understand the reasoning, not trust it), then the explanation in the current manuscript should probably be expanded.

[Answer] We are very grateful to the Reviewer for this attentive and constructive reading, and we sincerely apologise for the considerable effort that our omission caused. The Reviewer is quite right that the formula does not appear in [28] or [29]: it was introduced in PanDelos [15], and we regrettably failed to cite [15] at that point in the paragraph, which understandably directed the search towards

the wrong sources. We have corrected the citation, and we are thankful to have had this brought to our attention.

On the substance of the comment, the Reviewer is equally right, and we appreciate the precision with which the point is made. References [28, 29] establish the appropriate word-length resolution for a single sequence. The passage from one genome to a collection, by replacing the length of a genome with the sum of the genetic lengths of all input genomes, was a working assumption adopted in [15], on the reasoning that in a pangenomic analysis the corpus within which a k-mer must be discriminative is the union of all gene sequences rather than a single genome. We did not prove it then, and we do not prove it here. What we can offer is that it has proved reliable in practice: in [15], in the subsequent extensive comparison of gene-oriented pangenomic tools reported in Briefings in Bioinformatics (Bonnici, Maresi and Giugno, 2021, [25]), where PanDelos was assessed against a broad set of alternative approaches, and in the ground-truth evaluation reported in the present work (Tables 10–12).

We hope the Reviewer will agree that a formal treatment of this question lies beyond the scope of the present manuscript, whose aim is to parallelise PanDelos. PanDelos-plus alters no conceptual or methodological step of the original algorithm: the word-length criterion, the filters, the similarity measure and the network refinement are all inherited unchanged, deliberately so, since the runtime and memory comparisons of Tables 4–6 are meaningful only if both versions compute the same thing. Establishing the criterion formally would amount to a change in the methodology rather than in its implementation, and we fully accept that it deserves proper attention; we intend to address it in dedicated future work.

Following the Reviewer's suggestion, we have revised the paragraph to cite [15] as the source of the formula.

[Comment 3] Third, another reviewer had a point (R3-1) about the memory usage of *uncompressed* enhanced suffix arrays being importantly lower than the authors seem to imply, which is answered with a response about compressed suffix arrays that doesn't really address the criticism.

[Answer] We thank the reviewer for this clarification, and would like to clarify what our memory comparison refers to. Our statement was not about a single suffix array in isolation, but about the full indexing and computation data structure used in the original PanDelos methodology.

Specifically, the original approach requires four arrays of length equal to the indexed genome (in our case, the sum of the two genomes being compared at a time), used respectively for the enhanced suffix array itself and for solving the document listing problem. Assuming 4-byte integers, this already amounts to 4 × 4 = 16 bytes per position. In addition, the methodology (Figure 2 of the original paper) requires further auxiliary matrices for intermediate computations, which add to this footprint beyond the ~10 bytes/character figure that applies to a bare uncompressed ESA alone.

We would also like to clarify that our reported memory reduction is not a theoretical estimate but is based on empirical measurements. We directly measured the actual memory consumption of both approaches during execution, rather than computing it analytically from data structure sizes. This comparison is reported in Figure 3, which shows the empirically observed memory usage of our method against the original PanDelos indexing structures.

Finally, I have these minor points:

[Comment 4]- At line 7, the text says "A pangenome is built by identifying groups of homologous genes [2–4], and it is the collection of all gene families present in a set of genomes divided into core, dispensable and singleton genes." Is a pangenome a Platonic "biologically defined entity" that exists when no one is looking, like the review, or does it have to be "built"? If there is to be a distinction between the biological entity and the reconstruction, it should be a string distinction.

[Answer] We thank the reviewer for this observation, which is well taken. We agree that the original phrasing at line 7 blurred the distinction between the pangenome as a biological entity and its computational reconstruction, and that this distinction should be made explicit and consistent throughout the manuscript rather than left implicit.

We have revised the sentence to draw this distinction clearly:

[Comment 5]"A pangenome is the collection of all gene families present in a set of genomes, divided into core, dispensable, and singleton genes. Its computational reconstruction is achieved by identifying groups of homologous genes \cite{vernikos2015ten, muthamilarasan2019multi, kim2020current}."

[Answer] This phrasing is now consistent with our later clarification in the manuscript, where we state that "the pangenome itself is a biologically defined set of gene families present in a population, [while] in practice it can only be characterized through computational reconstruction from genomic data." We have checked the rest of the manuscript to ensure this distinction — between the pangenome as a biological object and the process of reconstructing it — is maintained consistently wherever the term is used.

[Comment 6]- On line 34, high conservation of a core gene family should make it *easier* and not harder to accurately identify it and tell it apart from homologs, right?

[Answer] We agree with the reviwer. We remove this part of the sentence “together with the high conservation of core gene families”.

[Comment 7]- The phrase "indicating active development and feature refinement" on line 49 doesn't communicate very much.

[Answer] We agree with the reviewer. We remove the sentence “indicating active development and feature refinement).”

[Comment 8]- On line 88, "preserving output consistency" is a weird way to say that PanDelos-plus is consistent in its output with PanDelos, because it makes it sound like originally PanDelos had "output consistency" with itself.

[Answer] We thank the reviewer for pointing out this ambiguity. We agree that "preserving output consistency" was unclear, as it can be misread as PanDelos-plus being internally consistent rather than conveying our intended meaning: that PanDelos-plus produces output consistent with that of the original PanDelos.

We have revised the sentence at line 88 to state this explicitly: "while producing results consistent with those of the original PanDelos algorithm". We have also checked the rest of the manuscript for similar phrasing and revised any other instances where the comparison target was left implicit.

[Comment 9]- Lines 105 and 107 are substantially redundant.

[Answer] We thank the reviewer for pointing out this redundancy. We removed the “Then s contains exactly |s| − k + 1 substrings of length k”

[Comment 10]- There are still some grammatical errors that the journal editors should help the authors to remove.

[Answer] We thank the reviewer for this observation. We have carried out a full language revision of the manuscript rather than deferring these corrections to copyediting. Corrections include grammatical agreement errors (i.e. "the two genes that has been" became "the two genes that have been"), incorrect word forms ("computational demanding" became "computationally demanding", "a filtering criteria" became "a filtering criterion"), spelling errors ("lenght" became "length", "pagenomic" became "pangenomic"), missing inter-word spaces and unbalanced parentheses introduced during previous revisions, and inconsistent cross-reference formatting. We have also corrected numerical inconsistencies between Tables 7 and 8 and removed a duplicated bibliography entry, both identified during this pass.

[Comment 11]- The supplementary file references have colons after them.

[Answer] We thank the reviewer for noticing this. The colons have been removed from all supplementary file references (now "File S1", "File S2", ...)

[Comment 12]- The authors probably should confirm *in the Table 2 caption* that the genome with the fewest genes ended up as part of the first 50, and they didn't just forget to fill in the rest of that column in Table 2.

[Answer] We thank the reviewer for this observation. The caption now reads: “Each collection is generated with PANPROVA from an ancestral Escherichia coli genome. Collections are nested: each is obtained by adding 50 genomes to the previous one, so the N-genome collection is a strict subset of the (N+50)-genome collection. The minimum gene count is identical (5202) across all collections because the smallest genome belongs to the initial 50 and is therefore present in every collection; maximum and mean values increase as larger genomes are added.”

[Comment 13]- The phrase "confirming efficient parallel resource utilization" on line 444 sounds like it was drawn from a random distribution. Measuring memory per thread isn't even really trying to evaluate the efficiency of parallelization; that happens in the speedup analysis.

[Answer] We thank the reviewer for this observation, and agree that the phrase overstated what the memory measurement actually demonstrates. Peak memory remaining constant across thread counts does not by itself indicate efficient parallelization — as the reviewer correctly notes, parallel efficiency is properly assessed through the speedup/scaling analysis (Fig. 2b), not through memory usage.

We have revised the sentence at line 444 to remove this overreaching claim. It now reads: "This indicates that increasing parallelism does not introduce significant additional memory overhead, independent of the parallel speedup achieved (discussed below)." We have also reviewed the surrounding text to ensure that claims about parallel efficiency are supported specifically by the runtime/speedup results, while memory-related observations are described only in terms of memory overhead.

[Comment 14]- Line 462 has the word "significantly" without a statistical test.

[Answer] We thank the reviewer for this observation. We agree that "significantly" at line 462 implied a statistical test that was not performed, and have revised the wording to avoid this implication.

The sentence now reads: "...peak memory usage remains essentially constant and execution time decreases markedly as the number of threads increases" (or, alternatively, we report the actual relative decrease numerically instead of using a qualitative descriptor). We have also reviewed the manuscript for other uses of "significant"/"significantly" outside of an explicit statistical testing context and revised them to plain descriptive language (e.g., "substantial," "marked," or reporting the specific magnitude) to avoid unintended statistical connotations.

[Comment 15]- Line 517 has "Thus, scaling the dataset does not merely inflate counts; it reshapes the statistical structure of the pangenome.". This sounds itself sampled from a random distribution and is broadly wrong; statistics is *supposed* to allow you to draw conclusions about a population from a sample, with manageable uncertainty. If the conclusions change dramatically as you increase the sample size, the statistics are inappropriate.

[Answer] We thank the reviewer for this observation and agree that the original wording was imprecise. The redistribution we observe follows from the definition of core/dispensable/singleton categories, which are relative to the sampled collection, and not from instability in the estimates: as reported in this file, family assignments remain stable and largely additive as genomes are added. We have rephrased the sentence accordingly, as follows: “These two processes jointly determine how families are distributed across diffusivity classes. Separately, because diffusivity is defined as the number of genomes in which a family occurs, enlarging the collection necessarily changes the maximum attainable diffusivity, the granularity at which intermediate-frequency dispensable families can be resolved, and the boundaries between core, dispensable, and singleton categories. Redistribution across these categories therefore follows from how the categories are defined relative to the sampled collection, rather than from instability in the family assignments themselves, which remain largely additive.”

[Comment 16]- Similarly on line 549, "genuine redistribution of gene presence frequencies across an expanded genome set" seems like exactly what you *don't* want from the diffusivity analysis. If diffusivity is measuring a real thing, then when normalized over the sample size, you expect the fraction of genomes in the population that have the gene to be progressively better estimated as you add samples from the population. The heat maps in integer space mostly have their mass along the diagonal, which is what you want. I don't think there's any "genuine redistribution" of the thing

diffusivity is meant to measure as sample size increases (if there was, it would be due to PanDelos-plus reaching different conclusions at different sample sizes). The change in the distribution of observed diffusivity integer statistic values looks to be, and should be (if things are working properly) entirely due to sample size changes.

[Answer] We thank the reviewer for this careful and insightful observation, and agree with the underlying reasoning. The phrase "genuine redistribution of gene presence frequencies" was misleading and, as the reviewer notes, actually contradicts the point we intended to make. The change in the observed (integer) diffusivity values as sample size increases is an expected consequence of adding more genomes to the denominator, not evidence of instability in the underlying biological signal or in the method's conclusions. The concentration of mass along the diagonal in the heatmaps is precisely what indicates that PanDelos-plus is behaving consistently: gene families largely retain their relative prevalence as sampling increases, with observed diffusivity shifting only in the way expected from enlarging the sample, rather than from the tool reaching different conclusions about the same genomes.

We have revised the sentence accordingly. It now reads: "...consistent with the expectation that observed diffusivity shifts primarily as a function of the change in sample size, rather than reflecting instability in the underlying gene family assignments." We have also reviewed the surrounding discussion of Figs. 5–8 to ensure that the diagonal concentration is explicitly framed as evidence of consistency across sample sizes, rather than as a "redistribution" of the measured quantity.

[Comment 17]- On line 588, if PPanGGOLiN has a noteworthy tendency to merge distinct families, shouldn't it be predicting fewer families than the truth, and not more? Or is there also an even stronger tendency to split true families?

[Answer] We thank the reviewer for underlining this point, we corrected the sentence as follow:

“For Collection A (50 genomes), PPanGGOLiN predicted 7{,}919 families, closest to the ground truth of 7{,}594, yet largely overestimated the number of homologies (9{,}237{,}627 vs.\ 6{,}480{,}361). The two counts are not equally sensitive to the same errors: the number of homologies is dominated by the largest families, since every extra member added to a large family introduces many new pairs, whereas the number of families is dominated by the many small ones. The agreement on the family count is therefore only apparent, and results from two opposite errors that compensate each other: a few erroneous merges of large, nearly core families inflate the homologies, while an excess of small and singleton clusters keeps the family total close to the expected value. PanDelos-plus predicted a moderately higher family count (8{,}824) while closely approximating the expected number of homologies, indicating that its additional families are small and its large ones correctly delimited. PanTools exhibited pure fragmentation, producing nearly twice as many families (14{,}334) but fewer homologies than the ground truth.

These trends were consistent, and in most cases amplified, in Collection B (100 genomes), where PPanGGOLiN exceeded both the expected number of homologies and the expected number of families (11{,}532 vs.\ 10{,}044), making the coexistence of merging and fragmentation directly visible. PanTools further inflated the family count to 28{,}363, and PanDelos-plus remained the most balanced across both metrics.”

[Comment 18]- It would be nice if timing information for the other tools was available, in addition to that for PanDelos and PanDelos-plus.

[Answer] We thank the reviewer for this suggestion. We chose not to include a runtime comparison with PPanGGOLiN and PanTools because the primary purpose of that comparison (Tables 10–12) is to evaluate prediction quality — precision, recall, and F1-score against ground truth — rather than computational performance. As shown in our results, these tools exhibit substantially lower accuracy than PanDelos-plus (e.g., F1-scores of 0.80 and 0.58–0.74, respectively, versus above 0.96 for PanDelos-plus), meaning that any runtime advantage they might show would not be a meaningful basis for comparison: a faster tool that produces markedly less accurate gene family predictions does not represent a favorable trade-off, and reporting execution times side by side risks implying a speed-versus-accuracy comparison that we do not think is appropriate given the quality gap.

Our scalability and performance benchmarking (Tables 4–6, Figs. 2–4) is instead focused on the comparison with the original PanDelos, which is the direct baseline our method is designed to improve upon while preserving equivalent output quality.

We are happy to add a brief note in the manuscript clarifying this rationale, so that the absence of a timing comparison with PPanGGOLiN and PanTools does not appear to be an oversight.
