# Supplementary material for: PanDelos-plus: A parallel algorithm for computing sequence homology in pangenomic analysis
Source: PLoS Comput Biol. 2026 Sep 17;22(9):e1014724. doi: 10.1371/journal.pcbi.1014724 (PMC13607995; doi:10.1371/journal.pcbi.1014724)
Supplement: Peer review history

# Peer review: Decision Letter 0

**DOI:** 10.1371/journal.pcbi.1014724.r001

**Authors:** Michael Domaratzki, Jean Fan

13 Jan 2026

PanDelos-plus: A parallel algorithm for computing sequence homology in pangenomic analysis

PLOS Computational Biology

Dear Dr. Bonnici,

Thank you for submitting your manuscript to PLOS Computational Biology. After careful consideration, we feel that it has merit but does not fully meet PLOS Computational Biology's publication criteria as it currently stands. Therefore, we invite you to submit a revised version of the manuscript that addresses the points raised during the review process.

Please submit your revised manuscript by Mar 15 2026 11:59PM. If you will need more time than this to complete your revisions, please reply to this message or contact the journal office at ploscompbiol@plos.org. When you're ready to submit your revision, log on to https://www.editorialmanager.com/pcompbiol/ and select the 'Submissions Needing Revision' folder to locate your manuscript file.

Please include the following items when submitting your revised manuscript:

* A letter that responds to each point raised by the editor and reviewer(s). You should upload this letter as a separate file labeled 'Response to Reviewers'. This file does not need to include responses to formatting updates and technical items listed in the 'Journal Requirements' section below.

* A marked-up copy of your manuscript that highlights changes made to the original version. You should upload this as a separate file labeled 'Revised Manuscript with Track Changes'.

* An unmarked version of your revised paper without tracked changes. You should upload this as a separate file labeled 'Manuscript'.

If you would like to make changes to your financial disclosure, competing interests statement, or data availability statement, please make these updates within the submission form at the time of resubmission. Guidelines for resubmitting your figure files are available below the reviewer comments at the end of this letter

We look forward to receiving your revised manuscript.

Kind regards,

Michael Domaratzki

Academic Editor

PLOS Computational Biology

Jean Fan

Section Editor

PLOS Computational Biology

**Journal Requirements:**

1) Please ensure that the CRediT author contributions listed for every co-author are completed accurately and in full.

At this stage, the following Authors/Authors require contributions: Vincenzo Bonnici. Please ensure that the full contributions of each author are acknowledged in the "Add/Edit/Remove Authors" section of our submission form.

The list of CRediT author contributions may be found here: https://journals.plos.org/ploscompbiol/s/authorship#loc-author-contributions

2) Please amend your detailed Financial Disclosure statement. This is published with the article. It must therefore be completed in full sentences and contain the exact wording you wish to be published.

State the initials, alongside each funding source, of each author to receive each grant. For example: "This work was supported by the National Institutes of Health (####### to AM; ###### to CJ) and the National Science Foundation (###### to AM)."

State what role the funders took in the study. If the funders had no role in your study, please state: "The funders had no role in study design, data collection and analysis, decision to publish, or preparation of the manuscript.".

If you did not receive any funding for this study, please simply state: u201cThe authors received no specific funding for this work.u201d

**Reviewers' comments:**

Reviewer's Responses to Questions

**Comments to the Authors:**

**Please note here if the review is uploaded as an attachment.**

Reviewer #1: Review is uploaded as an attachment.

Reviewer #2: In the manuscript entitled “PanDelos-plus: A parallel algorithm for computing sequence homology in pangenomic analysis,” Colli et al. introduce an updated, parallelized implementation of the previously published PanDelos and PanDelos-frags pangenome tools. The improvements include redesigned internal data structures, multi-threaded BH/BBH computation, and substantially reduced memory usage. These scalability enhancements are valuable for large bacterial pangenomes.

However, the claims of methodological novelty are overstated relative to PanDelos (Bonnici et al., 2018) and PanDelos-frags (Bonnici et al., 2023). The core algorithmic framework, k-mer multiplicity vectors, generalized Jaccard similarity, and BH/BBH-based homology inference, remains unchanged, and the primary advances are engineering and parallelization improvements. Additionally, the performance comparisons in Table 3 present speedups in a way that may mislead readers regarding per-core efficiency. The authors themselves acknowledge this limitation in lines 393–397: “Importantly, the analysis of synthetic datasets shows that while the single-threaded version of PanDelos remains faster than PanDelos-plus when only one core is used, the parallel design of PanDelos-plus allows it to surpass PanDelos already with two threads, achieving nearly ideal scalability as the number of threads increases.” A clearer presentation of single-core versus multi-core performance would strengthen the benchmarking section.

Furthermore, the abstract describes PanDelos-plus as a “gene-centric” redesign of PanDelos, and the Methods section defines genomes strictly as lists of gene sequences. However, the manuscript does not explicitly state that PanDelos-plus does not operate on full genome assemblies or raw sequence data, and that users must supply pre-annotated gene sequences. This is a critical practical limitation of the tool. I recommend adding an explicit statement early in the Methods, and ideally in the Introduction, clarifying that PanDelos-plus accepts gene sequences rather than genome assemblies as input and does not include gene prediction or genome reconstruction functionality.

Overall, the work appears to represent a valuable parallelization and optimization extension, but not a conceptual or methodological advance over prior versions of PanDelos. Strengthening the framing of novelty and clarifying input requirements would improve the manuscript.

Reviewer #3: Manuscript presents PanDelos-plus, a new version of a tool for identifying homologous gene families called PanDelos. The improvement concerns computational efficiency – reduced memory consumption and parallelization.

The manuscript contains several unsubstantiated claims, which seem to indicate that the authors are exaggerating the results of their work, which in reality seem minor. In particular:

1. The authors claim that the memory reduction results from replacing enhanced suffix arrays (ESA) with some lightweight data structures. However, a straightforward (not commpressed) ESA implementation requires less than 10 bytes per character, which yields the memory consumption very similar to the memory used in the experiments by PanDelos-plus. Thus it seems that a more thorough ESA implementation would do be adequate.

2. The authors claim that the PanDelos-plus computation time scales almost linearly (in line 379, they even write "sublinearly") with respect to the number of genomes. In fact, the results presented in Figure 4 reveal a near-quadratic relationship - increasing the input data by a factor of 12 results in a time increase of over 100.

3. In the abstract, the authors state "Benchmarks on synthetic datasets show that PanDelos-plus achieves (...), while maintaining accuracy", but no accuracy assessment was performed.

Moreover, the 'Materials and methods' section is terribly written. For example:

l.70: do you really assume that each genome has exactly m genes?

l.73: it is better to reformulate: "The substring of a sequence s=... is denoted by s[y,w]."

l.76-77: this sentence is completely incomprehensible

l.81 and l.94: unnecessarily repeated definition of substring from l.73

formula (1): this defines the sets of (k+1)-mers, not k-mers

l.90: in what sense this is the best k?

l.101: Word "therefore" suggests that previous sentence explains the motivation for the criteria, but it does not. The actual motivation seems to be included in the next paragraph, but this arrangement confuses the reader.

l.119: What do you mean "ensures" here? Explain how it ensures.

formula (7) and l.122: occ_s(w) is a set according to formula (2), arithmetic operations are not applicable

l.176: do you mean "revision"?

l.187-188: do you assign all possible k-mers to the same integer?

l.206: Why should the reader "consider" function f? If you mean a particular function, give it's definition.

l.208-209: I have no idea, what property this sentence is supposed to describe. If a_1=a_3 then f(a_1)=f(a_3) for any function f and no matter what a_2 is.

l.245-246: the notion of the best hit of a row/column is misleading - best hits should be identified with row/column indexes rather than matrix entries

**********

**Have the authors made all data and (if applicable) computational code underlying the findings in their manuscript fully available?**

The PLOS Data policy requires authors to make all data and code underlying the findings described in their manuscript fully available without restriction, with rare exception (please refer to the Data Availability Statement in the manuscript PDF file). The data and code should be provided as part of the manuscript or its supporting information, or deposited to a public repository. For example, in addition to summary statistics, the data points behind means, medians and variance measures should be available. If there are restrictions on publicly sharing data or code —e.g. participant privacy or use of data from a third party—those must be specified.

Reviewer #1: Yes

Reviewer #2: Yes

Reviewer #3: **No:**  The manuscript does not contain any link to the PanDelos-plus code.

**********

PLOS authors have the option to publish the peer review history of their article (what does this mean?). If published, this will include your full peer review and any attached files.

If you choose “no”, your identity will remain anonymous but your review may still be made public.

**Do you want your identity to be public for this peer review?**  For information about this choice, including consent withdrawal, please see our Privacy Policy.

Reviewer #1: No

Reviewer #2: No

Reviewer #3: No

[NOTE: If reviewer comments were submitted as an attachment file, they will be attached to this email and accessible via the submission site. Please log into your account, locate the manuscript record, and check for the action link "View Attachments". If this link does not appear, there are no attachment files.]

**Figure resubmission:**

**Reproducibility:**

?>

# Author comment: Author response to Decision Letter 1

**DOI:** 10.1371/journal.pcbi.1014724.r002

6 Mar 2026

Attachment Submitted filename: response_to_reviewers.pdf

# Peer review: Decision Letter 1

**DOI:** 10.1371/journal.pcbi.1014724.r003

**Authors:** Can Yang

6 Jul 2026

PCOMPBIOL-D-25-02217R1

PanDelos-plus: A parallel algorithm for computing sequence homology in pangenomic analysis

PLOS Computational Biology

Dear Dr. Bonnici,

Thank you for submitting your manuscript to PLOS Computational Biology. After careful consideration, we feel that it has merit but does not fully meet PLOS Computational Biology's publication criteria as it currently stands. Therefore, we invite you to submit a revised version of the manuscript that addresses the points raised during the review process.

Please submit your revised manuscript by Sep 05 2026 11:59PM. If you will need more time than this to complete your revisions, please reply to this message or contact the journal office at ploscompbiol@plos.org. When you're ready to submit your revision, log on to https://www.editorialmanager.com/pcompbiol/ and select the 'Submissions Needing Revision' folder to locate your manuscript file.

Please include the following items when submitting your revised manuscript:

* A letter that responds to each point raised by the editor and reviewer(s). You should upload this letter as a separate file labeled 'Response to Reviewers'. This file does not need to include responses to formatting updates and technical items listed in the 'Journal Requirements' section below.

* A marked-up copy of your manuscript that highlights changes made to the original version. You should upload this as a separate file labeled 'Revised Manuscript with Track Changes'.

* An unmarked version of your revised paper without tracked changes. You should upload this as a separate file labeled 'Manuscript'.

If you would like to make changes to your financial disclosure, competing interests statement, or data availability statement, please make these updates within the submission form at the time of resubmission. Guidelines for resubmitting your figure files are available below the reviewer comments at the end of this letter.

As the corresponding author, your ORCID iD is verified in the submission system and will appear in the published article. PLOS supports the use of ORCID, and we encourage all coauthors to register for an ORCID iD and use it as well. Please encourage your coauthors to verify their ORCID iD within the submission system before final acceptance, as unverified ORCID iDs will not appear in the published article. Only the individual author can complete the verification step; PLOS staff cannot verify ORCID iDs on behalf of authors.

We look forward to receiving your revised manuscript.

Kind regards,

Can Yang

Section Editor

PLOS Computational Biology

Can Yang

Section Editor

PLOS Computational Biology

**Additional Editor Comments (if provided):**

**Journal Requirements:**

If the reviewer comments include a recommendation to cite specific previously published works, please review and evaluate these publications to determine whether they are relevant and should be cited. There is no requirement to cite these works unless the editor has indicated otherwise.

1) Your manuscript is missing the following sections: Discussion.  Please ensure all required sections are present and in the correct order. Make sure section heading levels are clearly indicated in the manuscript text, and limit sub-sections to 3 heading levels. An outline of the required sections can be consulted in our submission guidelines here:

https://journals.plos.org/ploscompbiol/s/submission-guidelines#loc-parts-of-a-submission

2) Please amend your detailed Financial Disclosure statement. This is published with the article. It must therefore be completed in full sentences and contain the exact wording you wish to be published.

i) State the initials, alongside each funding source, of each author to receive each grant. For example: "This work was supported by the National Institutes of Health (####### to AM; ###### to CJ) and the National Science Foundation (###### to AM)."

ii) State what role the funders took in the study. If the funders had no role in your study, please state: "The funders had no role in study design, data collection and analysis, decision to publish, or preparation of the manuscript."

iii) If any authors received a salary from any of your funders, please state which authors and which funders..

If you did not receive any funding for this study, please simply state: u201cThe authors received no specific funding for this work.u201d

**Reviewers' comments:**

Reviewer's Responses to Questions

**Comments to the Authors:**

**Please note here if the review is uploaded as an attachment.**

Reviewer #1: The authors have substantially revised their manuscript in response to the reviewer comments, and it has been noticeably improved. However, I still have some issues with the manuscript.

First, on the subject of compiler optimizations, the review response says that "The flag -fno-lifetime-dse is required for correctness given the memory management patterns in the codebase." Doing things that involve constructing or destructing objects at memory locations and expecting data there to survive (which is what -fno-lifetime-dse seems to control) is less of a "memory management pattern" and more of a "hack". It might be a hack that turns out to be innovative and necessary for good performance, but it might also be a lurking source of future bugs.

Second, I am still not satisfied with the explanation for how and why k is selected.

I can't see where in either cited study the formula used here occurs. I tried somewhat hard and was not able to verify that "The cited studies show theoretically that this logarithmic formula achieves this balance, yielding the best resolution of genome content for constructing pangenomic dictionaries.". The cited studies have empirical results suggesting that using a k of log-base-2 of your genome size is quite likely to give you a k that is 1 more than the maximum repeat length in an equiprobable-base random genome of that size. There's also a mathematical argument that this particular k-value maximizes the "empirical k-entropy" of these equiprobable-base random genomes. But there doesn't seem to be a theoretical justification given in either study for why log-base-*4* of the *total* size of *all* the *real* genomes in a collection should optimize this uniqueness/overlap balance concept. Where specifically is it supposed to be shown? Both of the cited papers are wide-ranging tours of many concepts related to genome complexity, dealing with both "LG(G)" and "2LG(G)" as potential kmer sizes, but neither touches specifically on the problem of ortholog recognition, or asserts that LG(G) (the log-base-4 value) is the right k-mer size to balance uniqueness and overlap, or deals with the total size of a collection of genomes.

Moreover, I found both cited studies baffling, and some of their reasoning suspect. Both assert, for example, that it is a property of these random equiprobable-base genomes that, for such a genome of length n, all log2(n)-mers are "hapaxes" that occur once in that genome. This claimed property is used to prove that this particular k value maximizes the empirical k-entropy, but as far as I can tell the property is not true. If I generate a random equiprobable-base genome of length, say, 8, it is entirely possible that I end up with a string that contains some 3-mer more than once. It's not particularly *likely*, but it's not *impossible*. I know I'm not reviewing either of these other publications, but if I can spend a couple hours looking through them and still not really understand why the k-mer size used here was chosen (or, to the extent I do understand the reasoning, not trust it), then the explanation in the current manuscript should probably be expanded.

Third, another reviewer had a point (R3-1) about the memory usage of *uncompressed* enhanced suffix arrays being importantly lower than the authors seem to imply, which is answered with a response about compressed suffix arrays that doesn't really address the criticism.

Finally, I have these minor points:

- At line 7, the text says "A pangenome is built by identifying groups of homologous genes [2–4], and it is the collection of all gene families present in a set of genomes divided into core, dispensable and singleton genes." Is a pangenome a Platonic "biologically defined entity" that exists when no one is looking, like the review, or does it have to be "built"? If there is to be a distinction between the biological entity and the reconstruction, it should be a string distinction.

- On line 34, high conservation of a core gene family should make it *easier* and not harder to accurately identify it and tell it apart from homologs, right?

- The phrase "indicating active development and feature refinement" on line 49 doesn't communicate very much.

- On line 88, "preserving output consistency" is a weird way to say that PanDelos-plus is consistent in its output with PanDelos, because it makes it sound like originally PanDelos had "output consistency" with itself.

- Lines 105 and 107 are substantially redundant.

- There are still some grammatical errors that the journal editors should help the authors to remove.

- The supplementary file references have colons after them.

- The authors probably should confirm *in the Table 2 caption* that the genome with the fewest genes ended up as part of the first 50, and they didn't just forget to fill in the rest of that column in Table 2.

- The phrase "confirming efficient parallel resource utilization" on line 444 sounds like it was drawn from a random distribution. Measuring memory per thread isn't even really trying to evaluate the efficiency of parallelization; that happens in the speedup analysis.

- Line 462 has the word "significantly" without a statistical test.

- Line 517 has "Thus, scaling the dataset does not merely inflate counts; it reshapes the statistical structure of the pangenome.". This sounds itself sampled from a random distribution and is broadly wrong; statistics is *supposed* to allow you to draw conclusions about a population from a sample, with manageable uncertainty. If the conclusions change dramatically as you increase the sample size, the statistics are inappropriate.

- Similarly on line 549, "genuine redistribution of gene presence frequencies across an expanded genome set" seems like exactly what you *don't* want from the diffusivity analysis. If diffusivity is measuring a real thing, then when normalized over the sample size, you expect the fraction of genomes in the population that have the gene to be progressively better estimated as you add samples from the population. The heat maps in integer space mostly have their mass along the diagonal, which is what you want. I don't think there's any "genuine redistribution" of the thing diffusivity is meant to measure as sample size increases (if there was, it would be due to PanDelos-plus reaching different conclusions at different sample sizes). The change in the distribution of observed diffusivity integer statistic values looks to be, and should be (if things are working properly) entirely due to sample size changes.

- On line 588, if PPanGGOLiN has a noteworthy tendency to merge distinct families, shouldn't it be predicting fewer families than the truth, and not more? Or is there also an even stronger tendency to split true families?

- It would be nice if timing information for the other tools was available, in addition to that for PanDelos and PanDelos-plus.

**********

**Have the authors made all data and (if applicable) computational code underlying the findings in their manuscript fully available?**

The PLOS Data policy requires authors to make all data and code underlying the findings described in their manuscript fully available without restriction, with rare exception (please refer to the Data Availability Statement in the manuscript PDF file). The data and code should be provided as part of the manuscript or its supporting information, or deposited to a public repository. For example, in addition to summary statistics, the data points behind means, medians and variance measures should be available. If there are restrictions on publicly sharing data or code —e.g. participant privacy or use of data from a third party—those must be specified.

Reviewer #1: Yes

**********

PLOS authors have the option to publish the peer review history of their article (what does this mean?). If published, this will include your full peer review and any attached files.

If you choose “no”, your identity will remain anonymous but your review may still be made public.

**Do you want your identity to be public for this peer review?**  For information about this choice, including consent withdrawal, please see our Privacy Policy.

Reviewer #1: No

[NOTE: If reviewer comments were submitted as an attachment file, they will be attached to this email and accessible via the submission site. Please log into your account, locate the manuscript record, and check for the action link "View Attachments". If this link does not appear, there are no attachment files.]

**Figure resubmission:**

**Reproducibility:**

To enhance the reproducibility of your results, we recommend that authors of applicable studies deposit laboratory protocols in protocols.io, where a protocol can be assigned its own identifier (DOI) such that it can be cited independently in the future. Additionally, PLOS ONE offers an option to publish peer-reviewed clinical study protocols. Read more information on sharing protocols at https://plos.org/protocols?utm_medium=editorial-email&utm_source=authorletters&utm_campaign=protocols

# Author comment: Author response to Decision Letter 2

**DOI:** 10.1371/journal.pcbi.1014724.r004

4 Aug 2026

Attachment Submitted filename: answers_to_reviewers.pdf

# Peer review: Decision Letter 2

**DOI:** 10.1371/journal.pcbi.1014724.r005

**Authors:** Can Yang

17 Aug 2026

Dear Dr. Bonnici,

We are pleased to inform you that your manuscript 'PanDelos-plus: A parallel algorithm for computing sequence homology in pangenomic analysis' has been provisionally accepted for publication in PLOS Computational Biology.

Before your manuscript can be formally accepted you will need to complete some formatting changes, which you will receive in a follow up email. A member of our team will be in touch with a set of requests.

Please note that your manuscript will not be scheduled for publication until you have made the required changes, so a swift response is appreciated.

IMPORTANT: The editorial review process is now complete. PLOS will only permit corrections to spelling, formatting or significant scientific errors from this point onwards. Requests for major changes, or any which affect the scientific understanding of your work, will cause delays to the publication date of your manuscript.

Should you, your institution's press office or the journal office choose to press release your paper, you will automatically be opted out of early publication. We ask that you notify us now if you or your institution is planning to press release the article. All press must be co-ordinated with PLOS.

Thank you again for supporting Open Access publishing; we are looking forward to publishing your work in PLOS Computational Biology.

Best regards,

Can Yang

Section Editor

PLOS Computational Biology

Can Yang

Section Editor

PLOS Computational Biology

***********************************************************

Reviewer's Responses to Questions

**Comments to the Authors:**

**Please note here if the review is uploaded as an attachment.**

Reviewer #1: The authors have sufficiently addressed the review comments.

I still have some recommendations that the authors might want to consider when working with the editor to prepare their manuscript for publication:

- Lines 114 (about the theoretical basis for choosing k) and 223 (about k being determined non-empirically) seem to me to contradict line 124 (about how the formula used for k has been empirically shown to generalize to the current problem).

- The new lines 444-446 seem to say about the same thing as lines 449-450.

- On line 613, "directly visible" might not be the right phrase, because here we are looking at summary statistics and not "directly" at the families or at counts of the different error types.

- On line 677, "derade" does not appear to be the word the authors meant to use.

- In some places, the authors write in a style I've come to associate with text that has been through a language model. (For example, see line 633, where "highlighting" is used where I would choose "indicating" or "illustrating", or the phrases, since removed, previously criticized as sounding like they came from a random distribution.) This is fine (though readers might not always prefer reading this style), and, moreover, as people read more and more non-human-written text, this style of writing will often appear even in text that people typed by hand. But I went and checked the PLoS Comp Bio policy page, and it turns out that if any text in the article actually did come through a language model, PLoS policies at https://journals.plos.org/ploscompbiol/s/ethical-publishing-practice#loc-artificial-intelligence-tools-and-technologies would require a section in the methods section about that, when the article format has a methods section. (That policy as written doesn't make much sense to me, because something like "I used Grammarly to reword awkward phrases in my manuscript into idiomatic English" is about the article preparation process, not the actual analysis methods that are of interest to someone looking to repeat the experiments. But it does appear to be the current journal policy.) The authors should work with the editor to ensure that they have whatever is officially required by the journal for all the tools they used in preparing their manuscript.

**********

**Have the authors made all data and (if applicable) computational code underlying the findings in their manuscript fully available?**

The PLOS Data policy requires authors to make all data and code underlying the findings described in their manuscript fully available without restriction, with rare exception (please refer to the Data Availability Statement in the manuscript PDF file). The data and code should be provided as part of the manuscript or its supporting information, or deposited to a public repository. For example, in addition to summary statistics, the data points behind means, medians and variance measures should be available. If there are restrictions on publicly sharing data or code —e.g. participant privacy or use of data from a third party—those must be specified.

Reviewer #1: Yes

**********

PLOS authors have the option to publish the peer review history of their article (what does this mean?). If published, this will include your full peer review and any attached files.

If you choose “no”, your identity will remain anonymous but your review may still be made public.

**Do you want your identity to be public for this peer review?**  For information about this choice, including consent withdrawal, please see our Privacy Policy.

Reviewer #1: No

# Editor report: Acceptance letter

**DOI:** 10.1371/journal.pcbi.1014724.r006

**Authors:** Can Yang

PCOMPBIOL-D-25-02217R2

PanDelos-plus: A parallel algorithm for computing sequence homology in pangenomic analysis

Dear Dr Bonnici,

I am pleased to inform you that your manuscript has been formally accepted for publication in PLOS Computational Biology. Your manuscript is now with our production department and you will be notified of the publication date in due course.

The corresponding author will soon be receiving a typeset proof for review, to ensure errors have not been introduced during production. Please review the PDF proof of your manuscript carefully, as this is the last chance to correct any errors. Please note that major changes, or those which affect the scientific understanding of the work, will likely cause delays to the publication date of your manuscript.

Soon after your final files are uploaded, unless you have opted out, the early version of your manuscript will be published online. The date of the early version will be your article's publication date. The final article will be published to the same URL, and all versions of the paper will be accessible to readers.

For Research, Software, and Methods articles, you will receive an invoice from PLOS for your publication fee after your manuscript has reached the completed accept phase. If you receive an email requesting payment before acceptance or for any other service, this may be a phishing scheme. Learn how to identify phishing emails and protect your accounts at https://explore.plos.org/phishing.

Thank you again for supporting PLOS Computational Biology and open-access publishing. We are looking forward to publishing your work!

With kind regards,

Janani Seenivasan

PLOS Computational Biology | Carlyle House, Carlyle Road, Cambridge CB4 3DN | United Kingdom ploscompbiol@plos.org | Phone +44 (0) 1223-442824 | ploscompbiol.org | @PLOSCompBiol
